# Adenovirus 5-Vectored *P. falciparum* Vaccine Expressing CSP and AMA1. Part A: Safety and Immunogenicity in Seronegative Adults

**DOI:** 10.1371/journal.pone.0024586

**Published:** 2011-10-07

**Authors:** Martha Sedegah, Cindy Tamminga, Shannon McGrath, Brent House, Harini Ganeshan, Jennylynn Lejano, Esteban Abot, Glenna J. Banania, Renato Sayo, Fouzia Farooq, Maria Belmonte, Nalini Manohar, Nancy O. Richie, Chloe Wood, Carole A. Long, David Regis, Francis T. Williams, Meng Shi, Ilin Chuang, Michele Spring, Judith E. Epstein, Jose Mendoza-Silveiras, Keith Limbach, Noelle B. Patterson, Joseph T. Bruder, Denise L. Doolan, C. Richter King, Lorraine Soisson, Carter Diggs, Daniel Carucci, Sheetij Dutta, Michael R. Hollingdale, Christian F. Ockenhouse, Thomas L. Richie

**Affiliations:** 1 U.S. Military Malaria Vaccine Program, Naval Medical Research Center, Silver Spring, Maryland, United States of America; 2 U.S. Military Malaria Vaccine Program, Walter Reed Army Institute of Research, Silver Spring, Maryland, United States of America; 3 Clinical Research Management, Hinckley, Ohio, United States of America; 4 Henry M. Jackson Foundation for the Advancement of Military Medicine, Rockville, Maryland, United States of America; 5 Laboratory of Malaria and Vector Research, National Institute of Allergy and Infectious Diseases (NIAID)/National Institutes of Health (NIH), Rockville, Maryland, United States of America; 6 National Naval Medical Center, Bethesda, Maryland, United States of America; 7 Division of Medical, Audio, Visual, Library and Statistical Services, Walter Reed Army Institute of Research, Silver Spring, Maryland, United States of America; 8 Allied Technology Group Inc., Rockville, Maryland, United States of America; 9 GenVec, Inc. Gaithersburg, Maryland, United States of America; 10 USAID, Washington, D. C., United States of America; 11 USMMVP, Malaria Department, NMRC, Silver Spring, Maryland, United States of America; New York University School of Medicine, United States of America

## Abstract

**Background:**

Models of immunity to malaria indicate the importance of CD8+ T cell responses for targeting intrahepatic stages and antibodies for targeting sporozoite and blood stages. We designed a multistage adenovirus 5 (Ad5)-vectored *Plasmodium falciparum* malaria vaccine, aiming to induce both types of responses in humans, that was tested for safety and immunogenicity in a Phase 1 dose escalation trial in Ad5-seronegative volunteers.

**Methodology/Principal Findings:**

The NMRC-M3V-Ad-PfCA vaccine combines two adenovectors encoding circumsporozoite protein (CSP) and apical membrane antigen-1 (AMA1). Group 1 (n = 6) healthy volunteers received one intramuscular injection of 2×10∧10 particle units (1×10∧10 each construct) and Group 2 (n = 6) a five-fold higher dose. Transient, mild to moderate adverse events were more pronounced with the higher dose. ELISpot responses to CSP and AMA1 peaked at 1 month, were higher in the low dose (geomean CSP = 422, AMA1 = 862 spot forming cells/million) than in the high dose (CSP = 154, p = 0.049, AMA1 = 423, p = 0.045) group and were still positive at 12 months in a number of volunteers. ELISpot depletion assays identified dependence on CD4+ or on both CD4+ and CD8+ T cells, with few responses dependent only on CD8+ T cells. Intracellular cytokine staining detected stronger CD8+ than CD4+ T cell IFN-γ responses (CSP p = 0.0001, AMA1 p = 0.003), but similar frequencies of multifunctional CD4+ and CD8+ T cells secreting two or more of IFN-γ, TNF-α or IL-2. Median fluorescence intensities were 7–10 fold higher in triple than single secreting cells. Antibody responses were low but trended higher in the high dose group and did not inhibit growth of cultured *P. falciparum* blood stage parasites.

**Significance:**

As found in other trials, adenovectored vaccines appeared safe and well-tolerated at doses up to 1×10∧11 particle units. This is the first demonstration in humans of a malaria vaccine eliciting strong CD8+ T cell IFN-γ responses.

**Trial Registration:**

ClinicalTrials.gov NCT00392015

## Introduction

Sterile protective immunity against malaria can be induced in animals or human volunteers by radiation-attenuated *Plasmodium* sporozoites [1], which invade the host hepatocyte but cannot develop into blood stage parasites [2], [3]. Protection is thought to be mediated primarily by interferon-gamma (IFN-γ) secretion by CD8+ and probably also CD4+ T cells recognizing parasite proteins expressed on the surface of infected hepatocytes, with anti-sporozoite antibodies contributing to protection [4], [5], [6]. Humans can also acquire anti-malaria immunity through natural exposure, after repeated episodes of parasitemia. This acquired immunity limits parasite density and clinical disease and appears to be mediated by antibodies to blood stage parasites [7], with cell mediated immunity (CMI) contributing [8], [9]. These findings suggest that a vaccine inducing both cell and antibody-mediated immunity targeting multiple pre-erythrocytic and blood stage antigens could solidly protect humans against malaria.

Viral vectors, used singly or in heterologous prime-boost combination, may constitute a suitable platform for inducing multiple immune responses against multiple parasite stages [10], [11] [12]. In particular, their ability to stimulate CD8+ T cell responses could improve on the partial protection afforded in humans by single antigen, protein-based vaccines such as RTS,S, which elicits strong antibody responses [13], [14], [15], moderate CD4+ T cell responses [16], [17], but no appreciable CD8+ T cell responses [18]. Recombinant adenoviruses, for example, have induced protection against malaria and other infectious agents in mice [19], [20], [21], [22], eliciting high titer antibody [22] and IFN-γ responses [23], [24] including T cell effector memory phenotype, and elevated CD8+ T cell responses including multifunctional responses [25]. To establish proof of principle for this approach, we selected a replication incompetent, serotype 5 adenovirus (Ad5) to construct two adenovectors expressing malaria proteins for human testing. Ad5 enters dendritic cells via the CAR receptor [26], while transduction of hepatocytes and Kupffer cells likely involves a different pathway associated with heparin sulfate proteoglycans [27], [28]. In contrast, Ad35, a less prevalent alternative to Ad5, targets CD46 [27], [29].

The two-component NMRC-M3V-Ad-PfCA vaccine was developed jointly by the US Military Malaria Vaccine Program, GenVec, Inc and USAID. The circumsporozoite protein (CSP) was chosen as a pre-erythrocytic stage test antigen because of its protective role in the RTS,S vaccine [30], and the apical membrane antigen-1 (AMA1) [31] was chosen as the erythrocytic stage test antigen because of protection seen in animal studies [32] and the association with clinical immunity in humans in endemic areas [33]. AMA1 is also expressed in sporozoites and late liver stages [34], and could potentially contribute to protective immunity against pre-erythrocytic stages. Recently a virosomal vaccine containing the repeat structure of CSP and loop 1 of domain III of AMA1 has elicited antibodies in humans that inhibited sporozoite invasion of hepatocytes *in vitro* and induced lymphocyte proliferative responses to AMA1 [35], [36], with no evidence of immune interference by either peptide.

Two clinical studies were performed. In the first, we tested the safety, tolerability and immunogenicity of low and high doses of the two-component NMRC-M3V-Ad-PfCA vaccine in healthy Ad5 seronegative adults (Groups 1 and 2). In the second, we tested the safety, tolerability, immunogenicity and efficacy of the CSP component alone, given in two doses to both Ad5 seronegative and seropositive volunteers (Group 3, companion paper Tamminga et al). Here we report the results of Group 1 (low dose) and Group 2 (high dose) showing that NMRC-M3V-Ad-PfCA was safe and well tolerated, and induced strong, primarily CD8+ T cell interferon-gamma (IFN-γ) responses. The results indicate a potential trade-off between cell-mediated and antibody mediated immunity, the former best induced by the lower dose of the vaccine, the latter by the higher dose.

## Methods

The protocol for this trial and supporting CONSORT checklist are available as supporting information; see Checklist S1 and Protocol S1.

### Ethics

The study protocol for the clinical trial presented in this manuscript was approved by the National Naval Medical Center, Naval Medical Research Center and Walter Reed Army Institute of Research Institutional Review Boards, in compliance with all applicable federal regulations governing the protection of human subjects. All study subjects gave written informed consent. This study was conducted according to all Federal Regulations regarding the protection of human participants in research including The Nuremberg Code, The Belmont Report, 32 CFR 219 (The Common Rule) and all regulations pertinent to the Department of Defense, the Department of the Navy, the Department of the Army, the Bureau of Medicine and Surgery of the United States Navy and the internal policies for human subject protections and the standards for the responsible conduct of research of the Naval Medical Research Center (NMRC) and US Army Medical Research and Materiel Command (USAMRMC). NMRC holds a Department of Defense/Department of the Navy Federal Wide Assurance for human subject protections, and a Federal Wide Assurance (FWA 00000152) from the Office for Human Research Protections (OHRP) for cooperation with the Department of Health and Human Services. All NMRC key personnel are certified as having completed mandatory Command human research ethics education curricula and training under the direction of the NMRC Office of Research Administration (ORA) and Human Subjects Protections Program (HSPP).

### Participants

Volunteers were healthy malaria-naïve civilian and military adult men and women, age 18–50 years. The study was conducted at the Naval Medical Research Center (NMRC) Clinical Trials Center in Bethesda, Maryland. The protocol was described to potential volunteers by an investigator. Volunteers were required to demonstrate adequate comprehension of the requirements and risks of participation in the study by passing a written Assessment of Understanding. Informed consent was obtained and volunteers were screened for participation using inclusion and exclusion criteria detailed in the clinical protocol (supplementary material), which included good health and no significant prior exposure to malaria.

Groups 1 and 2 required the enrolment of 12 healthy adult volunteers, six in each group, all of whom were classified as seronegative when tested for neutralizing antibodies to adenovirus serotype 5. Volunteers were considered seronegative if prescreening titers were ≤1/500 using an adenovirus 5 neutralization assay that is monitored by luciferase reporter gene expression (NVITAL, Bethesda, MD) [37]. Volunteers with previous history of exposure to malaria or prior participation in malaria vaccine trials were excluded.

### Interventions

**Figure 1:**
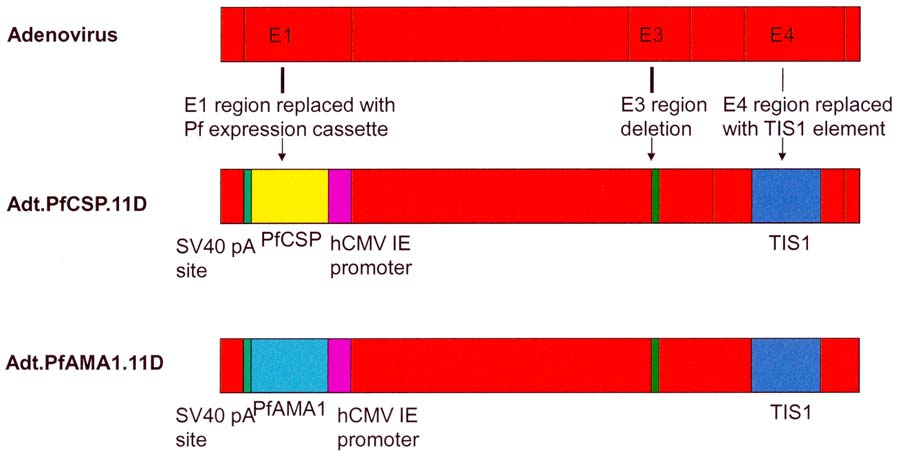
Schematic of Ad5-PfCSP and Ad5-PfAMA1 vectors. The parent adenovector was a serotype 5 adenovirus carrying deletions in E1, E4 and part of the E3 region with a transcriptionally inert spacer inserted into the site of the E4 deletion (TIS1), the resulting replication defective vector called GV11D. Codon-optimized CSP or AMA1 genes were inserted into the E1 region under the control of a cytomegalovirus promoter (hCMV IE). SV40 pA = simian virus 40 polyadenylation sequence.

NMRC-M3V-Ad-PfCA (NMRC - Multi-antigen Multi-stage, Malaria Vaccine - Adenovectored - *P. falciparum* CSP & AMA1 antigens) is a combination of two separate recombinant Ad5 constructs expressing the 3D7 strain of *P. falciparum* CSP (NMRC-MV-Ad-PfC) or AMA1 (NMRC-MV-Ad-PfA). The vector is derived from GV11D (GenVec, Inc.) and is missing the E1 and E4 regions required for replication, as well as part of the E3 region (Figure 1). The CSP and AMA1 genes were codon-optimized for expression in human cells and were inserted in the E1 region under the transcriptional control of a modified human cytomegalovirus promoter. The PfAMA1 gene is identical to native PfAMA1. The PfCSP gene was altered by the deletion of 16 of the 38 native NANP repeats (64 amino acids), and insertion of 23 amino acids (derived from the 3′-noncoding bovine growth hormone polyadenylation sequence) at the C-terminus. Although the glycosylphosphatidylinositol (GPI) anchor residues at the C-terminus have reduced immunogenicity in other studies [38], the GPI anchor contains T-epitopes that could potentially contribute to protection and thus was retained.

The vectors were constructed using GenVec's AdFAST technology using a homologous recombination system in *E. coli* to generate new adenovirus recombinants that were amplified in the 293-ORF6 cell line, which functionally complements the essential E1 and E4 deletions in the Ad5 vector [39]. Expression of the PfCSP and PfAMA1 proteins was confirmed by Western blot analyses using the PfCSP-specific NFS1 and the PfAMA1-specific 4G2 (dc1) monoclonal antibodies. The integrity of the vectors was confirmed by PCR, restriction enzyme digest and DNA sequence analyses of each strand. The vectors were manufactured in the 293-ORF6 cell line, under serum-free suspension culture by GenVec, Inc (Gaithersburg, MD). The supporting 293ORF6 cell line provides the E1 and E4 proteins in trans [39]. Production lots passed purity, identity and sterility tests. Each construct was vialed separately at 1×10^11^ particle units (pu)/mL in Final Formulation Buffer (FFB) (total volume 0.6 mL/vial). Each lot of vaccine and FFB remained within specifications for the duration of the clinical trial. The constructs were vialed separately in final formulation buffer (FFB) and equal proportions were mixed, diluted to a final concentration with FBB as needed and then loaded into a single syringe prior to injection (1 mL injected in total).

Since this was the first human study of these vaccines, a staggered schedule of immunizations was used and the safety and tolerability results from this study were reviewed by a Safety Monitoring Committee and a written report was submitted to the FDA prior to proceeding to the study of NMRC-MV-Ad-PfC administered alone in two doses described in the companion paper Tamminga et al. The schedule and doses of Trials 1 and 2 are shown in Table 1.

**Table 1:** Vaccine constructs, doses, and times of immunization.

Vaccine	Trial	Group (n)	Particle units (pu) per dose			# doses	Total dose (pu)
			CSP	AMA1	Total		
NMRC-M3V-Ad-PfCA(CSP & AMA1 mixed)	1	1 (6)	1×10^10^	1×10^10^	2×10^10^	1	2×10^10^
		2 (6)	5×10^10^	5×10^10^	1×10^11^	1	1×10^11^
NMRC-MV-Ad-PfC(CSP only)	2	3 (15)	1×10^10^	0	1×10^10^	2	2×10^10^

**Trial 1**: Dose escalation study: Group 1 was immunized with a single dose, a safety review was conducted, then Group 2 was immunized with a single five-fold higher dose.

**Trial 2**: Challenge study. Group 3 was immunized twice using the Group 1 dose of CSP, with 16 weeks between doses. Fifteen volunteers received the first dose, 14 volunteers the second, and 12 volunteers underwent malaria challenge 21 days following the second immunization. Data from Trial 2 are presented in the companion paper, Tamminga et al.

Lower dose Group 1 (n = 6): 0.5 mL from each vial (containing the CSP or the AMA1 construct) were mixed with 4 mL of FFB in an empty vial giving a final volume of 5 mL at 2×10^10^ pu/mL (1×10^10^ pu/mL of each construct). Six volunteers received 2×10^10^ pu of NMRC-M3V-Ad-PfCA as a single dose into the deltoid muscle of the non-dominant arm by needle injection.

Higher dose Group 2 (n = 6, eight months later): 0.55 mL from each vial were mixed in an empty vial without the addition of FFB and one mL was withdrawn for intramuscular injection (1×10^11^ pu, or 5×10^10^ pu of each construct, a five-fold dose escalation).

In this dose escalation trial, six malaria-naïve volunteers lacking evidence of prior exposure to human Ad5 (as determined by adenovirus neutralizing assay) received one intramuscular (IM) injection into the deltoid muscle of 2×10^10^ particle units (pu) of the combination vaccine (Group 1) and approximately one year later, after demonstration of safety, six additional volunteers received a single five-fold higher dose of 1×10^11^ pu (Group 2). A single administration was selected because it has been noted that a single dose is often more effective than two doses [40].

### Objectives

The primary objective was to assess the safety and tolerability of low and high doses of NMRC-M3V-Ad-PfCA vaccine. Individuals seronegative for neutralizing antibodies to Ad5 (NAb) were selected to reveal the full reactogenicity of the vaccine as others had reported that Ad5-specific NAb lowered the frequency of adverse events (AEs) [41]. The second objective was to measure the immunogenicity of two different doses of the vaccine in Group 1 (lower dose) and Group 2 (higher dose).

### Outcomes

To evaluate safety, tolerability and reactogenicity, AEs were recorded after each immunization. Solicited local and systemic events were recorded during a 14 day follow-up period while unsolicited events were collected over a 28 day follow-up period. Serious AEs were collected throughout the duration of the study for each group. Safety assessments were recorded by telephone interview (day 1), direct observation and questioning (days 2, 7, 10, 14 and 28) and by a diary card (days 0 to 7, for self-recording of oral temperature and symptoms). Safety laboratory assays, including complete blood count and a chemistry panel, were performed on samples collected at the in-person visits detailed above except during the day 10 follow up visit when only immunogenicity analysis was performed. In addition, a urinalysis was performed on day 14. All clinical laboratory tests were performed at the NNMC clinical laboratory. Only those laboratory abnormalities that were gradable by protocol or FDA/CBER guidelines are reported here. The in-person follow up period for Groups 1 and 2 was approximately 1 year. Local, systemic, and laboratory AEs were graded using severity scales detailed in the protocol (supplementary material). Telephone or email follow-up for all study subjects is to extend for a total of five years per FDA request.

### Sample size

The study was designed to demonstrate the vaccine's safety and tolerability profile in a small number of volunteers, thereby providing evidence that the frequency of serious or severe vaccine-related AEs was sufficiently low to continue testing in larger numbers of volunteers. If none of the twelve immunized volunteers experienced a serious or severe vaccine-related AE, the following predictions could be made regarding vaccine safety: there was a 46% level of confidence that the true rate of severe or serious vaccine-related AEs in the general population would be less than 5%; alternatively, there was a 72% level of confidence that the true rate of these events in the general population would be less than 10%; or, as a third example, there was a 93% level of confidence that the true rate would be less than 20%. These figures were determined using the exact binomial method (1-p)^n^ = 1-c where p is the probability that a subject has an event, n is the total number of subjects and c is the level of confidence.

### Immunological endpoints

Immunological assessments were performed pre-immunization; 10 days (d); and 1, 4, 7, 10 and approximately 12 months (m) post immunization.

#### Synthetic peptides and peptides pools

Peptides used for ELISpot assays were synthesized by Mimotopes, VIC, Australia (>80% purity).). The full length *P. falciparum* CSP sequence (GenBank no. X15363) was covered by a series of 15 amino acid (aa) peptide sequences over lapping by 11 aa. These were combined into nine pools (Cp1–Cp9) containing three to 12 peptides, such that most pools contained previously characterized T cell epitopes [42], [43], [44], [45] (Table 2). Similarly, 15 aa peptides spanning the full length of *P. falciparum* AMA1 (Gene Bank ID 810891) were combined into 12 pools each containing 10–13 peptides (Ap1–Ap12) and 10 pools contained a previously identified T cell epitope [46] (Table 3).

**Table 2:** CSP peptides used in ELISpot and ICS assays.

Pool	CSP aa	# Pep*	Class	HLA restriction	Residues	Sequence
**1** +	1–39	7	I	A2.1 supertype	1–10	MMRKLAILSV
			I	A2 supertype	7–16	ILSVSSFLFV
			II	DR (A2.1 and A2 supertype)	1–20	MMRKLAILSVSSFLFVEALF
**2** +	29–71	8				
3	61–107	9	I	B8	81–89	KLRKPKHKK
4	97–283	12	II	DR	105–116	NANPNVDPNANP
5	273–319	9	II	DR (B7)	281–300	QGHNMPNDPNRNVDENANAN
			I	B7	86–94	MPNDPNRNV
**6** +	309–331	3	I	A1	310–319	EPSDKHIKEY
			I	A2.1	319–327	YLNKIQNSL
			II	DR (A2.1)	316–335	IKEYLNKIQNSLSTEWSPCS
7	321–335	6	II	Th2r	326–343	SLSTEWSPCSVTCGNGIQ
8	345–367	3	II	B35-Th3r	346–365	IKPGSANKPKDELDYANDIE
**9** +	357–397	8	II	DR	363–383	DIEKKICKMEKCSSVFNVVNS
			II	DR (A2 supertype)	375–397	SSVFNVVNSSIGLIMVLSFLFLN
			I	A2 supertype	386–394	GLIMVLSFL

PfCSP peptide sequences and residue numbers were based on those of the *P. falciparum* clone 3D7 (GenBank no. X15363). Previously identified Class I and II CSP epitopes were distributed among peptide pools, except pool 2.

*Number of 15mer peptides in each pool.

+ Peptide pools used in ICS assays.

**Table 3:** AMA1 peptides used in ELISpot and ICS assays.

Pool	AMA1 aa	# Pep*	Class	T epitope%	Residues	Sequence
**1** +	1–63	13	II	PL186	14–35	EFTYMIFNRGQNYWEHPYQKS
				PL187	41–51	INEHRPKEY
**2** +	53–115	13		PL188	92–103	NLFSSIEIVERS
**3** +	105–167	13				
**4** +	157–219	13		PL189	188–204	PLMSPMTLDEMRHFYKD
5	209–271	13		PL190	218–229	SRHAGNMIPDND
				PL191	259–271	NGPRYCNKDE
6	261–323	13		PL192	279–288	AKDISFQNYT
**7** +	313–375	13		PL171	348–366	DQPKQYEQHLTDYEKIKEG
**8** +	365–427	13		PL193	390–402	YKSHGKGYNWGNY
9	417–479	13		PL172	444–461	SLYKNEIMKEIERESKRI
**10** +	469–531	13				
**11** +	521–583	13		PL194	527–538	EYKDEYADIPEH
				PL173	571–588	GNAEKYDKMDEPQHYGKS @
12	573–622	10		PL173	571–588	GNAEKYDKMDEPQHYGKS @

AMA1 peptide sequences and residue numbers were based on those of the *P. falciparum* clone FC27. Gene Bank ID 810891. Previously identified Class II AMA1 epitopes were distributed among peptide pools, except pools 3 and 10.

*Number of 15mer peptides in each pool.

% As described in reference 46.

+ Peptide pools used in ICS assays.

@ Epitope PL173 overlaps AMA1 peptide pools 11 and 12.

#### Interferon-gamma Enzyme Linked Immunospot Assays (IFN-γ ELISpot Assays)

Antigen-specific circulating peripheral blood mononuclear cells (PBMC's) were evaluated by modification of previously described methods [47]. Briefly, freshly isolated or cryopreserved PBMC at 100 K, 200 K, or 400 K suspended in 100 µL complete medium were stimulated with CSP and AMA1 15-mer peptide pools suspended in 100 µL of complete medium at 10 µg/mL of each 15-mer peptide in the pool tested. The positive control was CEF-Class I Peptide Pool *Plus* (CTL, Ohio, USA) consisting of 32 peptides corresponding to defined HLA class I-restricted T-cell epitopes from cytomegalovirus, Epstein-Barr virus and influenza virus. Cultures were incubated for 36 hours (hr) at 37°C, 5% CO_2_. Each PBMC sample was assayed in triplicate or quadruplicate and the number of IFN-γ-secreting cells recognized as spot-forming cells (sfc) was counted using an automated ELISpot reader (AID, GmbH, Germany). For each triplicate or quadruplicate, outliers were rejected if any single value contributed more than 50% of the standard deviation of the triplicate (or quadruplicate) and if its value was three-fold greater or less than the average of the remaining two (or three) values. After removing outliers, the mean sfc per million PBMC (sfc/m) obtained in negative control wells was subtracted from the value of each test well. Negative counts generated by this background subtraction were converted to zero. The mean of the test sample was then calculated and expressed as sfc/m. For each sample tested, the response against CSP or AMA was presented as responses against each individual peptide pool as well as the summed sfc/m responses against all pools representing the antigen.

#### Criteria for positive ELISpot response

For each CSP and AMA1 peptide pool tested against any given bleed, a positive response was defined as (1) a statistically significant difference (p = <0.05) between average of the number of spot forming cells in triplicate or quadruplicate test wells and the average of triplicate or quadruplicate negative control wells (Student's two tailed *t*-test), plus (2) at least a doubling of spot forming cells in test wells relative to negative control wells, plus (3) a difference of at least ten spots between test and negative control wells. The volunteer was designated as a responder when positive against at least one of the pools tested at any post immunization sampling.

#### Characterization of IFN-γ-producing cells by cell depletion or enrichment studies

ELISpot assays were carried out with PBMCs after depletion of T cell subsets using anti-human CD4+- or anti-CD8+-coated Dynabeads M-450 (Dynal, Great Neck, NY) following the manufacturer's instructions. Mock depletion was done by using Dynabeads coated with sheep anti-mouse IgG. Flow cytometry confirmed that T-cell subset depletions were >99% in all experiments. The data is presented as the % change in activity after T cell subset depletion.

#### Intracellular cytokine staining (ICS)

For each volunteer, matched pre- and post-vaccination samples were tested simultaneously. ICS was performed under a similar protocol as published previously [48]. Briefly, vials of cryopreserved test cells were thawed, washed, and resuspended at 10×10^6^ cells/mL in 20% fetal calf serum (FCS) (Hyclone, Logan, UT) in complete RPMI (cRPMI) (RPMI-1640 (BioWhittaker, Walkersville, MD) supplemented with penicillin/streptomycin (Sigma, St. Louis, MO), 2-ME, non-essential amino acids, pyruvate, and glutamine (Gibco, Grand Island, NY). All stimulants were diluted in cRPMI with costimulatory antibodies anti-CD28 and anti-CD4+9d (BD Bioscience, San Jose, CA) at a final concentration of 1 µg/mL each (referred to as M+). Due to cell availability, only subsets of the CSP (C1, C2, C6, and C9) and AMA1 (A1, A2, A3, A4, A7, A8, A10, and A11) peptide pools were tested at 10 µg/mL for each peptide. CEF peptide pool (Anaspec, San Jose, CA) was used at 2 µg/mL each peptide as an antigen-specific CD8+ positive control for each volunteer. A bridging volunteer whose responses were well characterized in our lab was included in each assay and responses to M+, CEF and Staphylococcal enterotoxin B (SEB) (Sigma; 0.5 µg/mL) were monitored to be within the normal range. Stimulants were added to cells and incubated at 37°C with 5%CO_2_ for 2 hr. Golgi Plug (Brefeldin A) (BD Bioscience) was added at a final concentration of 0.6 µL/mL and incubated at 37°C with 5%CO_2_ overnight, approximately 13–15 hr. Cells were stained with anti-CD3 Alexa Fluor 700, anti-CD4+ PerCP, and anti-CD8+ Pacific Blue (all BD Bioscience) and 1 µg/mL of live/dead fixable blue dye (Invitrogen), incubated and washed. Cells were permeabilized with Cytofix/Cytoperm solution (BD Bioscience), incubated and washed. Cells were stained intracellularly with anti-CD3 AlexaFluor700, anti-CD4+ PerCP, anti-CD8+ Pacific Blue, anti-IFNFITC, anti-TNF PE, and anti-IL-2 APC, incubated and washed. Cells were resuspended and acquired on a BD LSRII using FACSDiVa (BD Bioscience) software.

7-color flow cytometry was used to investigate the phenotype of responding cells, and dead or dying cells were excluded from analysis. Cells were phenotyped as CD4+ and CD8+ T cells and assessed for functionality (cytokine secretion) by staining for IFN-γ, TNF-α and IL-2. The gating strategy involved progressively measuring total cells; viable cells only; lymphocytes; T cells; CD4+ CD8+ populations; and finally a specific cell type expressing a specific cytokine (Figure S1). Histograms were used to determine the total production of IFN-γ, IL-2, and TNF-α for the CD4+ or CD8+ populations (a total of 6 histograms). Boolean gates were used to determine cells producing combinations of cytokines. Results were tabulated and transferred to Microsoft Excel and Prism (GraphPad) for graphing and statistical analysis. Data for peptide pools were corrected for media response at each time point.

All pre- and post-vaccination PBMC samples for a given volunteer were run in a single assay. The total IFN-γ responses (T-IFN-γ) for the CD4+ and CD8+ T cell subsets were measured regardless of which other cytokines were expressed. The T-IFN-γ included IFN-γ+ IL-2+ TNFα+, IFN-γ+ IL-2+ TNFα−, IFN-γ+ IL-2− TNFα+ and IFN-γ+ IL-2− TNFα-containing cells. Multifunctional responses were calculated as cells that produced 2 cytokines (IFN-γ + IL-2+ TNFα−, IFN-γ+ IL-2− TNFα+, IFN-γ−IL-2+ TNFα+) or all 3 cytokines (IFN-γ + IL-2+ TNFα+). Finally, we examined the relative proportion of cells producing any 1, 2 or 3 cytokines, where cells secreting just TNFα or IL-2 were included in the singles category. For time points after immunization, responses were corrected for pre-bleed responses so that magnitudes depicted are attributable to vaccination.

#### Enzyme-Linked Immunosorbent Assay (ELISA)

ELISA was used to measure total IgG antibody titers against the *P. falciparum* CSP central repeat region using a hexameric synthetic peptide (NANP)_6_ as the capture antigen and against *P. falciparum* AMA1 using recombinant ectodomain protein [49]. Briefly, plates were coated overnight at 4°C with (NANP)_6_ or AMA1 (100 µL/well, 0.5 µg/mL), after which they were blocked with 0.5% boiled casein buffer for 1 hr at 22°C. Test samples were added to the plate, diluted in eight sequential two-fold dilutions (done in triplicate) and incubated for 2 hr at 22°C. Secondary antibody (Affinity Purified Peroxidase Labeled Goat anti-Human IgG (γ), KPL, Gaithersburg, MD: Cat# 074-1002) at a 1∶4,000 dilution, was added and incubated 1 hr at 22°C. A stop solution (20% SDS) was added and the plates were read using a Spectromax 340OC Plate Reader (Molecular Devices, Sunnyvale, CA). Between each incubation step the wells were washed in PBS using a SkanWasher Plate Washer (Molecular Devices) with four washing cycles of 400 µL each. Titer was defined as the serum dilution required yielding an optical density reading of 1.0. Seroconversion was established when post-immunization antibody titers differed significantly (p<0.05, two tailed student t-test) from pre-immunization titers.

#### Immunofluorescent Antibody Assay (IFA)

*P. falciparum* sporozoite-specific antibodies were assayed by immunofluorescent staining of air-dried *P. falciparum* sporozoites and *P. falciparum* parasitized red blood cells as described previously [50].

#### Functional assay

*In vitro* growth inhibition of cultured *P. falciparum* strain 3D7 blood stages was performed at the GIA Reference Center at the National Institutes of Health by Dr. Carole Long [51].

### Statistical methods

SAS software was used to conduct all analyses [52] unless mentioned otherwise. A mixed model, ANOVA model III, was used to compare means of T cell responses (ELISpot, ICS) between experimental groups and antigens. The model consisted of group and time as fixed effect, a two-way interaction of group versus time as fixed effect, and the individual as random effect. Natural log transformation was used on the outcome measure to stabilize the variance. For flow cytometry, acquired data files were analyzed using FlowJo version 8.7.1 (Tree Star, Inc.), and SPICE 4.1.6 (developed by Mario Roederer, Vaccine Research Center, NIAID/NIH). One-way ANOVA with Dunnett's post-test was performed for comparisons between pre- and post-vaccination time points (as indicated), using GraphPad Prism version 5.00 for Windows (GraphPad Software, San Diego, CA, USA). For comparison of activities between groups at specific time points Mann Whitney U tests and two-tailed probabilities were used. To compare MFI activities of triple and single IFN-γ secreting CD4+ and CD8+ T cells for Group 1 volunteers, the average MFI for each volunteer using CSP and AMA1 peptides was log_10_-transformed, values entered into Mann-Whitney U tests and the two-tailed significance calculated.

## Results

### Participant flow

**Figure 2:**
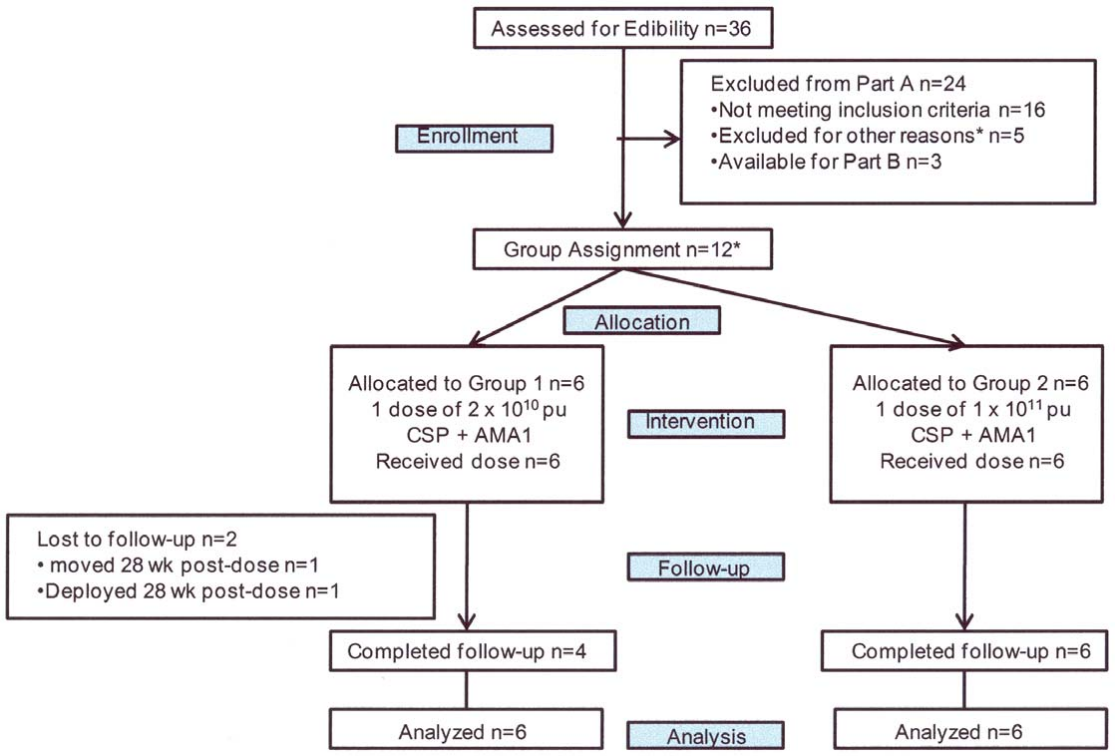
Flow diagram of volunteers in Groups 1 and 2. The first six volunteers were allocated to Group 1 and the subsequent six volunteers to Group 2. *Reasons for exclusion: lost to follow up (2), moved out of area (1), deployed (1), job commitments (1).

Participant flow is shown in Figure 2. Recruitment took place between October 2006 and January 2008. 36 healthy, malaria-naïve, civilian and military adult men and women, aged 18–50 years, were assessed for eligibility and 16 were excluded. A further five were excluded for other reasons, and three were assigned to the follow-on efficacy study (Tamminga et al). The remaining 12 volunteers who met all screening criteria were assigned sequentially into the low vaccine dose Group 1 (n = 6) and then the higher vaccine dose Group 2 (n = 6). Ten of the 12 immunized volunteers completed active follow up for one year. Two volunteers were lost to follow up after completing 28 weeks of participation following vaccine administration.

The demographic make-up of the participant volunteers is shown in Table 4. The vaccine groups were similar in age and ethnicity although Group 1 had more males (5) than Group 2 (2). All 12 immunized volunteers received their scheduled vaccinations and were analyzed for safety and immunogenicity.

**Table 4:** Demograpic characteristics of volunteers enrolled in Group 1 and Group 2.

	Group 1N = 6	Group 2N = 6
Male #	5	2
Female #	1	4
Age range 18–20 years	0	1
Age range 21–30 years	3	2
Age range 31–40 years	2	1
Age range 41–50 years	1	2
Overall age range years	22–43	19–49
Median age years	32.5	33.5
Mean age years	32.6	34.0
Caucasian #	3	3
African-American #	2	3
Hispanic #	1	0
Asian #	0	0
Individual Ad5 NAb titers	<12,<12,<12<12,34,359	<12,<12,<12,<12,19,48

The demographic distribution was similar in all categories, except there were more males than females in Group 1, and more females than males in Group 2.

### Local and systemic adverse events

Solicited post-immunization AEs recorded in each 14-day follow-up period are shown in Table 5. The most common local side effect was injection site pain and/or tenderness, occurring in five of six (83%) volunteers in both Group 1 (low dose) and Group 2 (high dose). Most of these events were mild in severity. Ipsilateral axillary lymphadenopathy with axillary tenderness occurred in two Group 1 volunteers following immunization, attributed to the effects of the vaccine. No volunteer in Group 2 developed lymphadenopathy although one volunteer did experience ipsilateral axillary tenderness accompanied by medial arm pain.(recorded as unsolicited AEs – see below). No Grade 3 local AEs occurred in either group.

**Table 5:** Local and systemic Adverse Events in Group 1 and Group 2.

Sign or Symptom	Group 1 N = 6 CSP+AMA1 low dose		Group 2 N = 6 CSP+AMA1 high dose	
	All	Gr 3	All	Gr 3
**LOCAL**				
Pain/Tenderness	5 (83)	0	5(83)	0
Erythema	0	0	0	0
Induration/Swelling	0	0	1(17)	0
Warmth	0	0	1(17)	0
Hives	0	0	0	0
Lymphadenopathy	2(33)*	0	0	0
Limited arm motion	1(17)*	0	0	0
**MEAN**† **LOCAL**	**1.3**		**1.2**	
**SYSTEMIC**				
Headache	2(33)	0	3(50)	1(17)
Malaise	1(17)	0	4(67)	0
Fever (objective)	0	0	2(33)‡	0
Fever (subjective)	1 (17)	0	2(33)	0
Chills	0	0	4(67)	1(17) §
Myalgia	2(33)	0	2(33)	1(17)
Arthralgia	1(17)	0	1(17)	0
Nausea/vomiting	2(33)	0	3(50)	0
Diarrhea	0	0	1(17)	0
Blurred vision	0	0	1(17)	0
Dizziness	0	0	1(17)	0
**MEAN**† **SYSTEMIC**	**1.5**		**4.0**	**0.5**

Gr = severity grade.

*ipsilateral axillary tenderness.

The most common systemic, vaccine-related AEs in Group 1 were headache, myalgia, and nausea, each occurring in two of six volunteers, mostly of mild severity. In Group 2, headache and nausea and/or vomiting each occurred in three of six volunteers, also mostly of mild severity, but Group 2 volunteers also developed malaise (four of six), subjective (two of six) or objective (two of six) fever and chills (four of six). Although most of these events were mild, malaise was more commonly of moderate severity.

One volunteer in Group 2 (v23) reported an episode of severe chills, myalgia, and headache along with milder dizziness and malaise; she reported that this was followed by a sensation of difficulty talking and swallowing, throat swelling, blue discoloration to her fingernails and nausea (with these latter complaints all resolving that evening). The onset of symptoms was approximately 8 hours post vaccination while the volunteer was at work; these events were not observed by the study team. Pulse oximetry taken during the episode at the request of the volunteer, by a nurse at the volunteer's place of employment, was reportedly normal. Because of the timing, this cluster of symptoms was listed as probably related to vaccination. This volunteer was one of two in Group 2 who experienced an objective fever with a temperature of 100.6°F (Grade 1) on day 1 post vaccination. In addition, this volunteer also experienced a Grade 3 decrease in absolute neutrophil count and a Grade 1 decrease in WBC on day 2; both of these laboratory AEs had normalized by day 8 (see below for laboratory AEs). One vaccine-unrelated serious adverse event (SAE), hospitalization for depression and alcohol use, occurred in a volunteer in Group 1 approximately 3 m after immunization. The volunteer had a prior history of these conditions.

Overall, the number of systemic but not local AEs related to the vaccine was significantly greater in Group 2 than in Group 1 when these two groups were compared (systemic: 24 in Group 2 vs. 9 in Group 1, p = 0.014) by Wilcoxon two-sample test, (and see analysis in Tamminga et al when the five most common or important AE's are considered). Subjects who reported more local AE's also reported more systemic AE's (data not shown).

### Unsolicited adverse events

Volunteers were questioned for unsolicited symptoms for 28 days following vaccine administration. Their unsolicited AEs are listed in Table S1. The unsolicited AEs considered definitely or probably related to immunization were: ipsilateral arm stiffness (n = 1) or pain (n = 1), axillary tenderness (n = 2, mentioned above, associated with axillary adenopathy in two volunteers), and the complex of symptoms experienced by v23, discussed above under “Local and Systemic Adverse Events”. Several volunteers experienced clinical syndromes of mild or moderate severity consistent with adenovirus infection, but the timing relative to immunization was variable suggesting that these were probably not related to immunization. The rate of occurrence of these miscellaneous clinical syndromes was consistent with background infections circulating in the community during the periods of immunization.

### Laboratory adverse events

**Figure 3:**
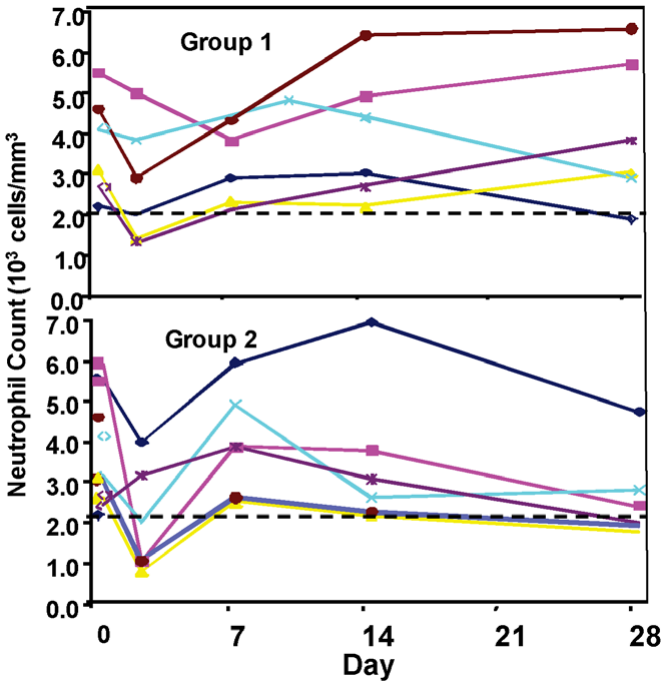
Neutrophil kinetics in Groups 1 and 2 after immunization. A fall in neutrophil count was observed in most volunteers at 2–3 days post-immunization with return to normal or near normal levels by day 7 post-immunization.

Safety labs were collected on days 0, 2, 7, 14, and 28 post immunization (complete blood count with differential; chemistry panel including sodium, potassium, blood urea nitrogen, creatinine, aspartate aminotransferase, alanine aminotransferase, phosphorous, glucose, calcium, albumin, total protein, bilirubin, alkaline phosphatase; and, on day 14 only, urinalysis including urine protein, glucose and blood). There was a fall in total white blood cell count (WBC) recorded in most volunteers on day 2 following immunization, with complete or near complete recovery by day 7. When examined by WBC subset, this consisted primarily of reductions in neutrophils (Figure 3) and to lesser degree lymphocytes. There was suggestive evidence of a dose-response, as neutropenia in Group 1 reached Grade 1 in one of six and Grade 2 in two of six volunteers, while in Group 2 it reached Grade 2 in two of six and Grade 3 in one of six volunteers. Only one volunteer (Group 2) had a neutrophil count less than 1000 cells per mm^3^ (936 cells) on day 2 post immunization, with resolution by day 8. There were no other laboratory trends noted.

### Immunogenicity

#### T cell responses to CSP by *ex vivo* IFNγ ELISpot Assay

**Figure 4:**
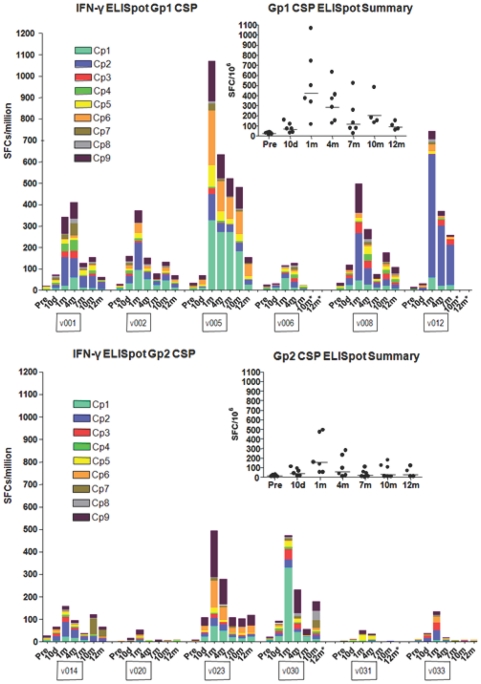
ELISpot activity of serial bleeds of volunteers in Group 1 and Group 2 with CSP peptide pools. The ELISpot activities of each volunteer at pre-immunization, 10 d and 1, 4, 7, 10 and 11–12 m are displayed using color-coded CSP peptide pools Cp1–Cp9. The inserts show the values of the sum of each volunteer's responses at each time point and the bar indicates the geometric mean of the group. At 1 month, geomean values were higher (422 sfc/m PBMC's) in the low dose Group 1 than the high dose Group 2 (154 sfc/m PBMC's).

**Figure 5:**
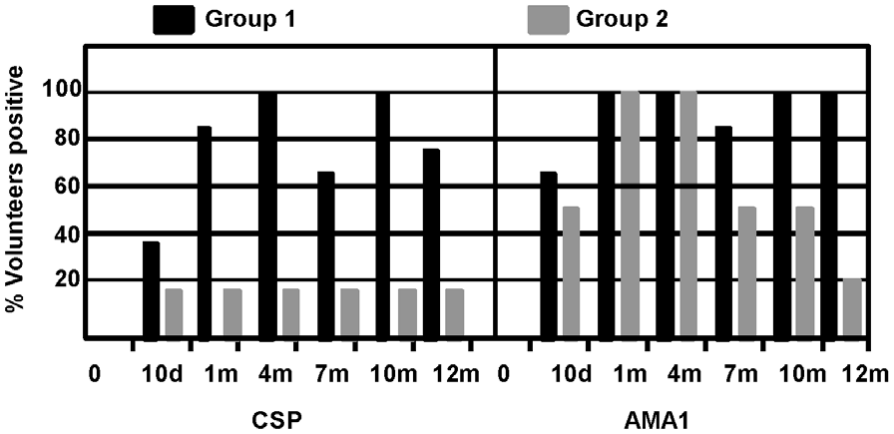
Percent of Group 1 and Group 2 volunteers who responded to one or more CSP or AMA1 peptide pools at each time point after immunization. ELISpot activity was determined to be positive or negative for a given peptide pool as described in Methods. A positive volunteer is one who recognized one or more peptide pools at each of the time points 10 d and 1, 4, 7, 10 and 11–12 m after immunization. The percent positive responders are displayed at each time point for CSP or AMA1.

ELISpot CSP IFN-γ responses of the six Group 1 volunteers (receiving the lower dose of vaccine, 1×10^10^ pu of each construct totaling 2×10^10^ pu) were detected at 10 d post immunization, peaked at 1 m (geometric mean response, summed across peptide pools for each volunteer, 422, range 114–1066 sfc/m), declined at 4 m, but were detected as late as 12 m (Figure 4, upper panel). Although responses differed among volunteers, all volunteers met criteria for positive responses at 4 m, not all were positive at 7 m but all became positive again at 10 m (Figure 5, left panel). ELISpot CSP IFN-γ responses of Group 2 (5×10^10^ pu each construct totaling 1×10^11^ pu) were qualitatively similar to those of Group 1 (Figure 4, lower panel), but were significantly lower at 1 m (geometric mean 154, range 52–493 sfc/m) and during the follow up period (p = 0.049, Anova). This finding was reflected in the fewer numbers of volunteers meeting criteria for a positive response in Group 2 compared to Group 1 (p = 0.045, Anova) (Figure 5, left panel).

To assess which peptide pools elicited the strongest responses, we selected those contributing at least 33% of the sfc/m recorded for the highest pool at a given time point. This analysis showed that Cp1, Cp2, Cp6 and Cp9 were the most strongly recognized at 1 m through 10 m (Table 6, upper panel, seen also in Figure 4, upper panel), while in Group 2, the same four pools were dominant at 1 m but not at later time points (Table 6, upper panel, seen also in Figure 4, lower panel).

**Table 6:** Immunodominant CSP and AMA1 peptide pools by ELISpot assay.

	Group 1						Group 2					
Cp	10 d	28 d	4 m	7 m	10 m	12 m	10 d	28 d	4 m	7 m	10 m	12 m
1		1	3	3	1			2	2		1	
2		6	5	3	2			2	2		1	
3												
4												
5												
6		2	1	1	1	1		1				
7												
8												
9		3	3	1	4			1	1			

Ap	10 d	28 d	4 m	7 m	10 m	12 m	10 d	28 d	4 m	7 m	10 m	12 m
1		2	2	2	2	1		1				
2		1	1		1							
3		3	2	1				2	1	2	1	1
4		3	4	2	1	1		2				
5		1	3									
6		1	3									
7		2	5		1							
8		4	4	1	1							
9			2		1			4	2	1	1	1
10		3	3	2	2	1		2	1			
11		1	3	1	1			1	1			
12		1	1									

The peptide pools that were most strongly recognized at each time point by a volunteer demonstrating an overall positive response were arbitrarily identified as immunodominant if the response to that pool exceeded one-third the value of the responses to the pool with the peak response in that volunteer. The number of volunteers who met criteria for immunodominance for a given peptide pool is shown in each box. Responses to CSP pools were consistent in both Groups but more variable to AMA1 in Group 1 and Group 2.

#### T cell responses to AMA1 by *ex vivo* IFN-γ ELISpot Assay

**Figure 6:**
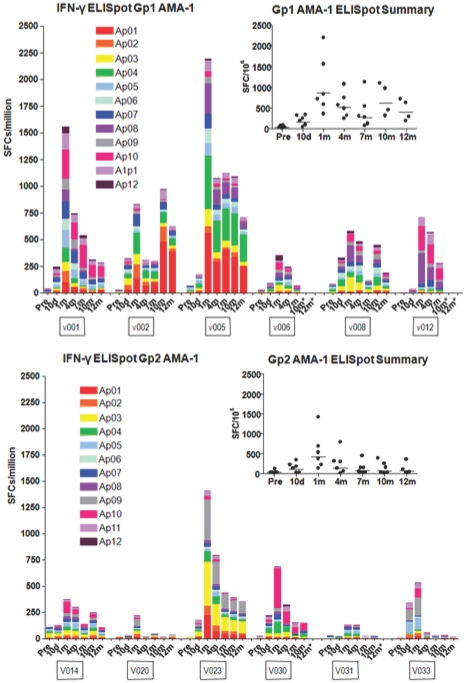
ELISpot activity of serial bleeds of volunteers in Group 1 and Group 2 with AMA1 peptide pools. The ELISpot activities of each volunteer at pre-immunization, 10 d and 1, 4, 7, 10 and 11–12 m are displayed using color-coded AMA1 peptide pools Ap1–Ap12. The inserts show the values of the sum of each volunteer's responses at each time point and the bar indicates the geometric mean of the group. At 1 month, geomean values were higher (862 sfc/m PBMC's) in the low dose Group 1 than the high dose Group 2 (422 sfc/m PBMC's).

As with CSP, low dose (Group 1) IFN-γ ELISpot responses to AMA1 were detected at 10 d, peaked at 1 m (geometric mean response, summed across peptide pools for each volunteer, of 862, range 353–2193 sfc/m) and declined at 7 m but were still detected in all volunteers at 10 and 12 m (Figure 6, upper panel), and were approximately two fold higher but with a similar kinetic response compared to CSP. As with CSP, IFN-γ ELISpot responses to AMA1 over the course of the study were significantly lower in the high dose group at 1 m (geometric mean of 423, range 135–1418 sfc/m) and for the duration of the study (Figure 6, lower panel, p = 0.045, Anova) and demonstrated more positive responders to AMA1 than Group 2 (p = 0.013, Anova) (Figure 5, right panel).

In Group 1 for AMA1, 11 out of 12 individual peptide pools were above the 33% cut off at 1 m, with Ap1, Ap4, Ap8 and Ap10 showing the strongest responses (Figure 5, right panel, seen also in Figure 6, upper panel) and remaining dominant at 4, 7, and 10 m. Ap11 also became dominant at 7 and 10 m, and Ap2 and Ap7 at 10 m. In contrast, in Group 2, 6 of the 11 peptide pools were above the cut off at 1 m and the strongest recognition was of Ap3 and Ap9, although Ap1, Ap4 and Ap10 as in Group 1 were also dominant at 1 m and 4 m (Table 6, lower panel, see also Figure 6, lower panel).

#### ELISpot CD4+ and CD8+ T cell depletion experiments

The role of CD4+ and CD8+ T cells in ELISpot responses was investigated by depletion of CD4+ or CD8+ T cells using frozen PMBC's from the low dose group (36 hr stimulation assay as for fresh ELISpot) (Table 7). After depletion, cell numbers were not further adjusted, and were tested for IFN-γ secretion. Due to limited availability of cells, only the four dominant CSP and ten dominant AMA1 peptide pools were tested. v006 was excluded, based on relatively low frequency of recognition in ELISpot assays. Generally, responses by each volunteer to peptide pools were reduced either by depleting CD4+ T cells only, or by depleting either CD4+ or CD8+ T cells. Only three CD8+ T cell-specific activities were observed, one with v001 and Cp2, one with v008 and Ap3, and one with v012 and Cp1. With v001, ELISpot activity in response to five peptide pools rose after CD8+ T cell depletion, suggesting that a subset of CD8+ T cells might have suppressed total ELISpot activity. In general, different outcomes for the various peptide pools were observed with each volunteer. For example Cp2 was recognized as containing a CD8+ T cell epitope by v001, a CD4+ T cell epitope by v002 and v005, and both CD4+ and CD8+ T cell epitopes by v008 and v012. It is likely that epitope mapping studies using these volunteers would identify multiple class I and class II-restricted epitopes in this region of CSP.

**Table 7:** Percent change in ELISpot responses after depleting CD4+ or CD8+ T cells at 1 m time-point.

Peptide Pool	V001		V002		V005		V008		V012	
Depleted Cells:	CD4+	CD8+	CD4+	CD8+	CD4+	CD8+	CD4+	CD8+	CD4+	CD8+
Cp1	*	*	*	*	*	*	*	*	NC	−57
Cp2	NC	−88	−90	NC	−100	NC	−77	−88	−55	−77
Cp6	*	*	−68	−72	−100	NC	*	*	−76	NC
Cp9	−100	+300	−74	NC	−99	NC	−61	−93	−100	NC
Ap1	*	*	−51	−100	−56	−99	*	*	*	*
Ap2	*	*	−92	NC	−100	−45	−80	−69	*	*
Ap3	*	*	−87	NC	−93	−34	NC	−90	*	*
Ap4	−91	+283	−62	−80	−57	−67	*	*	*	*
Ap5	−79	+145	*	*	*	*	*	*	*	*
Ap6	−65	+148	*	*	*	*	−90	NC	*	*
Ap7	NC	NC	−90	−61	*	*	−97	NC	−84	−65
Ap8	*	*	*	*	*	*	−67	−95	−57	−33
Ap10	−47	−66	*	*	*	*	*	*	−77	−92
Ap11	−76	+78	−52	−75	−80	NC	*	*	−83	−100

IFN-γ production by ELISpot assay was measured with and without depletion of either CD4+ or CD8+ T cell populations and is expressed as percent change in spot forming cells with depletion. A negative value indicated IFN-γ decreased by the indicated percentage after depletion, and a positive value indicated IFN-γ increased after depletion. All peptide pools tested in ELISpot depletion studies met criteria for positive in the absence of depletion (Figures 1 and 2). Any decrease or increase in ELISpot responses of ≥25% following depletion was scored as positive and a number is provided in the table, while changes <25% were scored as no change (NC).

*Not tested.

#### Total-IFN-γ (T- IFN-γ) responses to CSP peptides by ICS

**Figure 7:**
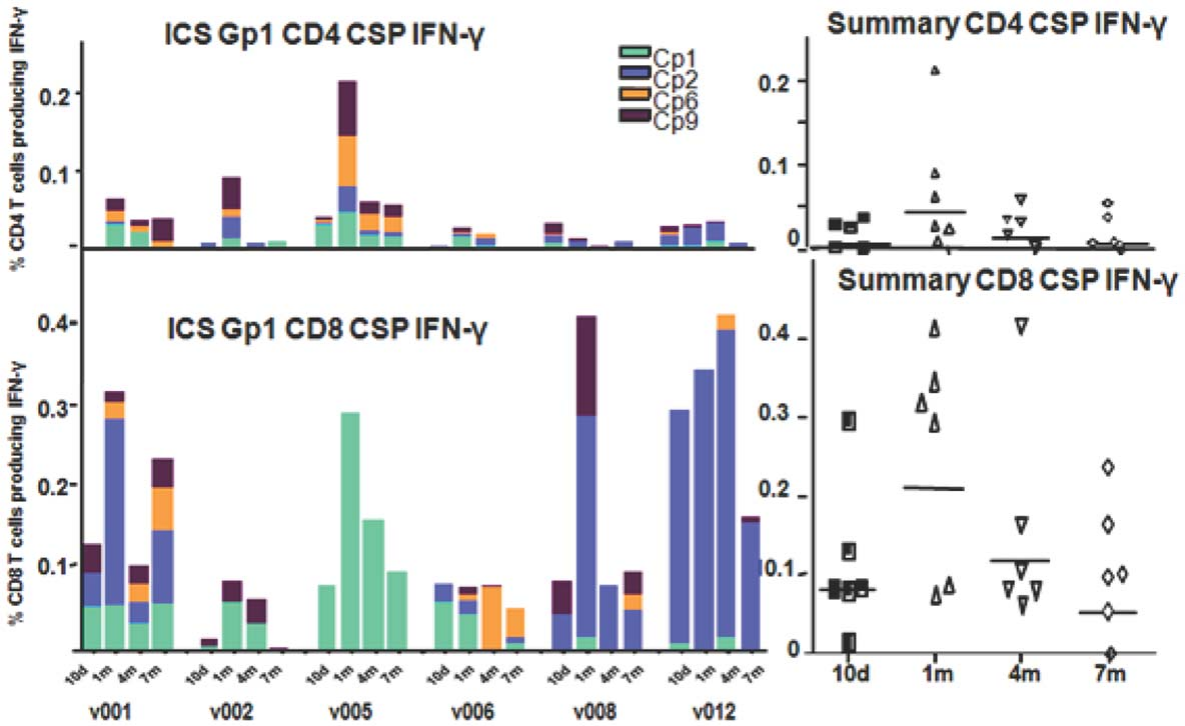
T-ICS CD4+ and CD8+ IFN-γ activity of serial bleeds of volunteers in Group 1 with CSP peptide pools. Four Cp peptide pools that were most strongly recognized in ELISpot assays were used to determine ICS CD4+ and CD8+ IFN-γ activity. The ICS CD4+ and CD8+ T cell activities of each volunteer at 10 d and 1, 4, and 7 m (subtracting pre-immunization values) are displayed using color-coded CSP peptide pools. Scales for each phenotype have been equalized to emphasize the lower CD4+ responses. The inserts show the values of the sum of each volunteer's responses at each time point and the bar indicates the geometric mean of the group. At 1 month CD8+ responses (geomean 0.21% CD8+ T cells, std dev 0.14) were higher than CD4+ responses (geomean 0.044% CD4+ T cells, std dev 0.075).

Using frozen PBMC's, multi-parameter flow cytometry and ICS were conducted at time points following immunization of Groups 1 and 2, using the four most strongly stimulating CSP peptide pools in ELISpot assays (Cp1, Cp2, Cp6 and Cp9, Table 6). We first established that the mean ratio of CD4+ ∶ CD8+ T cells in the gated CD3+ T cell population of each volunteer was 2.3 (range 1.9–2.5, data not shown). Then we compared the T-IFN-γ producing CD4+ or CD8+ T cells in each group that included cells producing IFNγ only and cells that produced IFNγ in combination with one or two other cytokines (IL2, TNF-α) but not cells producing only IL2, TNF-α or their combination. In the low dose group (Group 1), total CD8+ IFN-γ responses to CSP generally peaked 1 m after immunization and were approximately 5 fold higher than total CD4+ IFN-γ responses as a percent of gated CD8+ or CD4+ T cells, respectively (geomeans 0.21% CD8+ [range 0.0761–0.4131%], 0.044% CD4+ [range 0.002–0.2125%]) (Figure 7). This difference between CD8 and CD4 was highly significant comparing all time points (10 d to 7 m) (p = 0.0001, Anova). When corrected for the 2.3 times larger number of CD4+ vs. CD8+ T cells in the gated CD3+ T cell population, the predominance of CD8+ over CD4+ T was approximately 2-fold.

The high dose group (Group 2) demonstrated a similar pattern of total CD8+ and CD4+ T cell IFN-γ responses to CSP by ICS as the low dose Group 1 (Figure S2) but with lower magnitude compared with Group 1, (geomeans 0.020% CD8+ [range 0.0003–0.3001%], 0.006% CD4+ [range 0.0013–0.0455%]) consistent with the lower magnitude observed with ELISpot responses (p = 0.0001 for CD8+ T cells, p = 0.054 for CD4+ T cells, Anova). CD8+ T cells predominated over CD4+ T cells as with Group 1 but this difference did not reach statistical significance (p = 0.13, Anova).

#### Total IFN-γ responses to AMA1 peptides by ICS

**Figure 8:**
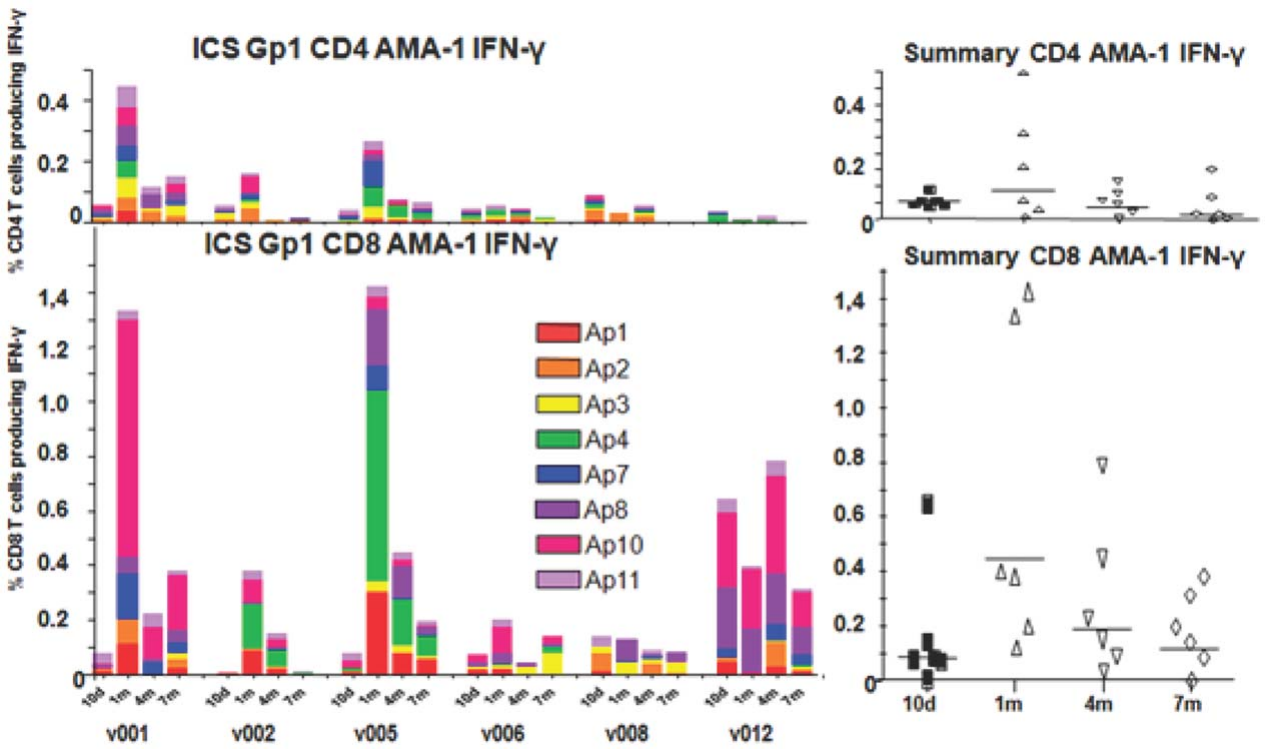
T-ICS CD4+ and CD8+ IFNactivity of serial bleeds of each volunteer in Group 1 with AMA1 peptide pools. Eight Ap peptide pools that were most strongly recognized in ELISpot assays were used to determine ICS CD4+ and CD8+ IFN-γ activity. The ICS CD4+ and CD8+ T cell activities of each volunteer at 10 days and 1, 4, and 7 months (subtracting pre-immunization values) are displayed using color-coded AMA1 peptide pools. Scales for each phenotype have been equalized to emphasize the lower CD4+ responses. The inserts show the values of the sum of each volunteer's responses at each time point and the bar indicates the geometric mean of the group. At 1 month, CD8+ responses (geomean 0.44% CD8+ T cells, std dev 0.58) were higher than CD4+ T-INF-γ responses (geomean 0.086% CD4+ T cells, std dev 0.17).

Multi-parameter flow cytometry and ICS were conducted using the eight strongest stimulating AMA1 peptide pools (Ap1, Ap2, Ap3, Ap4, Ap7, Ap8, Ap10, Ap11, Table 6) at the same time points as CSP following immunization of Groups 1 and 2. As with CSP, low dose (Group 1) total CD8+ T cell IFN-γ responses to AMA1 generally peaked at 1 m, were 5-fold higher than CD4+ T cell IFN-γ responses as a percent of gated CD8+ or CD4+ T cells, respectively (geomeans 0.44% CD8+ [range 0.1315–1.3302%], 0.086% CD4+ [range 0.0126–0.4480%]) (Figure 8) and remained so throughout the study (p = 0.003, Anova). This difference was also approximately 2-fold after correcting for the larger number of CD4+ compared with CD8+ T cells in the gated CD3+ T cell population. As with CSP, Group 2 AMA1 ICS total CD4+ IFN-γ and total CD8+ IFN-γ responses at 1 m (geometric means: 0.15% CD8+ [range 0.0122–1.0671%], 0.035% CD4+ [range 0.0046–0.1054%]) were lower than Group 1 (p = 0.008 for CD8+ T cells, p = 0.014 (Anova) for CD4+ T cells), but unlike CSP, the predominance of Group 2 CD8+ AMA1 T cell responses over CD4+ just reached significance (p = 0.0485, Anova) (Figure S3).

#### Multifunctional T cell responses to CSP and AMA1

We next evaluated multifunctional T cells that produced two or more cytokines: IFN-γ+ IL2+ TNF-α +; IFN-γ+ IL-2+ TNF-α−; IFN-γ + IL-2− TNF-α +; IFN-γ− IL-2+ TNF-α+.

**Figure 9:**
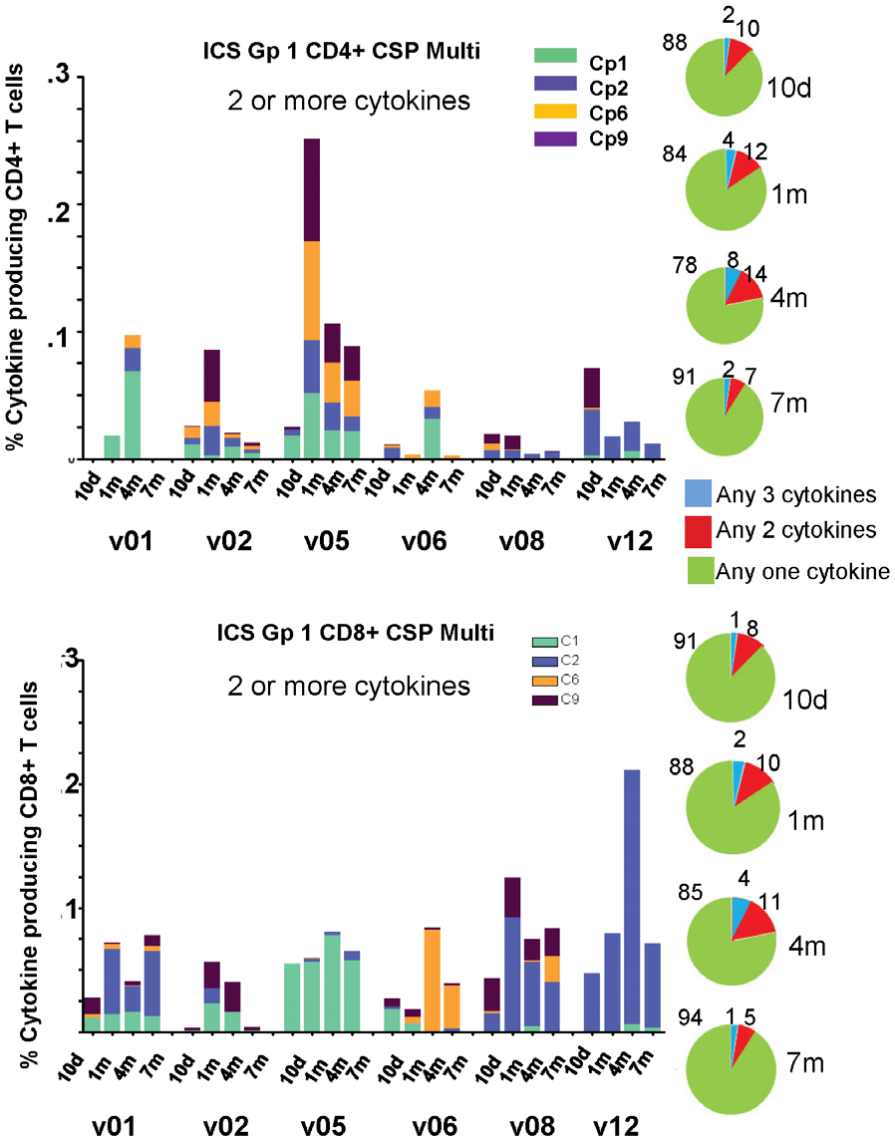
Multifunctional CD4+ and CD8+ T cells of serial bleeds of volunteers in Group 1 with CSP peptide pools. **A** and **C**: Multifunctional (any two cytokines) CD4+ and CD8+ T cell activities of each volunteer at 10 d, 1, 4, and 7 m after immunization (subtracting pre-immunization values) are displayed using color-coded CSP peptide pools. At 1 month, CD4+ multi responses (geomean 0.027% CD4+ T cells, std dev 953) and CD8+ multi responses (geomean 0.06% CD8+ T cells, std dev 346) were similar. **B** and **D**: Pie charts representing the proportion of the cytokine response indicated by cytokine subsets as shown; numbers on pie charts represent percent of that subset of the total cells.

Group 1 multifunctional T cell responses to CSP: the frequencies of multifunctional CD4+ and CD8+ T cell populations were similar as a percent of gated CD8+ or CD4+ T cells, respectively, at 1 m (geomean 0.06% CD8+ [range 0.0181–0.1244%], 0.027% CD4+ [range 0.0035–0.2507%]) (Figure 9, panels A and C), although CD8+ multifunctional cell frequencies were more sustained over the 7 m period tested than CD4+ multifunctional cells (p = 0.0016, Anova) (Figure 9). The frequencies at 1 m represented an increase in the proportion of multifunctional cells relative to 10 d for both CD8+ and CD4+ populations (Figure 9, panels B and D).

**Figure 10:**
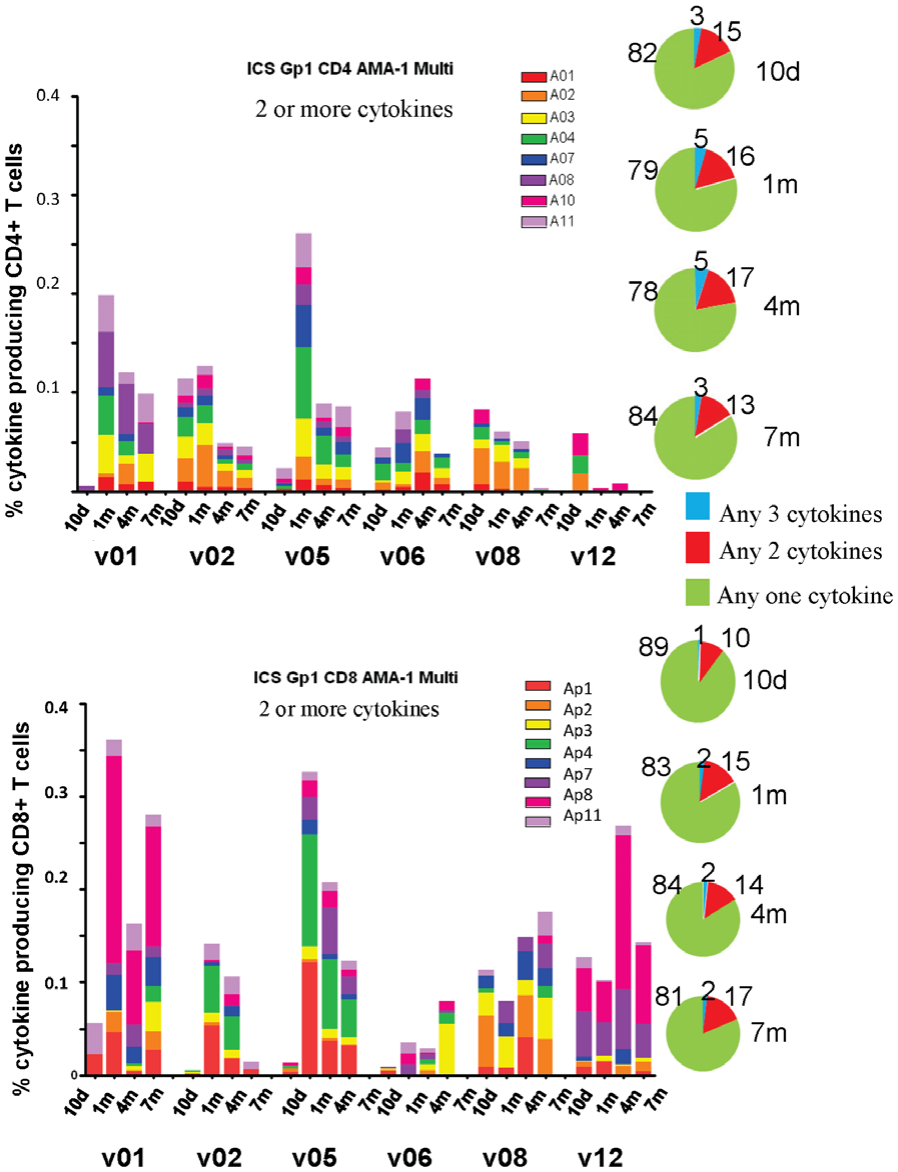
Multifunctional CD4+ and CD8+ T cells of serial bleeds of volunteers in Group 1 with AMA1 peptide pools. **A** and **C**: Multifunctional (any two cytokines) CD4+ and CD8+ T cell activities of each volunteer at 10 d, 1, 4, and 7 m after immunization (subtracting pre-immunization values) are displayed using color-coded AMA1 peptide pools. At 1 month, CD4+ multi responses (geomean 0.072% CD4+ T cells, std dev 94) and CD8+ multi responses (geomean 0.13% CD8+ T cells, std dev 14) were similar. **B** and **D**: Pie charts representing the proportion of the cytokine response indicated by cytokine subsets as shown; numbers on pie charts represent per cent of that subset of the total cells.

Group 1 multifunctional T cell responses to AMA1: as with CSP, CD4+ and CD8+ multifunctional responses to AMA1 were similar to each other at 1 m (geomean 0.13% CD8+ [range 0.0358–0.3612%], 0.07% CD4+ [range 0.0043–0.2613%], Figure 10, panels A and C), but, in contrast to CSP, were not quite statistically significantly different throughout the study (p = 0.062, Anova). As with ELISpot responses, multifunctional T cell responses to AMA1 were higher than for CSP (Figure 10 compared to Figure 9). The frequencies at 1 m represented an increase in the proportion of multifunctional cells relative to 10 d for both CD8+ and CD4+ populations (Figure 10, panels B and D) similar to the increase observed with CSP (Figure 9). Of note, the proportion of multifunctional CD8+ T cell responses remained elevated at 7 m (Figure 10, panels B and D).

Group 2 multifunctional T cell responses to CSP and AMA1: Frequencies of multifunctional CD4+ and CD8+ T cells among gated CD4 and CD8+ T cells were lower than those identified in Group 1 for both CSP (geomeans 0.0045 CD8+ [range 0.0002–0.0309%], 0.009% CD4+ [range 0.0004–0.0387%]) (Figure S4) and AMA1 (geomeans 0.04% CD8+ [range 0.0130–0.0579%], CD4+ 0.022% [range 0.0017–0.1217%]) (Figure S5). When comparing CSP to AMA1, as with Group 1, the frequencies of CD4+ T cell multifunctional responses to CSP (Figure S4, top panel) were lower than to AMA1 (Figure S5, top panel) at 1 m.

#### Median Fluorescence Intensity (MFI)

The MFI of the IFN-γ signal was calculated at 1 m for four IFN-γ-secreting T cell populations: CD4+ triple secretors (IFN-γ + IL-2+ TNFα+), CD8+ triple secretors (IFN-γ+ IL-2+ TNFα+), CD4+ single secretors (IFN-γ+ IL-2− TNFα−) and CD8+ single secretors (IFN-γ+ IL-2− TNFα−). The MFI for the IFN-γ signals of each of these populations was determined by (1) calculating the geometric mean across 12 peptide pools (Cp1 Cp2 Cp6, Cp9, Ap1, Ap2, Ap3, Ap4, Ap7, Ap8, Ap10 and Ap11) for each of the six volunteers in Group 1, and (2) calculating the geomean of these six values, yielding one MFI value for each population. IFN-γ+ MFIs for the two triple secreting CD4+ and CD8+ T cell populations were approximately 7–10-fold higher than for the respective single secreting CD4+ and CD8+ T cell populations (CD4+ triple geomean 6,009, range 699–27,210; single geomean 839, range 267–5359; CD8+ triple geomean 10,415, range 594–36,640; single geomean 1,067, range 277–3960) (Figure S6). The significance of differences between triple and single secretors was calculated in Mann-Whitney U tests using two-tailed probabilities. The IFN-γ+ MFI of CD4+ and CD8+ T cell triple secretors was significantly higher than single secretors (p = 0.002 for CD4+, p = 0.002 for CD8+, respectively), but the difference between CD4+ and CD8+ triple secretors was not significant (p = 0.39). Although the proportion of double and triple cytokine secretors (Figures 9 and 10; panels B and D) was small when compared to the proportion of single cytokine secretors, this increase is greatly magnified by the 7–10-fold increases in MFI. Assuming that IFN-γ + MFI roughly corresponds to the magnitude of the IFN-γ response, these data indicate that triple IFN-γ secreting T cells contributed disproportionately to the overall IFN-γ response and thus may be more potent immune effectors.

#### Concordance between ELISpot and flow cytometry assays

We next examined the results of ELISpot and flow cytometry assays to see if they were generally concordant. To make this determination, we identified the single peptide pool most strongly recognized by ELISpot assay for each volunteer for CSP and for AMA1 at 1 m (Figures 4 and 6, respectively). We compared this to the single peptide pool most strongly recognized by ICS assay for each volunteer for CSP and AMA1 at 1 m (Figures 7 and 8, respectively). Because not all peptide pools were tested by ICS, this comparison was restricted to the peptide pools that were tested. CD8+ T cells were selected over CD4+ T cells for the comparison between ELISpot and flow cytometry because they were the dominant responses measured by the ICS assay.

In five of the six volunteers assessed for CSP responses, and four of the six volunteers assessed for AMA1 responses, the most strongly recognized peptide pool was the same for both ELISpot and ICS assays (CSP: Cp2 for v001, v008 and v012; Cp1 for v005 and v006; AMA1: Ap10 for v001 and v006; Ap4 for v002; Ap8 for v008). For the two non-concordant volunteers assessed for AMA1 responses, the two most strongly recognized peptide pools were the same for both assays, but with the rank order different (Ap1 and Ap4 for v005; Ap8 and Ap10 for v012), also indicating approximate concordance. Only for v002, where ELISpot and ICS were unmatched with regard to CSP, was there discordance; this volunteer most strongly recognized Cp2 by ELISpot, while CD8+ T cells did not recognize this pool by ICS assay. However, CD4+ T cells from this volunteer did recognize Cp2 by ICS assay and could account for the strong response by ELISpot assay. When non-dominant pools were compared between ELISpot and ICS assays, concordance was not as apparent as with the dominant peptide pool. Concordance was also not as apparent when CD4+ T cell responses were compared.

#### Antibody responses

**Figure 11:**
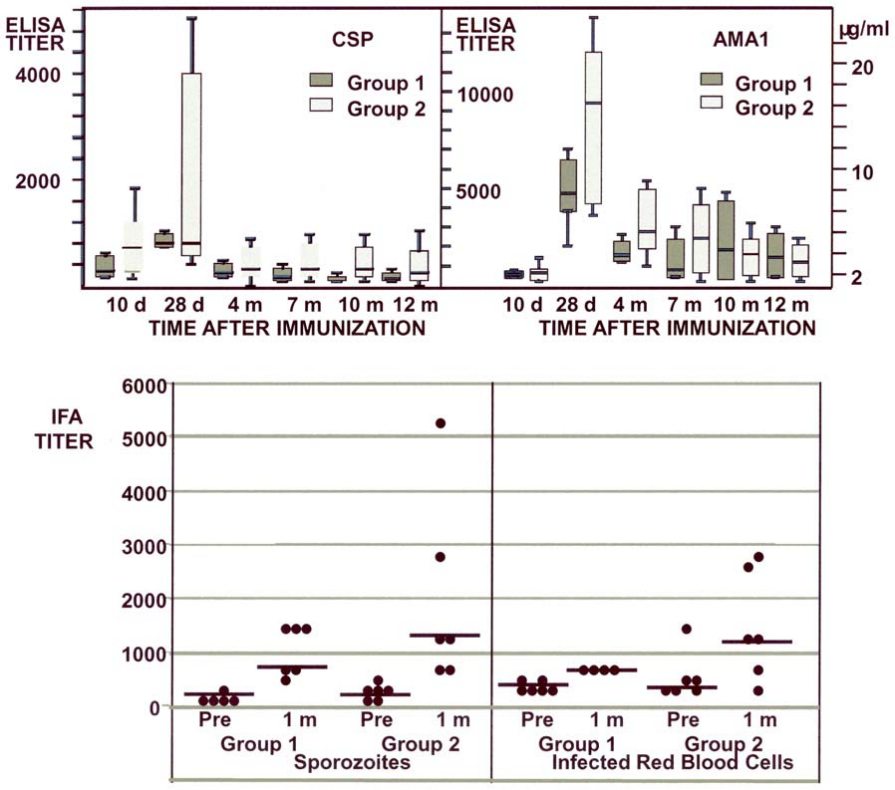
Group 1 and Group 2 anti-CSP and AMA1 antibody responses by ELISA and IFA. **A** and **B**: Box plots of the means and 25% and 75% percentiles of each group at 10 d and 1, 4, 7, 10 and 11–12 m after immunization. The first and third quartiles are the top and base of each box, and the upper and lower bars represent the high and low values respectively. **C**: IFA titers of each group pre-immunization and at 1 month post-immunization against sporozoites (left panel) and infected red blood cells (right panel). Bars represent geomeans.

Antibody responses measured by ELISA to CSP and AMA1 were modest (Figure 11), peaking 1 m after immunization and trending higher in Group 2 than in Group 1 (CSP, p = 0.23; AMA1, p = 0.06, Mann-Whitney U test 2-tailed). Conversion of AMA1 ELISA titers to micrograms of immunoglobulin per milliliter, using quantified controls, revealed a geometric mean antibody concentration for AMA1 of 8.13 µg/mL (range 3.45–12.38 µg/mL) at 1 m post immunization for Group 1 and 17.89 µg/mL (range 7.65–40.34 µg/mL) for Group 2, the latter significantly higher (p = 0.04, Mann-Whitney U test 2-tailed). At 1 m, IFA antibody titers to *P. falciparum* sporozoites or parasitized red blood cells likewise appeared higher in Group 2 at 1 m (but were not significantly different). Growth inhibition assays using parasitized red blood cells were performed to see if the antibodies inhibited the growth of *P. falciparum* in culture, and although inhibitory activity increased marginally post-immunization, particularly in Group 2 where antibody responses were higher, it did not exceed 15% for any volunteer (data not shown).

A summary of all immunogenicity assays, *ex vivo* IFN-γ ELISpot, intracellular cytokine staining (ICS), Median Fluorescence Intensity (MFI), and antibody responses measured by ELISA and IFA, is shown in Table 8.

**Table 8:** Summary of immunogenicity activities.

Grp	Assay	T cell	CSP	p	AMA1	P
1	ELISpot		422±337	0.049@	862±697	0.045@
2			154±203		423±466	
1	ICS	CD8+	0.210±0.14	0.0001*	0.44±0.58	0.003*
		CD4+	0.044±0.075		0.086±0.17	
2		CD8+	0.020±0.12	0.13*	0.15±0.46	0.049*
		CD4+	0.006±0.017		0.035±0.038	
1	ICS	CD8+M	0.06±0.035	0.0016*	0.13±0.14	0.062*
		CD4+M	0.027±0.095		0.072±0.094	
2		CD8+M	0.0045±0.013	0.74*	0.04±0.172	0.26*
		CD4+M	0.009±0.013		0.022±0.060	
1	MFI	CD8+3	10,415±7,221	0.002^$^		
		CD8+1	1,067±432			
		CD4+3			6,009±3.016	0.002^$^
		CD4+1			839±295	
1	ELISA		692±141	0.23@	4,395±1,7188±3 (ug/ml)	0.06@
						0.04@
2			930±2,412		8,478±5,65018±12 (ug/ml)	
			**Sporozoites**		**IRBC**	
1	IFA		806±425	0.19@	640±0	0.066@
2			1,437±1,717		1,140±944	

ELISpot was expressed as sfc/m; ICS as % CD4+ or CD8+ T cells; MFI as geometric mean; ELISA as titer or concentration; IFA as titer. p values were calculated by ANOVA over the course of this study and compare activities in Group 1 and Group 2 (@), CD4+ and CD8+ activities in each group (*), or MFI in CD8 triple cytokine secretors (CD8+3) and CD8+ single cytokine secretors (CD8+1) ($). CD8+M and CD4+M are multifunctional T cells (secreting two or more cytokines).

## Discussion

### Interpretation

The first objective of this dose-escalation study was to assess the safety and reactogenicity of the NMRC-M3V-Ad-PfCA vaccine. This study demonstrated that NMRC-M3V-Ad-PfCA given as a single dose of 2×10^10^ pu or as the five-fold higher dose of 1×10^11^ pu was safe and well tolerated in malaria-naïve adult volunteers. One volunteer receiving the higher dose experienced severe chills, myalgia, and headache and additional symptoms eight hours following immunization that resolved without intervention. Transient, clinically unapparent neutropenia developed consistently in most volunteers during the first several days following immunization, with one Grade 3 neutrophil decrease occurring with the higher dose.

The second objective of this study was to measure the immunogenicity of the 2×10^10^ pu and 1×10^11^ pu doses. This vaccine induced strong IFN-γ responses against both the CSP and AMA1 antigens in healthy, seronegative adults as demonstrated by *ex vivo* ELISpot assay, with CD8+ T cell responses predominating over CD4+ T cell responses in ICS assays. Both CD4+ and CD8+ T cell responses generally peaked at 1 m after immunization and persisted for up to 12 m in many volunteers. Although pool-specific responses for a given volunteer were consistent over time, responses varied among volunteers and between antigens, likely reflecting differences in genetic restriction. The dominant one or two pools identified by *ex vivo* ELISpot assay for each volunteer at 1 m post immunization were also dominant for the CD8+ T cell responses identified by ICS assay in nearly all (11/12) cases, indicating that these pools likely contained immunodominant CD8 epitopes.

Our results constitute the first demonstration of a malaria vaccine inducing a predominant CD8+ T cell (relative to CD4+ T cell) response in humans as detected by ICS IFN-γ assay, although cytotoxic T lymphocyte responses, presumably mediated by CD8+ T cells, have been demonstrated following the administration of DNA-vectored [47] and virally vectored malaria vaccines [53], [54]. This result underscores the potential value of adenovectored vaccines for eliciting mechanisms that may mediate protective responses against infectious agents such as malaria where CD8+ T cell responses are proposed to be critical effectors of protective immunity. Similar responses have been induced by adenovectored malaria antigens in animal models [24] and adenovectored HIV antigens in humans [41].

*Ex vivo* IFN-γ responses to CSP and AMA1 by both ELISpot and ICS assays were statistically significantly higher when the vaccine was administered at 1×10^10^ pu of each construct relative to a five-fold higher dose. These results are generally concordant with other clinical studies of adenovirus (serotype 5)-vectored vaccines showing little change or a drop in responses in Ad5 seronegative individuals comparing 10^10^ pu to 10^11^ pu, with some variation depending on the antigen tested [41], [55], [56]. The inverse relationship between ELISpot responses and dose demonstrated in our study may reflect an inverse relationship between the frequency of multifunctional T cells and antigen load [57]. Because the loss of response at the higher dose was statistically significant and is supported by other studies, we selected the lower dose as optimal for further clinical development.

Interestingly, when ELISpot assays were performed following depletion of either CD4+ or CD8+ T cells, most responses to individual peptide pools were CD4+ T cell-dependent (17 of 39 assays, or CD4+ and CD8+ T cell-dependent (19 of 39 assays), and in only three cases dependent on CD8+ alone. The paucity of responses dependent solely on CD8+ T cells despite the predominance of CD8+ T cell responses may indicate that CD8+ T cell epitopes are nested within or adjacent to CD4+ T cell epitopes or reflect the importance of CD4+ T cell help. In one volunteer (v001), CD8+ depletions increased IFN-γ levels (observed by others [58]), possibly reflecting the presence of regulatory T cells of the CD8+ phenotype that were inhibiting autologous T cell responses and which potentially might negatively affect vaccine efficacy [59].This is consistent with other findings that CD8+ T cell responses depend on CD4+ T cells suggesting that CD4+ and CD8+ T epitopes may overlap [60], [61].

There was a tendency for the volunteers in each group to recognize more pools from AMA1 than CSP (Table 6). This likely reflects the longer length of the AMA1 construct compared with CSP construct (622 vs. 333 amino acids, respectively), and may explain why summed responses to AMA1 were larger than responses to CSP. We found, like others [62], that the N-terminal region of CSP (spanned by Cp1 and Cp2) was highly immunogenic and induced both CD4+ and CD8+ T cell responses. This region contains CD8+-restricted epitopes (amino acids 1–10, 7–16 [42], [43], [63]) and CD4+-restricted epitopes (amino acids 1–16 [60], 51–70 [44], [60]). However, C-terminal peptide pools Cp6 and Cp9 also induced strong T cell responses and contain previously described T cell epitopes CST3 [64], Th2R and Th3R [65], and several others [44]. This may be important when comparing this vaccine to the protective vaccine RTS,S, where the CSP N-terminal is absent [66]. The AMA1 peptide pools recognized by ELISpot or ICS in this study overlapped peptides recognized by proliferating T cells in a study in Africa [46], including a peptide associated with a lower risk of parasitemia [67] contained within peptide 13 in Ap5.

The NMRC-M3V-Ad-PfCA vaccine induced a low frequency of multifunctional CD4+ and CD8+ T cell responses (cells producing any 2 or more cytokines), with differences in MFI indicating that the triple secreting CD8+ T cells produced 7–10-fold more IFN-γ than single secretors, consistent with other studies [57]. Thus, even though triple secretors remained less than 10% of cells secreting IFN-γ, TNF-α and/or IL2, their contribution to overall IFN-γ production was disproportionately large. Such cells may be associated with protective immunity (leishmaniasis, HIV) although the role of either CD4+ or CD8+ multifunctional T cells in malaria, though suggested [25], [68], [69], remains to be proven. Adenovectored vaccines have induced multifunctional CD8+ T cell malaria and HIV-1 responses in mice and macaques [70], [71], [72].

Antibody titers were low as measured by ELISA, and did not significantly inhibit growth in vitro using 15% inhibition as a positive threshold. Previous studies suggested that 15% growth inhibition required approximately 30 µg/mL of purified human anti-AMA1 antibodies [73], above the levels achieved in this trial (8.13 µg/mL–17.89 µg/mL). Antibody titers to sporozoites as measured by IFA were lower than the IFA titers commonly achieved with the RTS,S vaccine [18], but antibody titers to infected red blood cells were similar to the IFA titers achieved by a recombinant AMA1 vaccine [74] in another human trial. When the low dose and high dose groups were compared, there was a trend toward higher antibody responses in the high dose group that reached significance when AMA1 antibody in micrograms per milliliter was compared, the reverse of what was observed for T cell responses. Based on animal studies [75] and a recently published clinical trial [76], superior antibody responses might be obtained if this adenovectored vaccine were administered as part of an appropriate prime-boost regimen, without losing potency for inducing T cell responses.

Irradiated *P. falciparum* sporozoites induce sterile protective immunity in humans, and CSP is a dominant protective antigen [77], [78]. ELISpot responses from irradiated sporozoite-protected volunteers using CSP peptide pools were low, 41.6±20.1 sfc/m [79] compared with 422±337 sfc/m in this study. Antibody responses from irradiated-sporozoite protected volunteers were also low [80], [81] compared to the vaccine-induced antibody activities induced in this referenced study [80].

### Generalizability

This study indicates the potential value of adenovectored malaria vaccines to induce responses thought to be related to protection, particularly for inducing the CD8+ immune responses likely critical for targeting liver stage parasites, although CD8+ T cell functionality might be dependent on the kinetics of antigen expression and presentation [82], [83]. Adenovectors expressing CSP, AMA1 as well as additional antigens should be tested in humans alone and in prime-boost regimens for efficacy against experimental sporozoite challenge. One interesting prime-boost strategy would be to combine adenovectors with protein-based vaccines. This regimen may provide high-level protection against malaria due to induction of strong CD8+/CD4+ T cell responses and strong antibody responses as observed in preclinical studies [48].

### Limitations

The main conclusions of this study – the predominance of CD8+ over CD4+ T cell responses and the inverse relationship between dose and magnitude of ELISpot responses – are strengthened by the fact that they applied equally to both antigens studied, CSP and AMA1. However, it is possible that the findings will not extend to other malaria antigens. T cell responses were statistically significantly stronger in the lower dose group, while antibody responses trended stronger in the high dose group, indicating a potential trade-off between achieving cellular and humor immunity; thus it may not be possible to achieve both simultaneously using adenovirus-vectored vaccines, although heterologous prime-boost vaccines could circumvent this limitation. Notably, the study was limited to Ad5 seronegative volunteers. Because neutralizing antibodies could diminish the effect of the vaccine on the recipients, and because the primary objective of this first-in-humans study was to assess safety, it was important to restrict the study to seronegatives in order to maximize the expression of adverse events. Whether a seropositive status, however, could blunt adverse reactions or immune responses will need to be evaluated.

### Overall evidence

This vaccine trial supports the safety and tolerability of adenovirus vaccines within the range tested 1×10^10^–1×10^11^ pu, and indicates that two antigens may be safely and effectively combined, and the first evidence (see also Tamminga et al) that adenovirus-vectored malaria vaccines induce robust IFN-γ CD8+ T cell responses in humans.

## Supporting Information

Figure S1 **Gating strategy to separate CD4+ and CD8+ T cell populations for analysis of cytokine secretion.** Histograms were used to determine the total production of IFN-γ, IL-2 and TNFα for the CD4+ or CD8+ populations (a total of 6 histograms). Boolean Gates are used to determine cells producing combinations of more than one cytokine, or one cytokine only.
(TIFF)

Figure S2 **Total ICS CD4+ and CD8+ IFN-γ+ activity of serial bleeds of volunteers in Group 2 with four dominant CSP peptide pools.** Four Cp peptide pools that were most strongly recognized in ELISpot assays were used to determine ICS CD4+ and CD8+ IFN-γ+ activity with Group 2 volunteers. The ICS CD4+ and CD8+ T cells activities of each volunteer at pre-immunization, 10 d and 1, 4, and 7 m are displayed using color-coded CSP peptide pools. Scales for each phenotype have been equalized. *Not tested.
(TIFF)

Figure S3 **Total ICS CD4+ and CD8+ IFN-γ+ activity of serial bleeds of volunteers in Group 2 with 8 dominant AMA1 peptide pools.** Eight Ap peptide pools that were most strongly recognized in ELISpot assays were used to determine ICS CD4+ and CD8+ IFN-γ+ activity with Group 2 volunteers. The ICS CD4+ and CD8+ T cells activities of each volunteer at pre-immunization, 10 d and 1, 4, and 7 m are displayed using color-coded AMA1 peptide pools. Scales for each phenotype have been equalized. *Not tested.
(TIFF)

Figure S4 **ICS multifunctional CD4+ and CD8+ T cells of serial bleeds of volunteers in Group 2 following PBMC stimulation with four dominant CSP peptide pools.** The multifunctional (any two cytokines) CD4+ and CD8+ T cells activity of each volunteer at 10 d, 1, 4, and 7 m after immunization are displayed using color-coded CSP peptide pools. *Not tested.
(TIF)

Figure S5 **Multifunctional CD4+ and CD8+ T cells of serial bleeds of volunteers in Group 2 following PBMC stimulation with eight dominant AMA1 peptide pools.** The multifunctional (any two cytokines) CD4+ and CD8+ T cell activities of each volunteer at 10 d, 1, 4, and 7 m after immunization are displayed using color-coded AMA1 peptide pools. *Not tested.
(TIF)

Figure S6 **Median Fluorescence Intensity of volunteers at 1 m after immunization.** The Median Fluorescent Intensity (MFI) of the IFN-γ signal for cytokine triple secretors and single secretors was measured 1 m after immunization in CD8+ (left panel) and CD4+ (right panel) T cell populations. Data from Group 1 and Group 2 and from CSP and AMA1 are combined. The boxes represent 25^th^ to 75^th^ percentile, the bar within the box the mean, the whiskers extend to 10^th^ and 90^th^ percentiles. The significance of differences between activities was calculated using a two-tailed Mann-Whitney U tests. Triple secretors appeared to show a significant 7–10-fold higher signal intensity than single secretors for CD4+ and CD8+ T cells, but CD4+ T cell triple secretors appeared similar to CD8+ triple secretors.
(TIFF)

Table S1 **Unsolicited adverse events experienced by volunteers Days 0–28.** Unsolicited adverse events were recorded for 28 days following each immunization. Because of the theoretical, even if remote, possibility of reversion of the vaccine to replication competence, attention was paid to unexpected symptoms that might have reflected adenovirus infection. However, the few clinical syndromes observed in volunteers following immunization that were consistent with adenovirus infection (upper and lower respiratory infection, enteritis, urinary tract infection) bore no particular relationship to immunization in terms of timing and appeared to reflect background rates in the community.
(DOC)

Checklist S1 (DOC)

Protocol S1 **Clinical protocol for NMRC-M3V = Ad-PfCA Vaccine.**
(DOC)

## References

[1] Clyde DF (1975). Immunization of man against falciparum and vivax malaria by use of attenuated sporozoites.. Am J Trop Med Hyg.

[2] Sigler CI, Leland P, Hollingdale MR (1984). In vitro infectivity of irradiated Plasmodium berghei sporozoites to cultured hepatoma cells.. Am J Trop Med Hyg.

[3] Nussenzweig RS, Vanderberg J, Most H, Orton C (1967). Protective immunity produced by the injection of x-irradiated sporozoites of plasmodium berghei.. Nature.

[4] Oliveira GA, Kumar KA, Calvo-Calle JM, Othoro C, Altszuler D (2008). Class II-restricted protective immunity induced by malaria sporozoites.. Infect Immun.

[5] Doolan DL, Martinez-Alier N (2006). Immune response to pre-erythrocytic stages of malaria parasites.. Curr Mol Med.

[6] Jobe O, Donofrio G, Sun G, Liepinsh D, Schwenk R (2009). Immunization with radiation-attenuated Plasmodium berghei sporozoites induces liver cCD8alpha+DC that activate CD8+T cells against liver-stage malaria.. PLoS ONE.

[7] Fandeur T, Dubois P, Gysin J, Dedet JP, da Silva LP (1984). In vitro and in vivo studies on protective and inhibitory antibodies against Plasmodium falciparum in the Saimiri monkey.. J Immunol.

[8] Beeson JG, Osier FH, Engwerda CR (2008). Recent insights into humoral and cellular immune responses against malaria.. Trends Parasitol.

[9] Marsh K, Kinyanjui S (2006). Immune effector mechanisms in malaria.. Parasite Immunol.

[10] Sedegah M, Brice GT, Rogers WO, Doolan DL, Charoenvit Y (2002). Persistence of protective immunity to malaria induced by DNA priming and poxvirus boosting: characterization of effector and memory CD8(+)-T-cell populations.. Infect Immun.

[11] Jiang G, Charoenvit Y, Moreno A, Baraceros MF, Banania G (2007). Induction of multi-antigen multi-stage immune responses against Plasmodium falciparum in rhesus monkeys, in the absence of antigen interference, with heterologous DNA prime/poxvirus boost immunization.. Malar J.

[12] Ockenhouse CF, Sun PF, Lanar DE, Wellde BT, Hall BT (1998). Phase I/IIa safety, immunogenicity, and efficacy trial of NYVAC-Pf7, a pox-vectored, multiantigen, multistage vaccine candidate for Plasmodium falciparum malaria.. J Infect Dis.

[13] Bojang KA, Olodude F, Pinder M, Ofori-Anyinam O, Vigneron L (2005). Safety and immunogenicty of RTS,S/AS02A candidate malaria vaccine in Gambian children.. Vaccine.

[14] Aponte JJ, Aide P, Renom M, Mandomando I, Bassat Q (2007). Safety of the RTS,S/AS02D candidate malaria vaccine in infants living in a highly endemic area of Mozambique: a double blind randomised controlled phase I/IIb trial.. Lancet.

[15] Kester KE, McKinney DA, Tornieporth N, Ockenhouse CF, Heppner DG (2007). A phase I/IIa safety, immunogenicity, and efficacy bridging randomized study of a two-dose regimen of liquid and lyophilized formulations of the candidate malaria vaccine RTS,S/AS02A in malaria-naive adults.. Vaccine.

[16] Sun P, Schwenk R, White K, Stoute JA, Cohen J (2003). Protective immunity induced with malaria vaccine, RTS,S, is linked to Plasmodium falciparum circumsporozoite protein-specific CD4+ and CD8+ T cells producing IFN-gamma.. J Immunol.

[17] Mettens P, Dubois PM, Demoitie MA, Bayat B, Donner MN (2008). Improved T cell responses to Plasmodium falciparum circumsporozoite protein in mice and monkeys induced by a novel formulation of RTS,S vaccine antigen.. Vaccine.

[18] Kester KE, Cummings JF, Ofori-Anyinam O, Ockenhouse CF, Krzych U (2009). Randomized, double-blind, phase 2a trial of falciparum malaria vaccines RTS,S/AS01B and RTS,S/AS02A in malaria-naive adults: safety, efficacy, and immunologic associates of protection.. J Infect Dis.

[19] Li S, Locke E, Bruder J, Clarke D, Doolan DL (2007). Viral vectors for malaria vaccine development.. Vaccine.

[20] Limbach KJ, Richie TL (2009). Viral vectors in malaria vaccine development.. Parasite Immunol.

[21] Barouch DH, Pau MG, Custers JH, Koudstaal W, Kostense S (2004). Immunogenicity of recombinant adenovirus serotype 35 vaccine in the presence of pre-existing anti-Ad5 immunity.. J Immunol.

[22] Draper SJ, Moore AC, Goodman AL, Long CA, Holder AA (2008). Effective induction of high-titer antibodies by viral vector vaccines.. Nat Med.

[23] Draper SJ, Goodman AL, Biswas S, Forbes EK, Moore AC (2009). Recombinant viral vaccines expressing merozoite surface protein-1 induce antibody- and T cell-mediated multistage protection against malaria.. Cell Host Microbe.

[24] Rodriguez A, Goudsmit J, Companjen A, Mintardjo R, Gillissen G (2008). Impact of recombinant adenovirus serotype 35 priming versus boosting of a Plasmodium falciparum protein: characterization of T- and B-cell responses to liver-stage antigen 1.. Infect Immun.

[25] Darrah PA, Patel DT, De Luca PM, Lindsay RW, Davey DF (2007). Multifunctional TH1 cells define a correlate of vaccine-mediated protection against Leishmania major.. Nat Med.

[26] Russell WC (2009). Adenoviruses: update on structure and function.. J Gen Virol.

[27] Zaiss AK, Machado HB, Herschman HR (2009). The influence of innate and pre-existing immunity on adenovirus therapy.. J Cell Biochem.

[28] Cheng C, Gall JG, Kong WP, Sheets RL, Gomez PL (2007). Mechanism of ad5 vaccine immunity and toxicity: fiber shaft targeting of dendritic cells.. PLoS Pathog.

[29] Lore K, Adams WC, Havenga MJ, Precopio ML, Holterman L (2007). Myeloid and plasmacytoid dendritic cells are susceptible to recombinant adenovirus vectors and stimulate polyfunctional memory T cell responses.. J Immunol.

[30] Stoute JA, Slaoui M, Heppner DG, Momin P, Kester KE (1997). A preliminary evaluation of a recombinant circumsporozoite protein vaccine against Plasmodium falciparum malaria. RTS,S Malaria Vaccine Evaluation Group.. N Engl J Med.

[31] Narum DL, Thomas AW (1994). Differential localization of full-length and processed forms of PF83/AMA-1 an apical membrane antigen of Plasmodium falciparum merozoites.. Mol Biochem Parasitol.

[32] Stowers AW, Kennedy MC, Keegan BP, Saul A, Long CA (2002). Vaccination of monkeys with recombinant Plasmodium falciparum apical membrane antigen 1 confers protection against blood-stage malaria.. Infect Immun.

[33] Polley SD, Mwangi T, Kocken CH, Thomas AW, Dutta S (2004). Human antibodies to recombinant protein constructs of Plasmodium falciparum Apical Membrane Antigen 1 (AMA1) and their associations with protection from malaria.. Vaccine.

[34] Silvie O, Franetich JF, Charrin S, Mueller MS, Siau A (2004). A role for apical membrane antigen 1 during invasion of hepatocytes by Plasmodium falciparum sporozoites.. J Biol Chem.

[35] Peduzzi E, Westerfeld N, Zurbriggen R, Pluschke G, Daubenberger CA (2008). Contribution of influenza immunity and virosomal-formulated synthetic peptide to cellular immune responses in a phase I subunit malaria vaccine trial.. Clin Immunol.

[36] Okitsu SL, Silvie O, Westerfeld N, Curcic M, Kammer AR (2007). A virosomal malaria peptide vaccine elicits a long-lasting sporozoite-inhibitory antibody response in a phase 1a clinical trial.. PLoS One.

[37] Sprangers MC, Lakhai W, Koudstaal W, Verhoeven M, Koel BF (2003). Quantifying adenovirus-neutralizing antibodies by luciferase transgene detection: addressing preexisting immunity to vaccine and gene therapy vectors.. J Clin Microbiol.

[38] Bruder JT, Angov E, Limbach KJ, Richie TL (2010). Molecular vaccines for malaria.. Hum Vaccin.

[39] Brough DE, Lizonova A, Hsu C, Kulesa VA, Kovesdi I (1996). A gene transfer vector-cell line system for complete functional complementation of adenovirus early regions E1 and E4.. J Virol.

[40] Reyes-Sandoval A, Sridhar S, Berthoud T, Moore AC, Harty JT (2008). Single-dose immunogenicity and protective efficacy of simian adenoviral vectors against Plasmodium berghei.. Eur J Immunol.

[41] Harro CD, Robertson MN, Lally MA, O'Neill LD, Edupuganti S (2009). Safety and immunogenicity of adenovirus-vectored near-consensus HIV type 1 clade B gag vaccines in healthy adults.. AIDS Res Hum Retroviruses.

[42] Doolan DL, Hoffman SL, Southwood S, Wentworth PA, Sidney J (1997). Degenerate cytotoxic T cell epitopes from P. falciparum restricted by multiple HLA-A and HLA-B supertype alleles.. Immunity.

[43] Blum-Tirouvanziam U, Servis C, Habluetzel A, Valmori D, Men Y (1995). Localization of HLA-A2.1-restricted T cell epitopes in the circumsporozoite protein of Plasmodium falciparum.. J Immunol.

[44] Good MF, Pombo D, Quakyi IA, Riley EM, Houghten RA (1988). Human T-cell recognition of the circumsporozoite protein of Plasmodium falciparum: immunodominant T-cell domains map to the polymorphic regions of the molecule.. Proc Natl Acad Sci U S A.

[45] Zevering Y, Houghten RA, Frazer IH, Good MF (1990). Major population differences in T cell response to a malaria sporozoite vaccine candidate.. Int Immunol.

[46] Lal AA, Hughes MA, Oliveira DA, Nelson C, Bloland PB (1996). Identification of T-cell determinants in natural immune responses to the Plasmodium falciparum apical membrane antigen (AMA-1) in an adult population exposed to malaria.. Infect Immun.

[47] Wang R, Epstein J, Baraceros FM, Gorak EJ, Charoenvit Y (2001). Induction of CD4(+) T cell-dependent CD8(+) type 1 responses in humans by a malaria DNA vaccine.. Proc Natl Acad Sci U S A.

[48] Stewart VA, McGrath SM, Dubois PM, Pau MG, Mettens P (2007). Priming with an adenovirus 35-circumsporozoite protein (CS) vaccine followed by RTS,S/AS01B boosting significantly improves immunogenicity to Plasmodium falciparum CS compared to that with either malaria vaccine alone.. Infect Immun.

[49] Spring MD, Cummings JF, Ockenhouse CF, Dutta S, Reidler R (2009). Phase 1/2a study of the malaria vaccine candidate apical membrane antigen-1 (AMA-1) administered in adjuvant system AS01B or AS02A.. PLoS One.

[50] Charoenvit Y, Mellouk S, Cole C, Bechara R, Leef MF (1991). Monoclonal, but not polyclonal, antibodies protect against Plasmodium yoelii sporozoites.. J Immunol.

[51] Bergmann-Leitner ES, Duncan EH, Mullen GE, Burge JR, Khan F (2006). Critical evaluation of different methods for measuring the functional activity of antibodies against malaria blood stage antigens.. Am J Trop Med Hyg.

[52] Neter J (1996). Applied Linear Statistical Methods..

[53] Moorthy VS, Imoukhuede EB, Keating S, Pinder M, Webster D (2004). Phase 1 evaluation of 3 highly immunogenic prime-boost regimens, including a 12-month reboosting vaccination, for malaria vaccination in Gambian men.. J Infect Dis.

[54] Vuola JM, Keating S, Webster DP, Berthoud T, Dunachie S (2005). Differential immunogenicity of various heterologous prime-boost vaccine regimens using DNA and viral vectors in healthy volunteers.. J Immunol.

[55] Harro C, Sun X, Stek JE, Leavitt RY, Mehrotra DV (2009). Safety and immunogenicity of the Merck adenovirus serotype 5 (MRKAd5) and MRKAd6 human immunodeficiency virus type 1 trigene vaccines alone and in combination in healthy adults.. Clin Vaccine Immunol.

[56] Priddy FH, Brown D, Kublin J, Monahan K, Wright DP (2008). Safety and immunogenicity of a replication-incompetent adenovirus type 5 HIV-1 clade B gag/pol/nef vaccine in healthy adults.. Clin Infect Dis.

[57] Seder RA, Darrah PA, Roederer M (2008). T-cell quality in memory and protection: implications for vaccine design.. Nat Rev Immunol.

[58] Kaufhold RM, Field JA, Caulfield MJ, Wang S, Joseph H (2005). Memory T-cell response to rotavirus detected with a gamma interferon enzyme-linked immunospot assay.. J Virol.

[59] Minigo G, Woodberry T, Piera KA, Salwati E, Tjitra E (2009). Parasite-dependent expansion of TNF receptor II-positive regulatory T cells with enhanced suppressive activity in adults with severe malaria.. PLoS Pathog.

[60] Doolan DL, Southwood S, Chesnut R, Appella E, Gomez E (2000). HLA-DR-promiscuous T cell epitopes from Plasmodium falciparum pre-erythrocytic-stage antigens restricted by multiple HLA class II alleles.. J Immunol.

[61] Sedegah M, Kim Y, Peters B, McGrath S, Ganeshan H Identification and localization of minimal MHC-restricted CD8+ T cell epitopes within the Plasmodium falciparum AMA1 protein.. Malar J.

[62] Bongfen SE, Ntsama PM, Offner S, Smith T, Felger I (2009). The N-terminal domain of Plasmodium falciparum circumsporozoite protein represents a target of protective immunity.. Vaccine.

[63] Wang R, Doolan DL, Le TP, Hedstrom RC, Coonan KM (1998). Induction of antigen-specific cytotoxic T lymphocytes in humans by a malaria DNA vaccine.. Science.

[64] Nardin EH, Calvo-Calle JM, Oliveira GA, Nussenzweig RS, Schneider M (2001). A totally synthetic polyoxime malaria vaccine containing Plasmodium falciparum B cell and universal T cell epitopes elicits immune responses in volunteers of diverse HLA types.. J Immunol.

[65] Lockyer MJ, Marsh K, Newbold CI (1989). Wild isolates of Plasmodium falciparum show extensive polymorphism in T cell epitopes of the circumsporozoite protein.. Mol Biochem Parasitol.

[66] Stoute JA, Kester KE, Krzych U, Wellde BT, Hall T (1998). Long-term efficacy and immune responses following immunization with the RTS,S malaria vaccine.. J Infect Dis.

[67] Udhayakumar V, Kariuki S, Kolczack M, Girma M, Roberts JM (2001). Longitudinal study of natural immune responses to the Plasmodium falciparum apical membrane antigen (AMA-1) in a holoendemic region of malaria in western Kenya: Asembo Bay Cohort Project VIII.. Am J Trop Med Hyg.

[68] Huaman MC, Mullen GE, Long CA, Mahanty S (2009). Plasmodium falciparum apical membrane antigen 1 vaccine elicits multifunctional CD4 cytokine-producing and memory T cells.. Vaccine.

[69] Reyes-Sandoval A, Berthoud T, Alder N, Siani L, Gilbert SC Prime-boost immunization with adenoviral and modified vaccinia virus Ankara vectors enhances the durability and polyfunctionality of protective malaria CD8+ T-cell responses.. Infect Immun.

[70] Shott JP, McGrath SM, Pau MG, Custers JH, Ophorst O (2008). Adenovirus 5 and 35 vectors expressing Plasmodium falciparum circumsporozoite surface protein elicit potent antigen-specific cellular IFN-gamma and antibody responses in mice.. Vaccine.

[71] Liu J, Ewald BA, Lynch DM, Denholtz M, Abbink P (2008). Magnitude and phenotype of cellular immune responses elicited by recombinant adenovirus vectors and heterologous prime-boost regimens in rhesus monkeys.. J Virol.

[72] Bruna-Romero O, Rocha CD, Tsuji M, Gazzinelli RT (2004). Enhanced protective immunity against malaria by vaccination with a recombinant adenovirus encoding the circumsporozoite protein of Plasmodium lacking the GPI-anchoring motif.. Vaccine.

[73] Pierce MA, Ellis RD, Martin LB, Malkin E, Tierney E Phase 1 safety and immunogenicity trial of the Plasmodium falciparum blood-stage malaria vaccine AMA1-C1/ISA 720 in Australian adults.. Vaccine.

[74] Malkin EM, Diemert DJ, McArthur JH, Perreault JR, Miles AP (2005). Phase 1 clinical trial of apical membrane antigen 1: an asexual blood-stage vaccine for Plasmodium falciparum malaria.. Infect Immun.

[75] Bruder JT, Stefaniak ME, Patterson NB, Chen P, Konovalova S (2010). Adenovectors induce functional antibodies capable of potent inhibition of blood stage malaria parasite growth.. Vaccine.

[76] Koup RA, Roederer M, Lamoreaux L, Fischer J, Novik L (2010). Priming immunization with DNA augments immunogenicity of recombinant adenoviral vectors for both HIV-1 specific antibody and T-cell responses.. PLoS ONE.

[77] Kumar KA, Sano G, Boscardin S, Nussenzweig RS, Nussenzweig MC (2006). The circumsporozoite protein is an immunodominant protective antigen in irradiated sporozoites.. Nature.

[78] Hoffman SL, Goh LM, Luke TC, Schneider I, Le TP (2002). Protection of humans against malaria by immunization with radiation-attenuated Plasmodium falciparum sporozoites.. J Infect Dis.

[79] Doolan DL, Southwood S, Freilich DA, Sidney J, Graber NL (2003). Identification of Plasmodium falciparum antigens by antigenic analysis of genomic and proteomic data.. Proc Natl Acad Sci U S A.

[80] Egan JE, Hoffman SL, Haynes JD, Sadoff JC, Schneider I (1993). Humoral immune responses in volunteers immunized with irradiated Plasmodium falciparum sporozoites.. Am J Trop Med Hyg.

[81] Hollingdale MR, Appiah A, Leland P, do Rosario VE, Mazier D (1990). Activity of human volunteer sera to candidate Plasmodium falciparum circumsporozoite protein vaccines in the inhibition of sporozoite invasion assay of human hepatoma cells and hepatocytes.. Trans R Soc Trop Med Hyg.

[82] Lindsay RW, Darrah PA, Quinn KM, Wille-Reece U, Mattei LM CD8+ T cell responses following replication-defective adenovirus serotype 5 immunization are dependent on CD11c+ dendritic cells but show redundancy in their requirement of TLR and nucleotide-binding oligomerization domain-like receptor signaling.. J Immunol.

[83] Geiben-Lynn R, Greenland JR, Frimpong-Boateng K, Letvin NL (2008). Kinetics of recombinant adenovirus type 5, vaccinia virus, modified vaccinia ankara virus, and DNA antigen expression in vivo and the induction of memory T-lymphocyte responses.. Clin Vaccine Immunol.

