# Supplementary figures and images for: Adenovirus 5-Vectored P. falciparum Vaccine Expressing CSP and AMA1. Part A: Safety and Immunogenicity in Seronegative Adults
Source: PLoS One. 2011 Oct 7;6(10):e24586. doi: 10.1371/journal.pone.0024586 (PMC3189181; doi:10.1371/journal.pone.0024586)

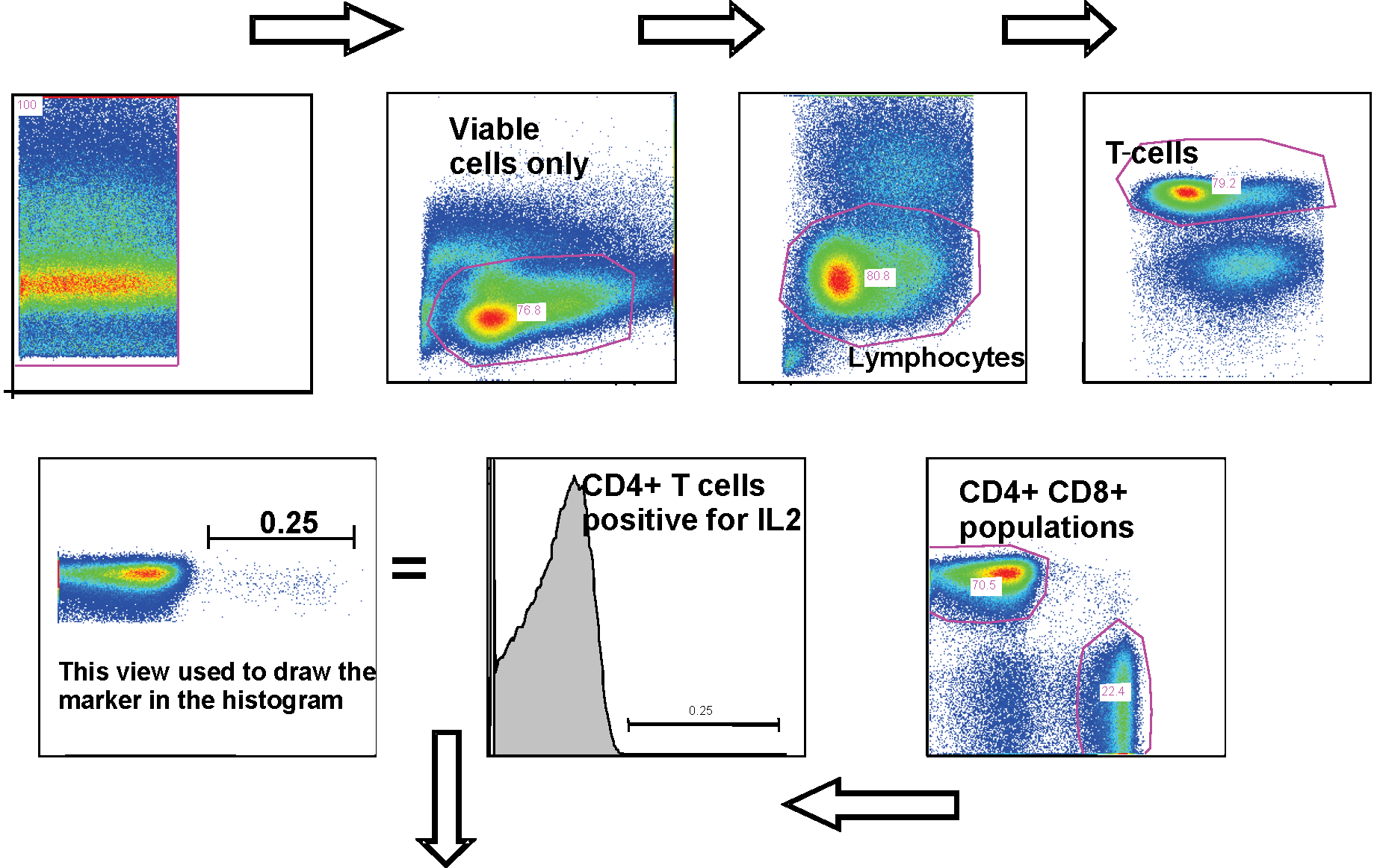

Supplement: Figure S1 — Gating strategy to separate CD4+ and CD8+ T cell populations for analysis of cytokine secretion. Histograms were used to determine the total production of IFN-γ, IL-2 and TNFα for the CD4+ or CD8+ populations (a total of 6 histograms). Boolean Gates are used to determine cells producing combinations of more than one cytokine, or one cytokine only. (TIFF) [file pone.0024586.s001.tif]

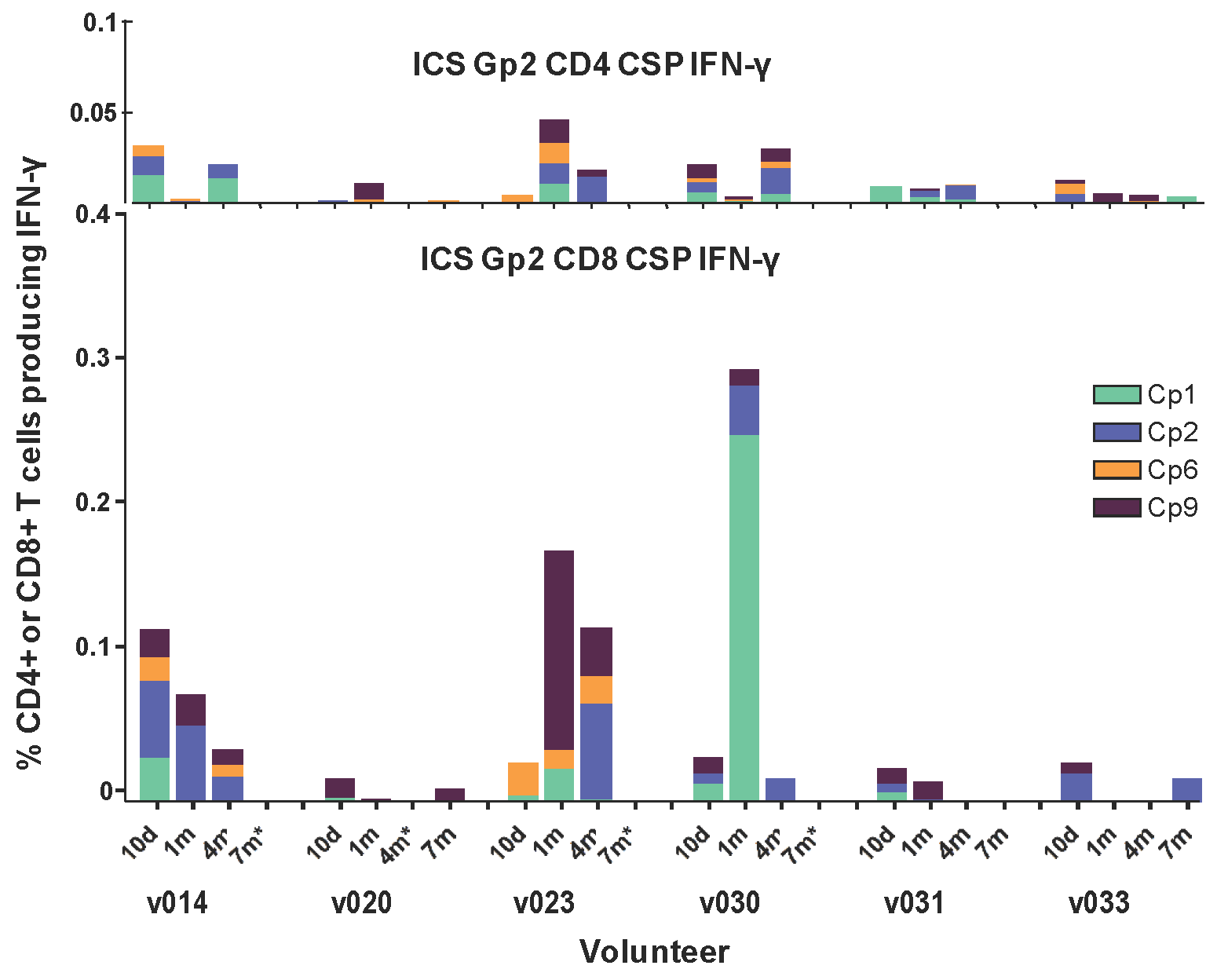

Supplement: Figure S2 — Total ICS CD4+ and CD8+ IFN-γ+ activity of serial bleeds of volunteers in Group 2 with four dominant CSP peptide pools. Four Cp peptide pools that were most strongly recognized in ELISpot assays were used to determine ICS CD4+ and CD8+ IFN-γ+ activity with Group 2 volunteers. The ICS CD4+ and CD8+ T cells activities of each volunteer at pre-immunization, 10 d and 1, 4, and 7 m are displayed using color-coded CSP peptide pools. Scales for each phenotype have been equalized. *Not tested. (TIFF) [file pone.0024586.s002.tif]

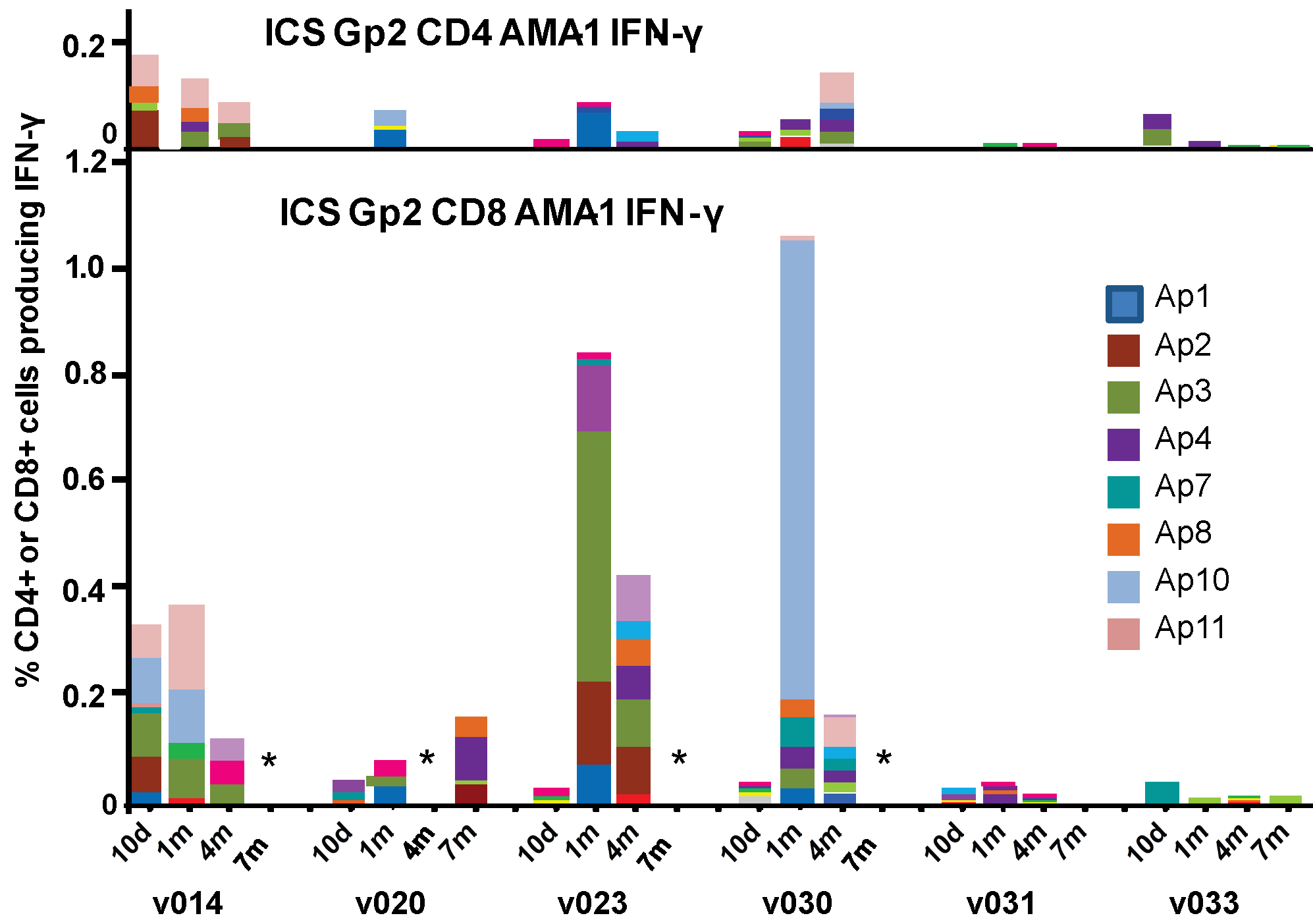

Supplement: Figure S3 — Total ICS CD4+ and CD8+ IFN-γ+ activity of serial bleeds of volunteers in Group 2 with 8 dominant AMA1 peptide pools. Eight Ap peptide pools that were most strongly recognized in ELISpot assays were used to determine ICS CD4+ and CD8+ IFN-γ+ activity with Group 2 volunteers. The ICS CD4+ and CD8+ T cells activities of each volunteer at pre-immunization, 10 d and 1, 4, and 7 m are displayed using color-coded AMA1 peptide pools. Scales for each phenotype have been equalized. *Not tested. (TIFF) [file pone.0024586.s003.tif]

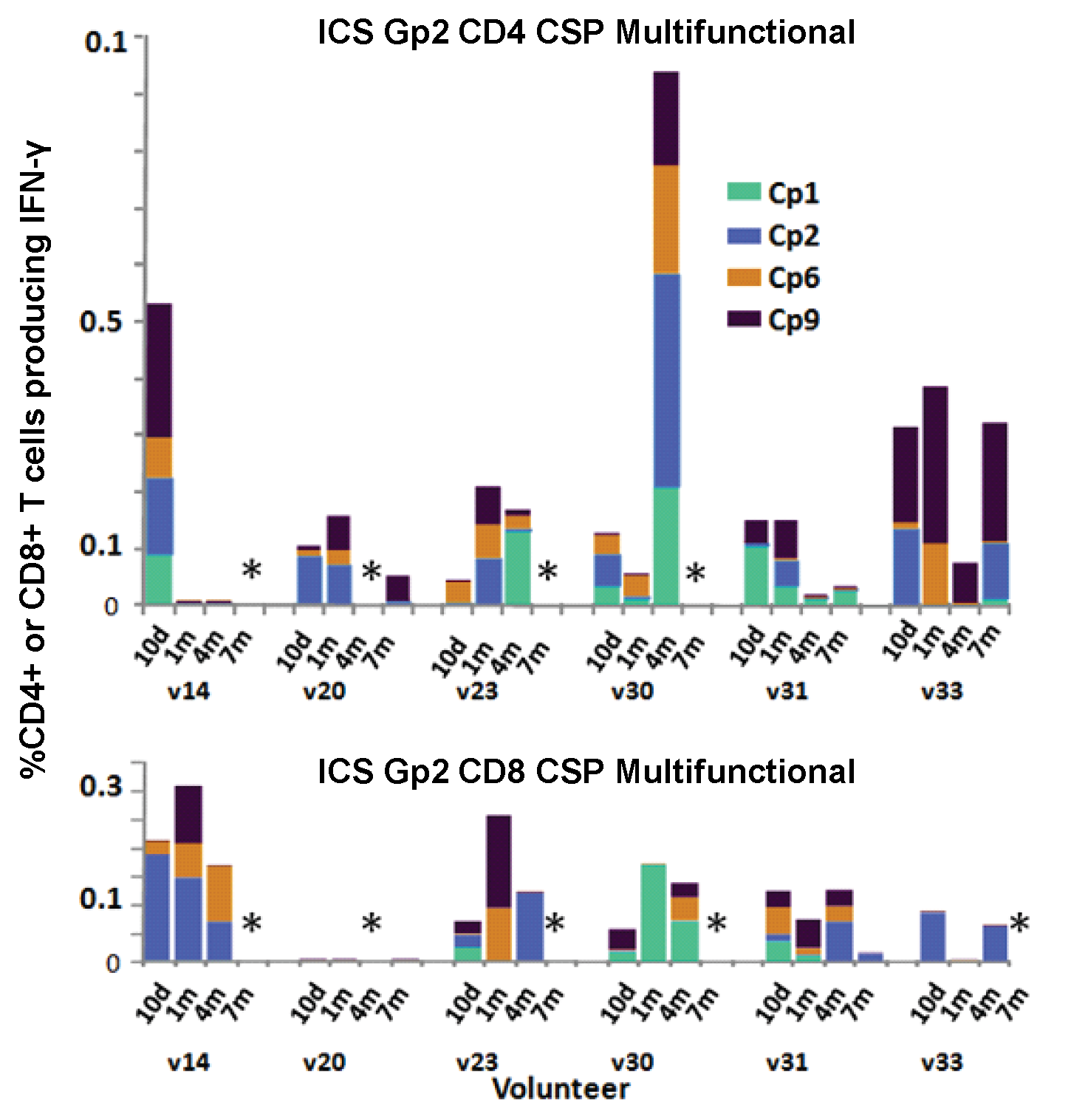

Supplement: Figure S4 — ICS multifunctional CD4+ and CD8+ T cells of serial bleeds of volunteers in Group 2 following PBMC stimulation with four dominant CSP peptide pools. The multifunctional (any two cytokines) CD4+ and CD8+ T cells activity of each volunteer at 10 d, 1, 4, and 7 m after immunization are displayed using color-coded CSP peptide pools. *Not tested. (TIF) [file pone.0024586.s004.tif]

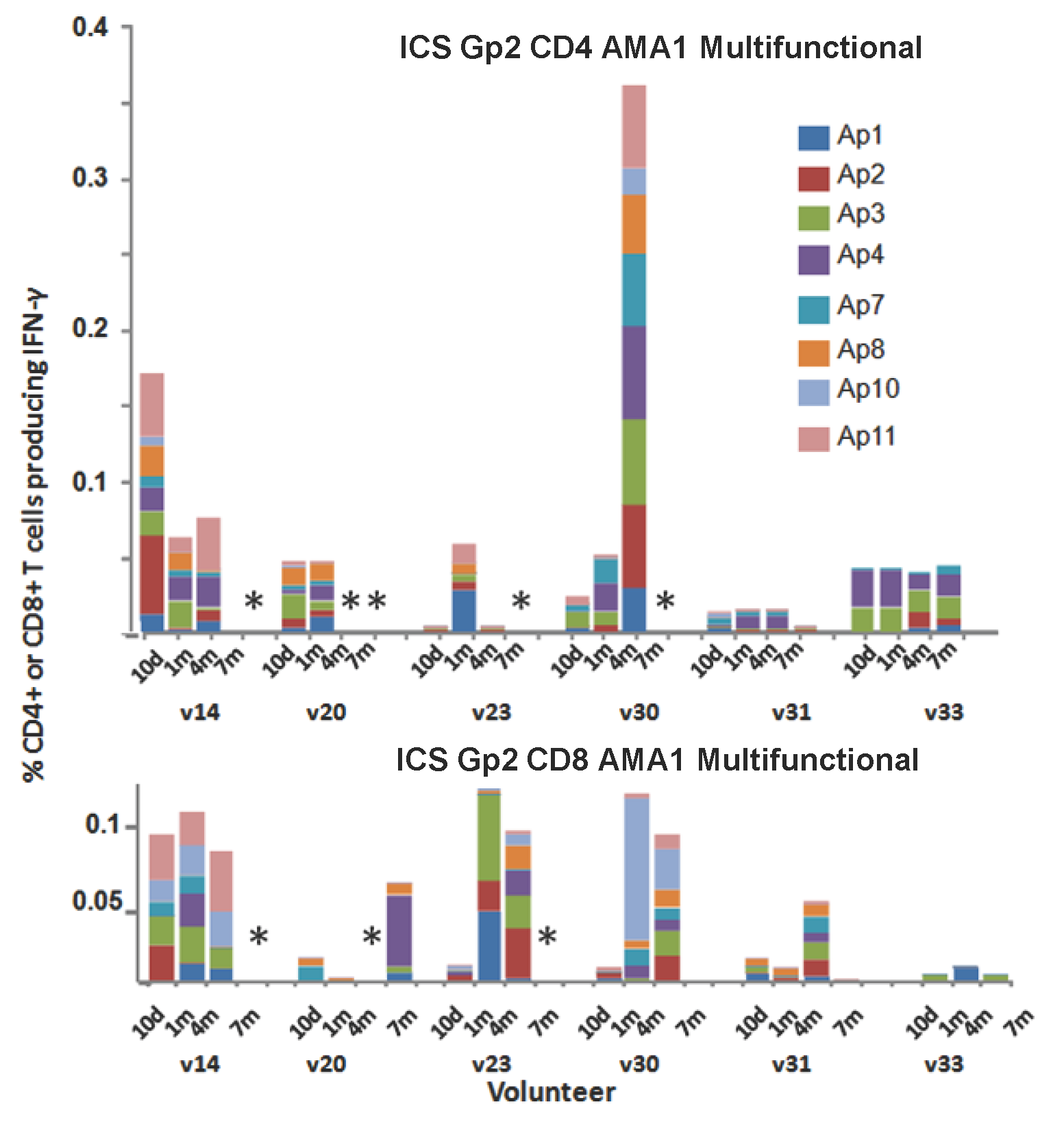

Supplement: Figure S5 — Multifunctional CD4+ and CD8+ T cells of serial bleeds of volunteers in Group 2 following PBMC stimulation with eight dominant AMA1 peptide pools. The multifunctional (any two cytokines) CD4+ and CD8+ T cell activities of each volunteer at 10 d, 1, 4, and 7 m after immunization are displayed using color-coded AMA1 peptide pools. *Not tested. (TIF) [file pone.0024586.s005.tif]

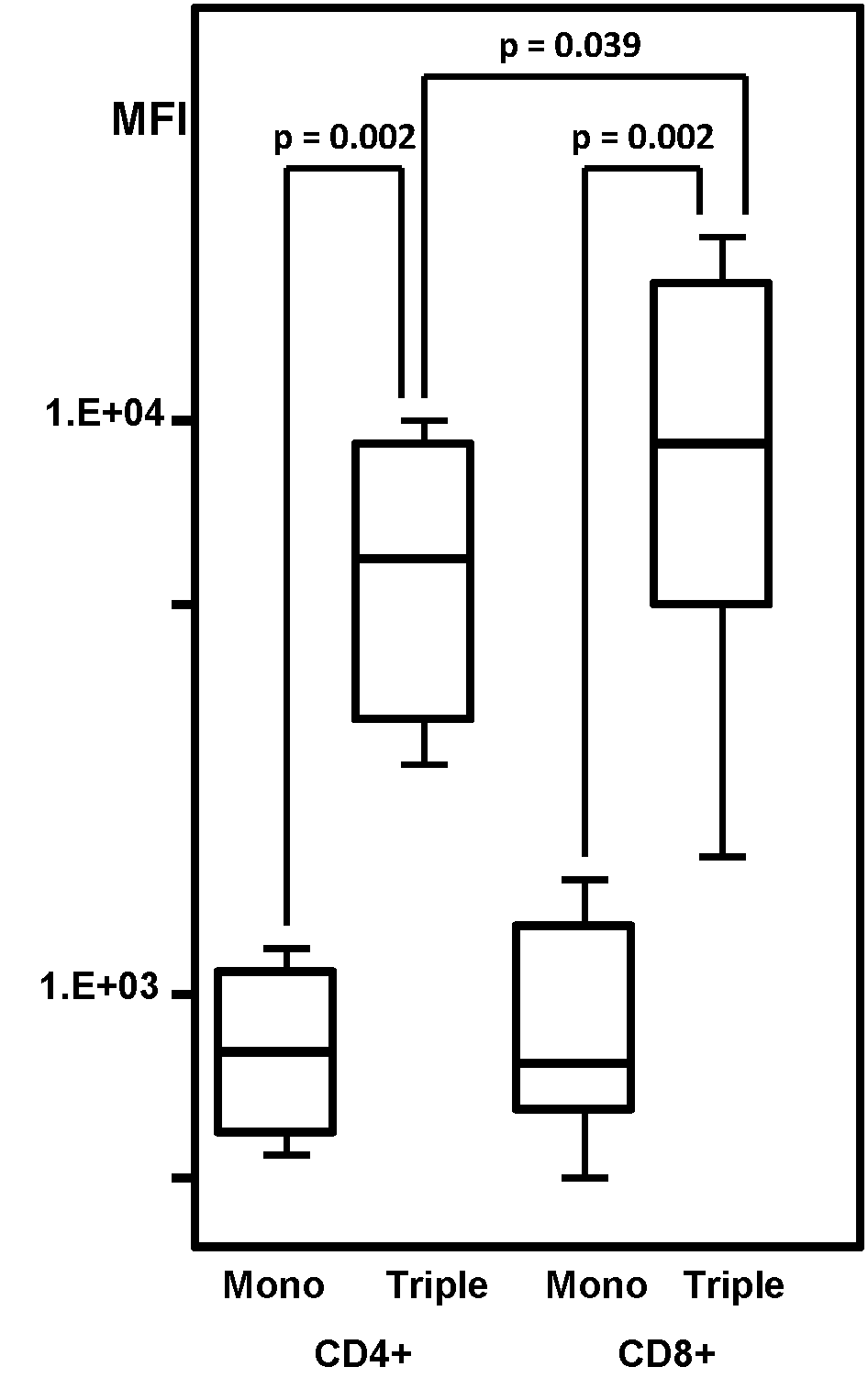

Supplement: Figure S6 — Median Fluorescence Intensity of volunteers at 1 m after immunization. The Median Fluorescent Intensity (MFI) of the IFN-γ signal for cytokine triple secretors and single secretors was measured 1 m after immunization in CD8+ (left panel) and CD4+ (right panel) T cell populations. Data from Group 1 and Group 2 and from CSP and AMA1 are combined. The boxes represent 25th to 75th percentile, the bar within the box the mean, the whiskers extend to 10th and 90th percentiles. The significance of differences between activities was calculated using a two-tailed Mann-Whitney U tests. Triple secretors appeared to show a significant 7–10-fold higher signal intensity than single secretors for CD4+ and CD8+ T cells, but CD4+ T cell triple secretors appeared similar to CD8+ triple secretors. (TIFF) [file pone.0024586.s006.tif]
