# Supplementary material for: Adenovirus 5-Vectored P. falciparum Vaccine Expressing CSP and AMA1. Part A: Safety and Immunogenicity in Seronegative Adults
Source: PLoS One. 2011 Oct 7;6(10):e24586. doi: 10.1371/journal.pone.0024586 (PMC3189181; doi:10.1371/journal.pone.0024586)
Supplement: Table S1 — Unsolicited adverse events experienced by volunteers Days 0–28. Unsolicited adverse events were recorded for 28 days following each immunization. Because of the theoretical, even if remote, possibility of reversion of the vaccine to replication competence, attention was paid to unexpected symptoms that might have reflected adenovirus infection. However, the few clinical syndromes observed in volunteers following immunization that were consistent with adenovirus infection (upper and lower respiratory infection, enteritis, urinary tract infection) bore no particular relationship to immunization in terms of timing and appeared to reflect background rates in the community. (DOC) [file pone.0024586.s007.doc]

| **Adverse event** | **Vol #** | **Grp** | **Day** | **Grade** | **Related** | **Comments** |
| --- | --- | --- | --- | --- | --- | --- |
| Axillary tenderness | V2 | 1 | 1 | 1 | Probable | Ipsilateral to immunization site |
| Axillary tenderness | V12 | 1 | 1 | 2 | Probable | Ipsilateral to immunization site |
| Axillary tenderness | V23 | 2 | 6 | 1 | Possible | Ipsilateral to immunization site |
| Pain medial arm | V23 | 2 | 6 | 1 | Probable | Ipsilateral to immunization site, no discrete dermatomal pattern, resolved by day 28 |
| Arm stiffness | V30 | 2 | 0 | 1 | Definite | Resolved the same day |
| Nasal congestion | V5 | 1 | 2 | 1 | Possible | Brief episode on awakening, cleared with blowing nose |
| Upper respiratory infection (URI) | V30 | 2 | 2 | 2 | Possible | Sinus and chest congestion, family member with similar symptoms |
| URI | V33 | 2 | 17 | 2 | Possible | Cough, sore throat, nausea day 17-21 post immunization |
| Light sensitivity, watery eyes | V14 | 2 | 0 | 1 | Possible | Occurred approximately 8 hours post immunization, resolved spontaneously within 1 hour |
| Dysuria | V20 | 2 | 1 | 2 | Possible | Viral urine culture was negative for adenovirus |
| Dry throat and remainder of unobserved symptom complex as defined in the text (Local and systemic adverse events section) | V23 | 2 | 0 | 2-3 | Probable | Occurred 8 hours post immunization, resolved spontaneously within approximately 3 hours (see text) |
| Laceration | V1 | 1 | 3 | 2 | Unrelated | Index finger |
| Furuncle | V14 | 2 | 12 | 1 | Unrelated | Right ear |
| Injury to left shoulder | V12 | 1 | 25 | 1 | Unrelated | Local physician diagnosed tendinitis or tear related to weight lifting |
| Reduced motion left shoulder | V12 | 1 | 25 | 1 | Unrelated | Ipsilateral to immunization, from day 4-25 |
