# Supplementary material for: Adenovirus 5-Vectored P. falciparum Vaccine Expressing CSP and AMA1. Part A: Safety and Immunogenicity in Seronegative Adults
Source: PLoS One. 2011 Oct 7;6(10):e24586. doi: 10.1371/journal.pone.0024586 (PMC3189181; doi:10.1371/journal.pone.0024586)
Supplement: Checklist S1 — (DOC) [file pone.0024586.s008.doc]

# CONSORT Statement 2010 - Checklist
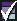


**Items to include when reporting a randomized trial**

| **Section/Topic** | **Item**  **No** | **Checklist item** |  |
| --- | --- | --- | --- |
| **Title and abstract** | 1a | Identification as a randomized trial in the title (It was sequential group assignment) | Title |
|  |  | Structured summary of trial design, methods, results, and conclusions | Background  Methodology/Principal Findings  Significance |
| **Introduction** |  |  |  |
| Background and objectives | 2a | Scientific background and explanation of rationale | Introduction |
|  | 2b | Specific objectives or hypotheses | Introduction  Objectives |
| **Methods** |  |  |  |
| Trial design | 3a | Description of trial design | Interventions |
|  | 3b | Important changes to methods after trial commencement | n/a |
| Participants | 4a | Eligibility criteria for participants | Participants |
|  | 4b | Settings and location where the data were collected | Outcomes |
| Interventions | 5 | Interventions for each group | Interventions |
| Outcomes | 6a | Primary and secondary outcome measures | Participants  Outcomes  Immunological endpoints |
|  | 6b | Any changes to trial outcomes after the trial commenced | n/a |
| Sample size | 7a | How sample size was determined | Sample size |
|  | 7b | Interim analyses and stopping guidelines | n/a |
| Randomization |  |  |  |
| Sequence determination | 8a | Method used to generate random allocation | n/a |
|  | 8b | Type of randomization | n/a |
| Allocation concealment | 9 | Method to implement random allocation | n/a |
| Implementation | 10 | Who generated the random allocation sequence, who enrolled participants, and who assigned participants to interventions | n/a |
| Blinding | 11a | If done | n/a |
|  | 11b | Description of the similarity of interventions | Interventions |
| Statistical methods | 12a | Statistical methods used to compare groups for primary and secondary outcomes | Statistical Methods |
|  | 12b | Methods for additional analyses | n/a |
| **Results** |  |  |  |
| Participant flow | 13a | No. participants. Intended treatment, analyzed | Participant Flow |
|  | 13b | Losses and exclusions after randomization and reasons | Participant Flow |
| Recruitment | 14a | Dates defining the periods of recruitment and follow-up | Participant Flow |
|  | 14b | Why the trial ended or was stopped | n/a |
| Baseline data | 15 | Baseline demographics and clinical characteristics for each group | Participant Flow |
| Numbers analyzed | 16 | No of participants in each analysis and whether this used original assigned groups | Participant Flow |
| Outcomes and estimation | 17a | Results for each group, estimated effect size and precision (95% confidence interval | Local and systemic adverse events  Unsolicited adverse events  Laboratory adverse events  Immunogenicity |
|  | 17b | Binary outcomes, absolute and relative effect sizes | n/a |
| Ancillary analyses | 18 | Results of any other analyses including subgroup analyses and adjusted analyses, distinguishing pre-specified from exploratory | Immunogenicity |
| Harms | 19 | All important harms or unintended effects in each group | Local and systemic adverse events  Unsolicited adverse events  Laboratory adverse events |
| **Discussion** |  |  |  |
| Limitations | 20 | Trial limitations, potential bias, imprecision, and multiplicity of analyses | Limitations |
| Generalizability | 21 | Generalizability (external validity, applicability) | Generalizability |
| Interpretation | 22 | Interpretation consistent with results, balancing benefits and harms, and considering other relevant evidence | Interpretation |
| **Other information** |  |  |  |
| Registration | 23 | Registration number and name of trial registry | Trial Registration |
| Protocol | 24 | Where the full trial protocol can be accessed, if available | Supplement. |
| Funding | 25 | Sources of funding and other support (such as supply of drugs), role of funders | Financial Disclosure |
