# Supplementary material for: Adenovirus 5-Vectored P. falciparum Vaccine Expressing CSP and AMA1. Part A: Safety and Immunogenicity in Seronegative Adults
Source: PLoS One. 2011 Oct 7;6(10):e24586. doi: 10.1371/journal.pone.0024586 (PMC3189181; doi:10.1371/journal.pone.0024586)
Supplement: Protocol S1 — Clinical protocol for NMRC-M3V = Ad-PfCA Vaccine. (DOC) [file pone.0024586.s009.doc]

**STUDY TITLE: A Two Part Clinical Trial Assessing the Safety, Tolerability,**

**Immunogenicity and Protective Efficacy of NMRC-M3V-Ad-PfCA, a Multivalent, Adenovirus-Vectored *Plasmodium falciparum* Malaria Vaccine, in Healthy, Malaria-Naïve Adults**

**Clinical Study Protocol**

Short Study Title: **NMRC-M3V-Ad-PfCA** Vaccine -Clinical Trial 1

Principal Investigator: Cindy Tamminga, MD, MPH

Commander, Medical Corps, US Navy

Principal Investigator, Malaria Department

Naval Medical Research Center

503 Robert Grant Avenue, 3W34

Silver Spring, MD 20910-7500

301-319-7688 (NMRC)

301-295-0007 (NMRC CTC)

cindy.tamminga@nmrc.navy.mil

Sponsor: The Surgeon General

U.S. Army

5109 Leesburg Pike, Suite 672

Falls Church, Virginia 22041-3258

Point of contact:

Robert E. Miller, Ph.D., RAC

Director, Division of Regulated Activities

and Compliance

OTSG Sponsor's Representative

U. S. Army Medical Materiel Development Activity

1430 Veterans Drive Fort Detrick, MD  21702

Telephone: 301-619-0042

Fax: 301-619-0197

NMRC Work Unit: 6000.RAD1F.A0309

RESTRICTED ACCESS

This document is a communication of NMRC that has Restricted Access. Receipt of this document constitutes agreement by the recipient that no unpublished information contained herein shall be published or disclosed without prior written approval from NMRC, except that this document may be disclosed to the appropriate IRBs and review boards under the condition that they do not disclose information contained herein.

# Glossary of Abbreviations

| AAV | | Adeno Associated Virus |
| --- | --- | --- |
| Ad | | Adenovirus |
| AE | | Adverse Event |
| Ag | | Antigen |
| ALT | | Alanine aminotransferase |
| AMA1 | | Apical Membrane Antigen 1 |
| ANA | | Anti-nuclear antibody |
| AST | | Aspartate aminotransferase |
| BGH | | Bovine Growth Hormone |
| BUMED | | Navy Bureau of Medicine and Surgery |
| BUN | | Blood Urea Nitrogen |
| CBER | | Center for Biologics Evaluation and Research |
| CBC | | Complete Blood Count |
| CDMRP | | Congressionally Directed Medical Research Program |
| CFR | | Code of Federal Regulations |
| CI | | Confidence Interval |
| CMC | | Chemistry, Manufacturing and Control |
| CMI | | Cell Mediated Immunity |
| CMO | | Contract Manufacturing Organization |
| CMP | | Complete Metabolic Panel |
| CMV | | Cytomegalovirus |
| CPE | | Cytopathic effect |
| CRO | | Contract Research Organization |
| CRF | | Case Report Form |
| CSP | | Circumsporozoite protein |
| CSS | | Clinical Seed Stock |
| CTM | | Clinical Trial Material |
| CTL | | Cytotoxic T lymphocyte |
| DAIDS | | Division of Acquired Immunodeficiency Syndrome |
| DMF | | Drug Master File |
| DNA | | Deoxyribonucleic acid |
| EKG/ECG | | Electrocardiogram |
| ELISA | | Enzyme linked immunosorbent assay |
| ELIspot | | Enzyme linked immunospot assay |
| FACS | | Fluorescence-Activated Cell Sorter |
| FDA | | Food and Drug Administration |
| FFU | | Focus Forming Unit |
| FFB | | Final Formulation Buffer |
| F/U | | Follow-up |
| FWA | | Federal-Wide Assurance |
| GCP | | Good Clinical Practice |
| GIA | Growth Inhibition Assay | |
| GLP | Good Laboratory Practices | |
| GMP | | Good Manufacturing Practices |
| GMT | | Geometric Mean Titer |
| GOT | | Glutamic oxaloacetic transaminase |
| GV | | GenVec |
| β-HCG | | Beta-Human Choriogonadotropin |
| HIV | | Human Immunodeficiency Virus |
| HbsAg, HbSAg | | Hepatitis B surface antigen |
| HLA | | Human leukocyte antigen |
| Hpf | | high power field |
| HSRRB | | Human Subjects Research Review Board |
| IAW | | in accordance with |
| IB | | Investigator’s Brochure |
| ICH | | International Conference on Harmonization |
| ICS | | Intracellular Cytokine Staining |
| ID | | Infectious Dose |
| IFA(T) | | Indirect fluorescent antibody (test) |
| IFN-γ, IFN-gamma, IFN-g | | Interferon gamma |
| IM | | Intramuscular |
| Imm | | Immunization |
| IND | | Investigational New Drug |
| IRB(s) | | Institutional Review Board(s) |
| Irr-spz | | Irradiated Sporozoite |
| ISM | | Independent Safety Monitor |
| IU | | International units |
| LAL | | Limulus Amebocyte Lysate |
| LN | | Lot Number |
| LSA1 | | Liver Stage Antigen 1 |
| LDH | | Lactate dehydrogenase |
| ml, mL | | Milliliter |
| M3V | | Multi-Stage, Multi-Antigen, Malaria Vaccine |
| MIDRP | | Military Infectious Disease Research Program |
| MF | | Master File |
| Mmol/l | | Millimole per litre |
| MSP1_42_ | | Merozoite Surface Protein 1 42k kDa C terminus |
| MV | | Malaria Vaccine |
| MVB | | Master Viral Bank |
| NHP | | Non-Human Primate |
| NIH | | National Institutes of Health |
| NMRC | | Naval Medical Research Center |
| NMRC-MV-Ad-PfC | | NMRC - Malaria Vaccine - Adenovectored - expressing *P. falciparum* Circumsporozoite Protein (3D7 strain) |
| NMRC-MV-Ad-PfA | | NMRC - Malaria Vaccine - Adenovectored – expressing *P. falciparum* Apical Membrane Antigen 1 (3D7 strain) |
| NMRC-M3V-Ad-PfCA | | NMRC - Multi-Antigen Multi-stage, Malaria Vaccine – Adenovectored – expressing *P. falciparum* Circumsporozoite Protein and Apical Membrane Antigen 1 |
| NNMC | | National Naval Medical Center |
| NS | | Normal Saline |
| OD | | Optical Density |
| O(H)RP | | Office for (Human) Research Protections |
| ORM | | Office of Research Management |
| ORF | | Open Reading Frame |
| OTSG | | Office of The Surgeon General |
| PBMC | | Peripheral Blood Mononuclear Cells |
| *Pf, P. falciparum* | | *Plasmodium falciparum* |
| Pfu, pfu | | Plaque forming units |
| PI | | Principal Investigator |
| *Pk* | | *Plasmodium knowlesi* |
| pu | | Particle Units |
| *Py, P. yoelii* | | *Plasmodium yoelii* |
| QA | | Quality Assurance |
| QC | | Quality Control |
| QRT-PCR | | Quantitative Real-Time Polymerase Chain Reaction |
| RCA | | Replication competent adenovirus |
| RNA | | Ribonucleic acid |
| SAE | | Serious Adverse Event |
| SECNAV | | Secretary of the Navy |
| SFC | | Spot Forming Cell |
| SHIV | | Simian Human Immunodeficiency Virus |
| SIV | | Simian Immunodeficiency Virus |
| SMC | | Safety Monitoring Committee |
| SOP | | Standard Operating Procedure |
| Spp | | Species |
| SRC | | Scientific Review Committee |
| SSP | | Study Specific Procedure |
| SSP2 | | Sporozoite Surface Protein 2 |
| SWFI | | Sterile Water For Injection |
| TBD | | To Be Determined |
| TIS1 | | Transcriptionally Inert Spacer 1 |
| USAID | | US Agency for International Development |
| USAMMDA | | US Army Medical Material Development Activity |
| USAMRAA | | US Army Medical Research Acquisition Activity |
| USAMRMC | | US Army Medical Research & Materiel Command |
| USUHS | | Uniformed Services University of the Health Sciences |
| VDP | | Vaccine Drug Product |
| VRC | | Vaccine Research Center |
| VSS | | Vector Seed Stock |
| VV | | Vaccinia Virus |
| WCB | | Working Cell Bank |
| WRAIR | | Walter Reed Army Institute of Research |
| μg | | Microgram |

# Clinical Protocol Synopsis

| **CLINICAL INVESTIGATIONAL PRODUCT** | **NMRC-M3V-Ad-PfCA** Vaccine | | |
| --- | --- | --- | --- |
| **PROTOCOL TITLE [No.]** | **A Two Part Clinical Trial Assessing the Safety, Tolerability, Immunogenicity and Protective Efficacy of NMRC-M3V-Ad-PfCA, a Multivalent, Adenovirus-Vectored *Plasmodium falciparum* Malaria Vaccine, in Healthy, Malaria-Naïve Adults** | | |
|  | Clinical Phase: **1 / 2a** | IND Number: BB-IND-13003 | |
| **SPONSOR COORDINATING OFFICE** | US Army Medical Research and Materiel Command (USAMRMC), Regulatory Affairs  USAMMDA  1430 Veterans Drive, Ft. Detrick, MD  21702-9232  301-619-0317; Fax:  301-619-0197 | | |
| **VACCINE MANUFACTURER** | GenVec Inc.: developed in Gaithersburg, MD and manufactured in Charlestown, Massachusetts | | |
| **PRINCIPAL**  **INVESTIGATOR** | Cindy Tamminga, MD, MPH  CDR, MC, USN  Infectious Diseases Directorate/Malaria Department  Naval Medical Research Center  503 Robert Grant Avenue  Silver Spring, MD 20910 | | |
| **ASSOCIATE**  **INVESTIGATORS** | Judith Epstein, MD CDR, MC, USN  Ilin Chuang, MD, MPH CDR, MC, USN  Thomas L. Richie, MD, PhD CAPT, MC, USN  Martha Sedegah, PhD  Christian F. Ockenhouse, MD, PhD, COL, MC, USA  Michele Spring, MD, MSPH | | |
| **MEDICAL MONITOR** | Daniel Freilich, MD CAPT, MC, USN  Director  CCC/TRMD/Hematomimetics Program  Naval Medical Research Center  503 Robert Grant Avenue  Silver Spring, MD 20910  Tel: (301) 319-4669 | |  |
| **RESEARCH ASSOCIATES** | Jose Mendoza-Silveiras, MD Keith Limbach, PhD Victoria Steinbeiss, BSN, RN  Noelle Patterson, MS Kathryn Smith, MA  Lorraine Soisson, PhD Santina Maiolatesi, BA, CIP  Frank Williams, MS, RPh CDR, MSC, USN Charlotte Fedders, BSN, RN, CCRP  Maria Sharina T. Reyes, MD | | |
| **STUDY SITE** | NMRC Malaria Program Clinical Trials Center (NNMC Building 141) Bethesda, MD.  Challenges will be conducted at the Walter Reed Army Institute of Research Insectary (WRAIR/NMRC Building 503) Silver Spring, MD. Blood draws for volunteer convenience (e.g. because they are stationed at NMRC/WRAIR) or associated with the malaria challenge will also be done at NMRC/WRAIR in an approved room for such purpose (Rm 1N56; WRAIR Transfusion Medicine Lab). | | |
| **LABORATORIES** | - GeneLogic Inc, Gaithersburg, MD (Contract Research Organization) – Nonclinical safety and toxicology testing - NMRC Malaria Department – Nonclinical immunogenicity testing; clinical trial immunology, parasitology - NNMC - clinical laboratories for screening and safety labs | | |
| **STUDY DURATION** | **Duration for each participant:** Each immunized volunteer will actively participate for approximately one year. The extended safety follow-up will last for approximately four additional years beyond active participation. Infectivity control volunteers will participate for approximately12 months.  **Duration of study:** The duration of screening and active clinical follow-up for the entire study will last approximately one year. Periodic extended safety follow-up will continue an additional four years. | | |

| **STUDY OBJECTIVES** | **Part A Dose-escalation**  Primary objective: Assess the safety and tolerability of NMRC-M3V-Ad-PfCA, in a  dose-escalation design, in healthy, malaria-naïve adults.  Secondary objective: Assess the immunogenicity of NMRC-M3V-Ad-PfCA in healthy, malaria-  naïve adults.  **Part B Regimen-comparison**  (While all regimens plan to be assessed they will be priotiorized in case full recruitment goals are not met. See below)  Primary objectives:   - Assess the safety and tolerability of the two components individually (NMRC-MV-Ad-PfC, NMRC-MV-Ad-PfA) and when combined (NMRC-M3V-Ad-PfCA) in a two dose regimen.. - Assess the protective efficacy against sporozoite challenge (*Pf*, 3D7 strain) of the two components individually (NMRC-MV-Ad-PfC, NMRC-MV-Ad-PfA) and when combined (NMRC-M3V-Ad-PfCA) in a two dose regimen.   Secondary objectives:   - Assess immunogenicity of the two components individually (NMRC-MV-Ad-PfC, NMRC-MV-Ad-PfA) and when combined. (NMRC-M3V-Ad-PfCA) in a two dose regimen. |
| --- | --- |
| **STUDY DESIGN** | This is a two-part, open-label study with sequential group assignment. The study will be conducted in two sequential phases, part A and Part B.  Part A- A dose escalation of NMRC-M3V-AdPfCA (2 antigen combination) in 12 volunteers: Two dose groups (2x10^10^ pu and 1x10^11^pu) of six volunteers each, will receive single IM injections with the injections in the two groups staggered by four weeks in order to assess the safety and tolerability of the vaccine and define the dose to be used in Part B (anticipated to be 2 x 10^10^ pu when both components are used; 1 x 10^10^  pu of each component), administered IM.  Part B: A regimen comparison of NMRC-M3V-AdPfCA and of the individual components (NMRC-MV-Ad-PfC and NMRC-MV-Ad-PfA) (dose TBD by Part A) in a two dose regimen in 12-60 volunteers (depending on recruitment thresholds):  A total of 10-20 volunteers for each regimen (minimum of 12 if only 1 group enrolled) will receive two IM injections of either combination vaccine or one of the individual components as detailed in the table below. Since the number of volunteers would be large in this scenario and the immunization and challenge schedule is complex, each dose regimen group will be split in half with two cohorts for each group. One set of cohorts (i.e. approximately 5-10 from each group) will be immunized and challenged as a group at up to a 3 week stagger from the other set of cohorts. The Infectivity Control group participating for the purpose of sporozoite challenge only will be split similarly (eg. 6/6) to accompany each of the cohort sets for challenge. However the number of volunteers and/or the regimens to be tested will be modified as outlined in the body of the protocol below if certain recruitment thresholds are not met. Safety, tolerability, immunogenicity and protective efficacy (as measured by sporozoite challenge) will be assessed. |
| ***** Depending on the rate of recruitment, there will be a prioritization for filling enrollment of the groups in Part B **(see Section 8.1) Assuming full recruitment each group will be split in half with two cohorts for each dose regimen group. One set of cohorts (i.e. approximately 8-10 from each group) will be immunized and challenged at up to a 3 week stagger from the other set of cohorts. The Infectivity Controls will be split similarly to accompany each of the cohort sets for challenge with n = 6 per challenge. Challenge will be 2-4 weeks after last immunization.**   | **Part A DOSE-ESCALATION** | | | | | | | | | | --- | --- | --- | --- | --- | --- | --- | --- | --- | | **Group** | **Test Article** | **Volunteersper group** | **Week 0** | **Week 4** | **Weeks 8-12** | | | | | 1 | NMRC-M3V-Ad-PfCA (2 Ag combination) | 6 | 2x10^10^ pu |  | Safety Monitoring Committee (SMC) Review of Safety Data  FDA Review of Safety Data | | | | | 2 | NMRC-M3V-Ad-PfCA (2 Ag combination) | 6 |  | 1x10^11^ pu | | **Part B REGIMEN-COMPARISON (doses tentative, depend on Part A)** | | | | | | | | | |  | **Test Article** | **Volunteers per group*** | **Week 16** |  | **Week 32** | **Week 34-36** | **Week 36-37** | **Week 38-48** | | 3 | NMRC-M3V-Ad-PfCA (2 Ag combination) | 20 | 2x10^10^ pu |  | 2x10^10^ pu | sporozoite challenge | Daily smears, overnight stays | Final clinical visit/ begin follow-up phase | | 4 | NMRC-MV-Ad-PfC (single Ag) | 20 | 1x10^10^ pu |  | 1x10^10^ pu | sporozoite challenge | Daily smears, overnight stays | Final clinical visit/ begin follow-up phase | | 5 | NMRC-MV-Ad-PfA (single Ag) | 20 | 1x10^10^ pu |  | 1x10^10^ pu | sporozoite challenge | Daily smears, overnight stays | Final clinical visit/ begin follow-up phase | |  | None (Infectivity Controls) | 12 |  |  |  | sporozoite challenge | Daily smears, overnight stays | Final clinical visit | | |
| **STUDY ENDPOINTS** | Safety and tolerability results will be reviewed by the Safety Monitoring Committee (SMC) and a written report will be submitted to the Sponsor, U.S. Army Medical Research and Materiel Command (USAMRMC) Office of Research Protections (ORP), relevant IRBs and FDA. Any comments and correspondence by the FDA regarding the transition from Part A to Part B will also be submitted to the same parties prior to beginning Part B. Immunogenicity assessment from Part A is not a prerequisite for continuation to Part B but will be submitted for review prior to challenge of Group B per FDA recommendations in the pre- IND meeting of April 14, 2005.  **Part A**:  Primary endpoints:   - Documentation of occurrence, severity and duration of vaccine-related solicited symptoms over a 14-day follow-up period (day of vaccination and 13 subsequent days). - Documentation of occurrence, severity and duration of vaccine-related unsolicited symptoms, abnormal physical findings and abnormal laboratory values over a 30-day follow-up period (day of immunization and 29 subsequent days). - Documentation of occurrence, severity and duration of vaccine-related serious adverse events as defined IAW 21 CFR 312.32(a) during the one year active study period or the four year period of extended safety follow-up.   Secondary endpoints:   - CSP: Analysis of IFN-γ ELIspot against synthetic peptides derived from PfCSP using PBMCs collected at screening/pre-immunization, 10 & 28 days post immunization. - AMA1: Analysis of ELISA (against recombinant PfAMA1 protein capture antigen) for sera/plasma collected at screening/pre-immunization, 10 & 28 days post immunization.   Tertiary endpoints:   - CSP: Analysis of ELISA (against recombinant CSP protein capture antigen) for sera/plasma collected at screening/pre-immunization, 10 & 28 days post immunization. - AMA1: Analysis of IFN-γ ELIspot against synthetic peptides derived from PfAMA1 and/or recombinant PfAMA1 protein using PBMCs collected at screening/pre-immunization, 10 & 28 days post immunization. - Analysis of growth inhibition assay (GIA) at screening/pre-immunization, 10 & 28 days post immunization. - Analysis of anti-sporozoite immunofluorescence assay (IFA) titers at screening/pre-immunization, and 10 & 28 days post immunization.   **Part B**:  Primary endpoints:   - Documentation of occurrence, severity and duration of solicited symptoms over a 14-day follow-up period (day of vaccination and 13 subsequent days). - Documentation of occurrence, severity and duration of unsolicited symptoms, abnormal physical findings and abnormal laboratory values over a 30 day follow-up period (day of immunization and 29 subsequent days). - Documentation of occurrence, severity and duration of serious adverse events as defined IAW 21 CFR 312.32(a) during the one year active study period, or in the following four year extended safety follow-up period. - Determination of vaccine efficacy (development of parasitemia and time to development of parasitemia) after sporozoite challenge two to four weeks post-final immunization.   Secondary endpoints:   - CSP: Analysis of IFN-γ ELIspot against synthetic peptides derived from PfCSP using PBMCs collected at screening/pre-immunization, 28 days post first immunization, prior to second immunization day of challenge (before challenge) and 28 days post challenge followed by collections at 4 ,6,and 12 months post second immunization. - AMA1: Analysis of ELISA (against recombinant PfAMA1 protein capture antigen) for sera/plasma collected at screening/pre-immunization, 28 days post first immunization, prior to second immunization, day of challenge (before challenge) and 28 days post challenge.   Tertiary endpoints:   - CSP: Analysis of ELISA (against rec. CSP protein capture antigen) for sera/plasma collected at screening/pre-immunization, 28 days post first immunization, prior to second immunization, day of challenge (before challenge) and 28 days post challenge. - AMA1: Analysis of IFN-γ ELIspot against synthetic peptides derived from PfAMA1 and/or recombinant PfAMA1 protein using PBMCs collected at screening/pre-immunization, 28 days post first immunization, prior to second immunization, day of challenge (before challenge) and 28 days post challenge. - Analysis of GIA at screening/pre-immunization, 28 days post first immunization, day of challenge (before challenge) and 28 days post challenge. Analysis of anti-sporozoite immunofluorescence assay (IFA) titers at screening/pre-immunization, 28 days post first immunization, prior to second immunization,day of challenge (before challenge) and 28 days post challenge. |
| ELIGIBILITY CRITERIA | Inclusion Criteria:   - Between the ages of 18-50 (inclusive) - Negative results of HIV ELISA, HbSAg, anti-HCV antibody, and no other clinically significant abnormal laboratory results from screening. - Adenovirus serotype 5 (Ad5) titer <1:500 (Part A Only). - Able to provide written informed consent. - Complete an Assessment of Understanding and verbalize an understanding of any questions answered incorrectly. - In good general health without clinically significant medical history or physical exam abnormalities at screening. - Willing to continue immunogenicity and clinical follow-ups for one year and telephone or mail (electronic/U.S. Postal) contact as long term safety monitoring provision for an additional four years (totaling five years of participation; immunized volunteers only). - Male and female participants being immunized and female participants being challenged agree to use effective means of birth control (an FDA approved contraceptive, abstinence) between screening and 60 days following last clinical study visit or able to provide evidence of no reproductive capability.   Exclusion Criteria:   - Plan to participate (or have participated in the last 30 days) in any other research study including an investigational drug or device during active clinical trials and follow-up. - History of malaria infection, travel to a malaria endemic region within 2 years prior to first immunization, history of long term residence (> 5 years) in area known to have significant transmission of *P. falciparum,* or receipt of a candidate malaria vaccine containing either the CSP or AMA1 antigens. - Significant cardiovascular, hepatic, renal, hematologic, or immunologic abnormality either by history or laboratory examination. Includes bleeding or seizure disorders. - Determineded, using a non-invasive cardiac risk assessment tool, to have a cardiovascular risk profile that might place them at increased risk if they should develop malaria as a result of the study. (Part B only) - A positive result on HIV testing at screening. - A positive result on Hepatitis B or C testing at screening. - An Adenovirus serotype 5 titer > 1:500 (Part A Only). - Splenectomy. - Use of immunosuppressive drugs (excluding nasal steroids or topical steroids) within thirty days of first scheduled immunization (Trial Day 0)/challenge. - Volunteers who have received immunizations (live or killed) within thirty days of either the first or second scheduled immunization. Volunteers who require immunizations for travel or other purposes during the immunization phase of the trial. - Volunteers receiving blood products within 120 days of immunization/challenge. - Serious Adverse Reaction to other vaccines (such as hives, anaphylaxis, respiratory difficulty, angioedema or abdominal pain [excluding abdominal pain caused by oral vaccines such as oral typhoid vaccine]). - Any other finding which would increase the risk of having an adverse outcome during treatment should the volunteer develop malaria as a result of the study.   Additional Criteria for Females:   - Pregnant or breast-feeding females or those planning to become pregnant within the next year. |
| **VACCINATION REGIMEN/DOSING** | Antigens (vialed separately), will be combined into a third vial according to SOP at the time of administration (for the two antigen vaccine), and injected IM at one site in the deltoid muscle (same arm to be used for both injections for volunteers who receive two injections).  **Part A**  Group 1: one dose (low) (combination, two antigen vaccine), 1 x 10^10^ pu each  construct, 2 x 10^10^ pu total dose  Group 2: one dose (standard) (combination, two antigen vaccine), 5 x 10^10^ pu  each construct, 1 x 10^11^ pu total dose (five-fold increase)  **Part B** The doses provided for Part B are based on the safety and available immunogenicity data from Part A. The higher dose, although found in Part A to demonstrate an acceptable safety profile, will only be used if compelling immongenicty data become available to warrant the use of the higher dose..  Group 3: two doses, with 16 week interval (combination, two antigen vaccine), anticipated dose  1 x10^10^ each per construct, 2 x 10^10^  pu total per dose  Group 4: two doses, with 16 week interval (single antigen vaccine; CSP), anticipated dose 1 x 10^10^  pu per construct, 1 x 10^10^ pu total dose  Group 5: two doses, with 16 week interval (single antigen vaccine; AMA1), anticipated dose 1 x 10^10^ pu per construct, 1 x 10^10^ pu total dose |
| **ASSESSMENT/**  **ANALYSIS** | **Part A** of this trial is to provide assurance that the vaccine is safe and well tolerated. This will be evaluated by review of the frequency and severity of adverse events. For example, if none of these 12 volunteers experience severe or serious vaccine related adverse events, then the likelihood that the true rate of severe or serious vaccine related adverse events in the general population is less than 20% will be 91%. Such findings will allow us to define a dose that appears to be safe and well tolerated (at a certain level of confidence for a given threshold, such as 91% confidence for a 20% threshold) and that may be used in Part B. A review by the SMC will provide recommendations regarding continued testing of the vaccine in Part B, including recommendation on the dose to be utilized. A review of the safety and tolerability results from Part A, starting from the day of immunization of Group 1 until 2 weeks after immunizing Group 2, will be conducted by the SMC and a summary report submitted to the Sponsor, USAMRMC ORP, relevant IRBs and FDA for review prior to immunization in Part B. Any comments and correspondence by the FDA regarding the transition from Part A to Part B will also be submitted to the same parties prior to beginning Part B. Immunogenicity assessment from Part A is not a prerequisite for continuation to Part B but will be submitted for review as well, prior to challenge of Group B, per FDA recommendations.  **Part B** of this trial will add additional volunteers and the level of confidence in the safety of the vaccine will increase (as long as no severe or serious vaccine related adverse events are observed). For example, with an n of 24-72 (total number of vaccinees in the trial from both Part A & B without and with Groups 3 &5), the confidence levels that the true rate of severe or serious vaccine related adverse events in the general population is less 20% is 99.18% to 99.99% (assuming that none occur in the trial). This confidence level would provide sufficient assurance of safety to justify the testing of larger numbers of volunteers in subsequent trials.  The sample size for Part B is also powered sufficiently to show statistically significant differences between regimens in immunogenicity and in protective efficacy. |

# Clinical Trial Time and Event Schedules

## Part A: Dose Finding

### Note: Unless otherwise indicated each time point has a window of +/- 2 days to account for volunteer scheduling, holidays or unforeseen events, however every attempt will be made to target the exact day. Also, timepoints after the 28 day post immunization visit have a window of +/- 7 days.

| **Schedule of Events by Week**  **PART A** |  |  |  |  |  | **Week 1** |  | **Week 2** | **Week 3** | **Week 4** |  |  | **Week 5** |  | **Week 6** | **Week 8** | **Week 9-12** | **Week 12-260** |
| --- | --- | --- | --- | --- | --- | --- | --- | --- | --- | --- | --- | --- | --- | --- | --- | --- | --- | --- |
| **Study Day** | **-30 to -14 (+/-7)** | **-7** | **0** | **1** | **2** | **7** | **10** | **14** | **21** | **28** | **29** | **30** | **35** | **38** | **42** | **56** | **57-84** | Q 3 Month clinical F/U to Week 52 then Annual phone f/u until week 260 |
| **Study Event** | **Screen ^#^,*** | **Review^#^** | **Imm^#^** | **F/U ^#^** | **F/U ^#^** | **F/U ^#^** | **F/U ^#^** | **F/U ^#^** | **Rev.*** | **F/U ^#^**  **Imm *** | **F/U *** | **F/U *** | **F/U *** | **F/U *** | **F/U *** | **F/U *** | **SMC Review** |  |
| **Consent** | X |  |  |  |  |  |  |  |  |  |  |  |  |  |  |  |  |  |
| **Physical Exams:** |  |  |  |  |  |  |  |  |  |  |  |  |  |  |  |  |  |  |
| Detailed | X |  |  |  |  |  |  |  |  |  |  |  |  |  |  |  |  |  |
| Abbreviated |  | X | X |  | X | X | X | X | X | X |  | X | X | X | X | X |  | X |
| **Labs:** |  |  |  |  |  |  |  |  |  |  |  |  |  |  |  |  |  |  |
| Screen (Hepatitis B/C, HIV, HLA type, Ad5 serology ) | X |  |  |  |  |  |  |  |  |  |  |  |  |  |  |  |  |  |
| Safety (CBC, CMP, UA) | X |  | X |  | X | X |  | X |  | X |  | X | X |  | X | X |  | X |
| Beta hCG (females only) | X | X | X |  |  |  |  |  | X | X |  |  |  |  |  | X |  |  |
| Blood for ELISA, IFA, GIA, AMA, CSP assays |  | X |  |  |  |  | X |  | X | X^#^ |  |  |  | X |  | X |  | X |
| **Medical History:** |  |  |  |  |  |  |  |  |  |  |  |  |  |  |  |  |  |  |
| Comprehensive Review | X |  |  |  |  |  |  |  |  |  |  |  |  |  |  |  |  |  |
| Update/ Recent History |  | X | X | X | X | X | X | X | X | X | X | X | X | X | X | X |  | X |
| **Volunteer Diary (daily record)** |  | X | X | X | X | X |  |  | X | X* | X | X | X |  |  |  |  |  |
| **Phone Check** |  |  |  | X |  |  |  |  |  |  | X |  |  |  |  | X |  | X |
| **Record Adverse Events** |  |  | X | X | X | X | X | X |  | X | X | X | X | X | X | X |  | X |
| **#= GROUP 1 Only**  ***= GROUP 2 Only** |  |  |  |  |  |  |  |  |  |  |  |  |  |  |  |  |  |  |

## Part B: Regimen Comparison

### Note: Unless otherwise indicated each time point has a window of +/- 2 days to account for volunteer scheduling, holidays or unforeseen events, however every attempt will be made to target the exact day. Also, time points involving the collection of immunologic assays during the immunologic, challenge, and follow-up phases will have a +/- 7 day window. However, every attempt will be made to target the exact date.

###

| **Schedule of Events**  **Part B- Immunization** |  |  |  |  |  |  |  | **Week 1** | **Week 2** | **Week 4** | **Week 15** | **Week 16** |
| --- | --- | --- | --- | --- | --- | --- | --- | --- | --- | --- | --- | --- |
| **Study Day**  **(For Part B Volunteers)** | **-90 to -14 (+/-7)** | **-7** | **0** | **1** | **2** | **3** | **5** | **7** | **14** | **28** | **105** | **112** |
| **Study Event** | **Screen** | **Review** | **Imm #1** | **F/U** | **F/U** | **F/U** | **F/U** | **F/U** | **F/U** | **F/U** | **Review** | **Imm #2** |
| **Consent** | X |  |  |  |  |  |  |  |  |  |  |  |
| **Physical Exams:** |  |  |  |  |  |  |  |  |  |  |  |  |
| Detailed | X |  |  |  |  |  |  |  |  |  |  |  |
| Abbreviated |  | X | X | X | X | X | X | X | X | X | X | X |
| **Labs:** |  |  |  |  |  |  |  |  |  |  |  |  |
| Screen (Hepatitis B/C,  HIV, , HLA type, G6PD,  Ad5 serology) | X |  |  |  |  |  |  |  |  |  |  |  |
| Safety (CBC, Chemistry, UA)¶ | X |  | X |  |  |  |  |  | X |  |  | X |
| Beta hCG (females only) | X | X | X |  |  |  |  |  |  |  | X | X |
| Safety (CBC, Chemistry) |  |  |  |  | X |  |  | X |  | X |  |  |
| Safety (CBC only) |  |  |  | X |  | X | X |  |  |  |  |  |
| Blood for ELISA, IFA, GIA, AMA, CSP assays |  | X |  |  |  |  |  |  |  | X | X |  |
| ECG** |  |  |  |  |  |  |  |  |  |  |  |  |
| **Medical History:** |  |  |  |  |  |  |  |  |  |  |  |  |
| Comprehensive Review | X |  |  |  |  |  |  |  |  |  |  |  |
| Update/ Recent History |  | X | X | X | X | X | X | X | X | X | X | X |
| **Volunteer Temperature Log (daily)** |  | X | X | X | X | X | X | X |  |  | X | X |
| **Record Adverse Events** |  |  | X | X | X | X | X | X | X | X |  | X |

**TABLE CONTINUED ON FOLLOWING PAGE**

| **Schedule of Events**  **Part B- Immunization Cont’d**  **(weeks continued from Part A)** |  |  |  |  | **Week 17** | **Week 18** |
| --- | --- | --- | --- | --- | --- | --- |
| **Study Day** | **113** | **114** | **115** | **117** | **119** | **126** |
| **Study Event** | **F/U** | **F/U** | **F/U** | **F/U** | **F/U** | **F/U** |
| **Consent** |  |  |  |  |  |  |
| **Physical Exams:** |  |  |  |  |  |  |
| Detailed |  |  |  |  |  |  |
| Abbreviated | X | X | X | X | X | X |
| **Labs:** |  |  |  |  |  |  |
| Screen (Hepatitis B/C,  HIV, , HLA type, G6PD,  Ad5 serology ) |  |  |  |  |  |  |
| Safety (CBC, Chemistry, UA) |  |  |  |  |  | X |
| Safety (CBC, Chemistry) |  | X |  |  | X |  |
| Safety (CBC only) | X |  | X | X |  |  |
| Beta hCG (females only) |  |  |  |  |  |  |
| Blood for ELISA, IFA, GIA, AMA, CSP assays |  |  |  |  |  |  |
| **Medical History:** |  |  |  |  |  |  |
| Comprehensive Review |  |  |  |  |  |  |
| Update/ Recent History | X | X | X | X | X | X |
| **Volunteer Temperature Log (daily)** | X | X | X | X | X |  |
| **Record Adverse Events** | X | X | X | X | X | X |

| **Part B- Challenge**  **(weeks continued from Immunization phase)** | **Week 18-20 post imm.** | **Week 1-3 post†** | **Week 4 post**† | **Week 5-8**  **post**† | **Week 12 post†** | **Week 13-48post**† | **Week 49-261**† |
| --- | --- | --- | --- | --- | --- | --- | --- |
| **Study Day** | 126-140 | 7-21† | 22-28† | 29-56† | 84 |  |  |
| **Study Event** | **Single Challenge** | **Daily F/U** | Every Other Day F/U | Weekly F/U | Active Phase Close out  visit | 6 and 12 month F/U (relative to 2^nd^ immunization) | Annual  Phone/e-mail Follow-up |
| **Physical Exams:** |  |  |  |  |  |  |  |
| Detailed |  |  |  |  |  |  |  |
| Abbreviated | X | X | X | X | X | X |  |
| **Labs:** |  |  |  |  |  |  |  |
| Safety (CBC, Chemistry, UA)# |  | I | I | X | X | X |  |
| Blood for ELISA, IFA, GIA, AMA, CSP assays | X |  | X‡ |  | X | X |  |
| Blood Smears |  | X | X | X | X | O |  |
| Beta hCG (females only) | X |  |  |  |  |  |  |
| **Challenge** | X |  |  |  |  |  |  |
| **Medical History:** |  |  |  |  |  |  |  |
| Comprehensive Review |  |  |  |  |  |  |  |
| Update/ Recent History | X | X | X | X | X | X | X |
| **Phone/e-mail Check** |  |  |  |  |  |  | X |
| **Record Adverse Events** | X* | X* | X* | X* | X* | X | X |

** Baseline ECG to be obtained within 1 year of date of challenge

†: Weeks and days relative to challenge day.

I: If clinically indicated and a minimum of once a week if asymptomatic.

O: If clinically indicated.

X*: During this period post-challenge signs and symptoms will be considered challenge/parasitemia related and not vaccine related unless evidence suggest otherwise.

‡ This will only be done once on day 28 after challenge, NOT every other day.

#: UA will be collected only as clinically indicated throughout this post challenge phase; safety chemistry labs include Creatinine, ALT, AST

# Table of Contents

1 Glossary of Abbreviations [2](#__RefHeading___Toc131481364)

2 Clinical Protocol Synopsis [6](#__RefHeading___Toc131481365)

3 Clinical Trial Time and Event Schedules [12](#__RefHeading___Toc131481366)

3.1 Part A: Dose Finding [12](#__RefHeading___Toc131481367)

3.1.1 Note: Unless otherwise indicated each time point has a window of +/- 2 days to account for volunteer scheduling, holidays or unforeseen events, however every attempt will be made to target the exact day. [12](#__RefHeading___Toc131481368)

3.2 Part B: Regimen Comparison [13](#__RefHeading___Toc131481369)

4 Table of Contents [15](#__RefHeading___Toc131481370)

5 Introduction [18](#__RefHeading___Toc131481371)

5.1 Overview [18](#__RefHeading___Toc131481372)

5.2 The Need for a Malaria Vaccine [19](#__RefHeading___Toc131481373)

5.3 Vaccine Development Strategy- [19](#__RefHeading___Toc131481374)

5.3.1 Rationale for a Multi-Antigen, Multi-Stage, Multi-Immune Response Strategy for Malaria Vaccine Development [19](#__RefHeading___Toc131481375)

5.3.2 Rationale for Antigen Selection [21](#__RefHeading___Toc131481376)

5.3.3 Rationale for Vaccine Vector Selection – Recombinant Adenovirus serotype 5 [22](#__RefHeading___Toc131481377)

5.3.4 Rationale for Clinical Trial Design [22](#__RefHeading___Toc131481378)

*5.3.4.1* Rationale for a long interval between prime and boost [23](#__RefHeading___Toc131481379)

*5.3.4.2* Rationale for a short interval between prime and boost [25](#__RefHeading___Toc131481380)

*5.3.4.3* Rationale for the comparison of one vs two doses of vaccine [26](#__RefHeading___Toc131481381)

5.3.5 Previous Human Experience with Adenovirus-vectored Vaccines [27](#__RefHeading___Toc131481382)

5.3.6 Nonclinical experience with NMRC-M3V-Ad-PfCA [27](#__RefHeading___Toc131481383)

*5.3.6.1* Nonclinical Safety of the NMRC-M3V-Ad-PfCA Vaccine [28](#__RefHeading___Toc131481384)

*5.3.6.2* Disaster Check Immunogenicity of the Clinical Lots [29](#__RefHeading___Toc131481385)

6 Study Objectives [29](#__RefHeading___Toc131481386)

6.1 Part A- Dose-escalation [29](#__RefHeading___Toc131481387)

6.2 Part B- Regimen-comparison [29](#__RefHeading___Toc131481388)

7 Study Population [30](#__RefHeading___Toc131481389)

7.1 Subjects [30](#__RefHeading___Toc131481390)

7.2 Sample Size and Power Consideration [30](#__RefHeading___Toc131481391)

7.3 Subject Selection Criteria [31](#__RefHeading___Toc131481392)

7.3.1 Inclusion Criteria [31](#__RefHeading___Toc131481393)

7.3.2 Exclusion Criteria [31](#__RefHeading___Toc131481394)

8 Study Design [32](#__RefHeading___Toc131481395)

8.1 Summary [32](#__RefHeading___Toc131481396)

8.2 Go-No Go criteria for initiating the clinical trial [34](#__RefHeading___Toc131481397)

8.3 Go-No Go criteria for transition from Part A to Part B [34](#__RefHeading___Toc131481398)

8.4 Go-No Go criteria for transition to future clinical trials [34](#__RefHeading___Toc131481399)

9 Study Duration [35](#__RefHeading___Toc131481400)

10 Study Procedures [35](#__RefHeading___Toc131481401)

10.1 Recruitment [35](#__RefHeading___Toc131481402)

10.2 Screening Visit [35](#__RefHeading___Toc131481403)

10.3 Immunization Visits [36](#__RefHeading___Toc131481404)

10.3.1 Prior to Immunization [36](#__RefHeading___Toc131481405)

10.3.2 Immunization [37](#__RefHeading___Toc131481406)

10.3.3 Post-Immunization [37](#__RefHeading___Toc131481407)

10.4 Post Immunization Follow-up Visits [38](#__RefHeading___Toc131481408)

10.5 Compliance Ranges and Holding Rules [39](#__RefHeading___Toc131481409)

10.6 Experimental Challenge with Malaria Infected Sporozoites [39](#__RefHeading___Toc131481410)

10.6.1 Challenge procedure [40](#__RefHeading___Toc131481411)

10.6.2 Parasitemia [40](#__RefHeading___Toc131481412)

10.6.3 Management of Volunteers POST CHALLENGE (PC) [40](#__RefHeading___Toc131481413)

10.6.4 Treatment of Malaria for Infected Volunteers [42](#__RefHeading___Toc131481414)

10.7 Clinical Assessments [43](#__RefHeading___Toc131481415)

10.7.1 Physical exams [43](#__RefHeading___Toc131481416)

10.7.2 Laboratories [43](#__RefHeading___Toc131481417)

10.7.3 Volunteer Temperature Log [43](#__RefHeading___Toc131481418)

10.7.4 Long Term Follow-up [43](#__RefHeading___Toc131481419)

10.8 Specimen Collection/Processing/Storage [44](#__RefHeading___Toc131481420)

10.8.1 Blood specimens [44](#__RefHeading___Toc131481421)

10.8.2 HLA Typing [44](#__RefHeading___Toc131481422)

10.9 Concomitant Medications [44](#__RefHeading___Toc131481423)

10.10 Withdrawal / Drop-outs [44](#__RefHeading___Toc131481424)

10.11 Study Termination [44](#__RefHeading___Toc131481425)

11 Study Product [45](#__RefHeading___Toc131481426)

11.1 Dose Groups [45](#__RefHeading___Toc131481427)

11.2 Route of Administration [45](#__RefHeading___Toc131481428)

12 Adverse Experiences [45](#__RefHeading___Toc131481429)

12.1 Adverse event definition [46](#__RefHeading___Toc131481430)

12.2 Reporting Procedures for Adverse Experiences [46](#__RefHeading___Toc131481431)

12.2.1 Solicited Adverse Events [46](#__RefHeading___Toc131481432)

12.2.2 Unsolicited adverse events [47](#__RefHeading___Toc131481433)

12.3 Serious Adverse Experiences (Events) [47](#__RefHeading___Toc131481434)

12.4 Unexpected Adverse Experiences [48](#__RefHeading___Toc131481435)

12.5 Classification of Adverse Experiences [48](#__RefHeading___Toc131481436)

12.5.1 Severity/Intensity [48](#__RefHeading___Toc131481437)

12.5.2 Relationship to participation in study [54](#__RefHeading___Toc131481438)

13 Endpoints [54](#__RefHeading___Toc131481439)

13.1 Safety [55](#__RefHeading___Toc131481440)

14 Immunology Definitions [55](#__RefHeading___Toc131481441)

14.1 Humoral Responses [57](#__RefHeading___Toc131481442)

14.2 Cellular Responses [57](#__RefHeading___Toc131481443)

15 Clinical Definitions [57](#__RefHeading___Toc131481444)

15.1 Protective Efficacy [57](#__RefHeading___Toc131481445)

16 Human Subjects Protection Considerations [57](#__RefHeading___Toc131481446)

16.1 Risks of study participation [57](#__RefHeading___Toc131481447)

16.2 Precautions to Minimize Risk of Study participation [59](#__RefHeading___Toc131481448)

16.2.1 Precautions To Minimize Risks Associated With Blood Drawing [59](#__RefHeading___Toc131481449)

16.2.2 Precautions To Minimize Risks Associated With Immunization [59](#__RefHeading___Toc131481450)

16.2.3 Precautions To Minimize Risks Associated With Challenge [59](#__RefHeading___Toc131481451)

17 Data Management and Analysis [60](#__RefHeading___Toc131481452)

17.1 Data Collection and Storage [60](#__RefHeading___Toc131481453)

17.2 Statistical Analysis Plan [61](#__RefHeading___Toc131481454)

17.2.1 Hypotheses to be tested [61](#__RefHeading___Toc131481455)

17.2.2 Analysis of Safety and Tolerability [61](#__RefHeading___Toc131481456)

17.2.3 Analysis of Immunogenicity [61](#__RefHeading___Toc131481457)

17.2.4 Analysis of Efficacy [61](#__RefHeading___Toc131481458)

17.2.5 Statistical Tests [62](#__RefHeading___Toc131481459)

18 Recording and Collection of Data [62](#__RefHeading___Toc131481460)

19 Monitoring [62](#__RefHeading___Toc131481461)

20 Audit and Inspection [62](#__RefHeading___Toc131481462)

21 Modification to the Protocol [62](#__RefHeading___Toc131481463)

22 Adherence to and Changes in Protocol [63](#__RefHeading___Toc131481464)

23 Investigational Product Accountability [63](#__RefHeading___Toc131481465)

24 Retention of Records [63](#__RefHeading___Toc131481466)

25 Disclosure of Information [63](#__RefHeading___Toc131481467)

26 Medical Care for Research-Related Injuries [63](#__RefHeading___Toc131481468)

27 Obligations of the Sponsor and the Investigator [64](#__RefHeading___Toc131481469)

28 Roles and Responsibilities of Study Personnel [64](#__RefHeading___Toc131481470)

29 Protocol Review Process [64](#__RefHeading___Toc131481471)

30 Signature of the Investigator [65](#__RefHeading___Toc131481472)

31 Appendices (In separate files) [65](#__RefHeading___Toc131481473)

# Introduction

## Overview

The Naval Medical Research Center (NMRC) is developing a recombinant adenovirus-vectored vaccine for the prevention of malaria caused by the parasite *Plasmodium falciparum*. The vaccine, called NMRC-M3V-Ad-PfCA (key: NMRC + Multi-antigen Multi-stage, Malaria Vaccine + Adenovectored + *P. falciparum* CSP & AMA1 antigens), is a combination of two recombinant adenovirus-derived constructs (adenovectors), one expressing the pre-erythrocytic stage antigen circumsporozoite protein (CSP) and the other expressing the erythrocytic stage antigen Apical Membrane Antigen 1 (AMA1), both from the 3D7 strain of *P. falciparum*. The CSP and AMA1 genes are synthetic and optimized for expression in mammalian cells (codon-optimized). The vector is an attenuated, replication-deficient adenovirus derived from wildtype serotype 5 adenovirus through the deletion of several genes (missing the E1 and E4 regions, each of which is required for replication, and also missing the E3 region). The vaccine is formulated in a buffered saline solution (Final Formulation Buffer = FFB).

The NMRC-M3V-Ad-PfCA vaccine has been jointly developed by the NMRC Malaria Program and GenVec, Inc, Gaithersburg, Maryland in partnership with the US Agency for International Development (USAID). The IND for the vaccine will be sponsored by the Surgeon General for the Army. Funding has been provided by USAID, MIDRP, and the Congressionally Directed Medical Research Program (CDMRP).

This first clinical study will be a two-part, Phase 1/2a, open-label, dose-escalating trial of the NMRC-M3V-Ad-PfCA vaccine administered intramuscularly to healthy, malaria-naïve adult volunteers. All volunteers for Part A will be low-titer (< 1:500, by a luciferase-based neutralizing antibody assay; VRC, Bethesda) for adenovirus serotype 5 (Ad5) while Part B will be a mix of Ad5 serpositive and seronegative with a minimum of (n/2)-2 of low titer volunteers for each study group. In the first part of the study (dose-escalation phase, Part A), 1 x 10^10^ particle units (pu) per construct or 2 x 10^10^ pu total will be administered to six volunteers as a single dose to assess safety, and 4 weeks later, 5 x 10^10^ pu per construct or 1 x 10^11^ pu total dose (five-fold dose escalation) will be administered to six additional volunteers. In the second part of the study (regimen-comparison phase, Part B), three regimens for administration will be compared: two doses of the combined antigen vaccine (NMRC-M3V-Ad-PfCA) administered 16 weeks apart or two doses of one or the other of the individual components of the vaccine (NMRC-MV-Ad-PfC or NMRC-MV-Ad-PfA) administered 16 weeks apart. These regimens/groups will be prioritized based on recruitment thresholds set for the 90 day recruitment/screening period outlined in Section 8.1 with *P. falciparum* sporozoites in order to assess vaccine efficacy. The proposed design of the regimen-comparison phase will provide information to direct selection of an appropriate dosing regimen for subsequent studies, and will also indicate whether the two constituent antigens, when co-formulated, act synergistically, independently, or interfere with each other in the induction of antigen-specific immune responses and protective immunity.

Safety and tolerability will be measured by questioning and examining volunteers, and by periodic blood draws for hematology and chemistry tests. A Safety Monitoring Committee (SMC; See Appendix L) will review the safety data of the dose-escalation phase prior to initiation of the regimen-comparison phase. In addition, a written report by the SMC will be provided to the Sponsor, USAMRMC ORP, relevant IRBs and FDA for review prior to initiation of part B. The study has been designed to accommodate this safety review process by being sectioned into parts A (dose-escalation) and B (regimen-comparison).

Immunogenicity will be measured to provide information on the capacity of the adenovectored vaccine to induce antigen-specific immune responses in humans and on the kinetics of induction of immune responses. Antibody immunogenicity will be measured by indirect fluorescent antibody test (IFAT) against sporozoites and blood stage parasites, and by ELISA against recombinant proteins or synthetic peptides derived from the CSP and AMA1 antigens. T cell immunogenicity will be measured by cytokine ELIspot and intracellular cytokine staining (ICS) assays. T cell and antibody responses will be measured at defined time points following immunization to evaluate vaccine-induced immunogenicity as well as at a defined time points post challenge to evaluate any boosting effect due to exposure to infectious sporozoites. A report on the immunogenicity of the vaccine in Part A will be provided to the FDA prior to initiation of challenge in Part B.

Protective efficacy will be assessed by challenging volunteers enrolled in the regimen-comparison phase with *P. falciparum* sporozoites, which will be administered via the bites of five infectious mosquitoes. Efficacy will be measured by determining the frequency of parasitemia in vaccine recipients to that in non-immunized infectivity controls and by comparing days to onset of parasitemia in those volunteers not fully protected with days to onset in the controls. Immunological follow-up will continue for approximately one year from day 0 (day of first immunization) and safety follow-up for five years beginning at Day 0 (day of first immunization).

If there is protection afforded against experimental sporozoite challenge, the vaccine will undergo further Phase 1a and Phase 2a testing in the United States, followed by Phase 1b and Phase 2b testing in endemic areas.

## The Need for a Malaria Vaccine

Malaria represents a major public health problem worldwide, causing significant morbidity and mortality in immunologically naïve individuals as well as endemic populations. Transmission occurs throughout tropical Africa, Asia, Oceania, and Latin America as well as some subtropical areas. It has been reported that malaria is responsible for 300-500 million clinical cases annually, and between one and three million deaths, mostly in children[1]. Analyses suggest that the medical impact of malaria may actually have been significantly underestimated[2]), and that the enormous economic impact of malaria has never been adequately considered[3]. Additionally, malaria remains a major public health threat to non-immune travelers to areas where malaria is transmitted, including both international tourists and individuals residing in malaria-free regions of malaria-endemic countries who travel locally to areas where malaria is transmitted. Increasing drug resistance by the parasite and insecticide resistance by the vector highlight the importance of developing an effective malaria vaccine.

## Vaccine Development Strategy-

### Rationale for a Multi-Antigen, Multi-Stage, Multi-Immune Response Strategy for Malaria Vaccine Development

Pre-erythrocytic Stage Immunity: Sterile protective immunity against malaria can be induced in animals or human volunteers by immunization with *Plasmodium* sporozoites attenuated by radiation so that they can invade the host hepatocyte but cannot transform into blood stage parasites[4, 5]. This protection is effective against challenge with high doses of infectious sporozoites, is species-specific but not strain-specific, is efficacious in genetically diverse backgrounds, and is sustained. This irradiated sporozoite model represents a human model for pre-erythrocytic stage vaccine development and demonstrates its feasibility. Such a vaccine would completely prevent infection. The primary protective immune mechanism in the irradiated sporozoite-induced protection is thought to be cell mediated immune responses directed against parasite proteins expressed by irradiated sporozoites in infected hepatocytes[6-8]. The importance of antigenic targets and immune mechanisms that are active in this model is highlighted by the fact that infection-blocking immunity in humans rarely, if ever, occurs under natural conditions.

Erythrocytic Stage Immunity: In areas where malaria is endemic, there is a decrease in the incidence of *Pf* infections, the prevalence and density of parasitemia, and the morbidity and mortality associated with *Plasmodium* spp. infection with age[9]. Passive transfer of purified immunoglobulin from individuals with lifelong exposure to endemic malaria results in a marked decrease in *Pf* blood-stage parasitemia and resolution of symptoms in the recipients[10-13], suggesting that antibodies directed against antigens exposed on the surface of merozoites or infected erythrocytes, or released from apical organelles at the moment of invasion, are the critical protective mechanism. This naturally acquired immunity model represents a human model for erythrocytic stage vaccine development. Such a vaccine would prevent death and severe disease.

*Multi-Antigen, Multi-Stage, Multi-Immune Response Vaccine*

Based on these two models of protection, our rational approach to malaria vaccine development focuses on a two-tiered approach, for the development of a multi-antigen multi-stage multi-immune response malaria vaccine: (i) induction of T cell-mediated immune responses, directed against the parasite antigens expressed by irradiated sporozoites within hepatocytes (liver-stage parasite antigens), and (ii) induction of antibody responses directed against parasite proteins expressed on the surface of merozoites or infected erythrocytes or in apical organelles.

One component is designed to reproduce irradiated sporozoite-induced immunity, which is directed at liver stage infections. Induction of T cell responses against liver-stage antigens would destroy the majority of developing parasites during the five-day window of hepatic stage development. However, since each sporozoite can potentially develop into 10,000-30,000 merozoites each of which may potentially undergo a series of asexual amplifications resulting in a 10 to 20-fold increase in the numbers of parasites in the bloodstream every 48 hours, a hepatic stage vaccine must be 100% effective. Therefore, the second component is designed to reproduce naturally acquired immunity, which is directed at blood stages. Induction of antibody responses against erythrocytic stage antigens would protect against severe disease and death in individuals who experience break-through blood-stage infections. This “combined stage” approach is designed to prevent infection by killing the majority of developing parasites in the liver, and also to prevent severe disease and death should break-through blood stage infections occur.

Our hypothesis is that by reducing the numbers of parasites emerging from the liver (via T cell immune responses directed against those antigens expressed by irradiated sporozoites in hepatocytes) and priming the immune system to erythrocytic stage antigens that will be boosted by infection from natural exposure (via antibody responses directed against parasite proteins expressed on the surface of merozoites or infected erythrocytes or apical organelles), the severity and mortality of *P. falciparum* malaria will be reduced.

In the current proposal, we have elected to focus on CSP as a model pre-erythrocytic antigen (primary target of T cell responses) and AMA1 as a model erythrocytic stage antigen (primary target of antibody responses). In addition, AMA1 is also expressed during the pre-erythrocytic stages which may provide additional liver stage protection.

Studies in the *P. knowlesi* rhesus monkey model strongly support our multi-antigen, multi-stage, multi-immune response approach. In our laboratory, we have now consistently demonstrated that a four-antigen multi-stage vaccine can protect rhesus monkeys against a virulent parasite challenge. In rhesus monkeys, *P. knowlesi* is invariably fatal, and even immunization with the whole organism irradiated sporozoite vaccine does not consistently provide protection. In contrast, multiple studies have now established that priming intramuscularly (IM) with three or four doses of DNA plasmids encoding four *P. knowlesi* antigens, two pre-erythrocytic stage antigens (*Pk*CSP and *Pk*SSP2) and two erythrocytic stage antigens (*Pk*MSP1_42_ and *Pk*AMA1), followed by heterologous boosting with a cocktail of four recombinant pox viruses expressing the same antigens, confers approximately 20% sterile protection and 60% partial protection against virulent *P. knowlesi* sporozoite challenge (50-100 times the number of sporozoites thought to be required to infect 50% of the monkeys, 50 ID50s)[14]. For example, in **Figure 5.3.1A**, all four controls exhibited the expected rise in parasitemia and required treatment (days 10 to 15); two of 11 vaccinated animals did not become parasitemic; seven exhibited parasitemia that was controlled without treatment; and only two of 11 required treatment for elevated parasitemia (days 12 and 13). We interpret the two parasitemia-negative monkeys as evidence of an effective pre-erythrocytic immune response and the seven monkeys achieving self-cure as evidence of an effective erythrocytic immune response. The reproducible demonstration that approximately 80% of monkeys can be protected against fatal infection by immunization with two pre-erythrocytic and two erythrocytic stage antigens provides proof of principle for our multi-antigen, multi-stage, multi-immune response vaccine concept.

**Figure 5.3.1A -** A multi-antigen, multi-stage vaccine strategy induces protection against parasite challenge in nonhuman primates. Rhesus monkeys were immunized IM 3 times with plasmid DNA encoding two pre-erythrocytic stage antigens (*Pk*CSP and *Pk*SSP2) and two erythrocytic stage antigens (*Pk*AMA1 and *Pk*MSP1_42_), boosted with recombinant poxviruses expressing the same antigens, and challenged with *P. knowlesi* sporozoites. All four controls but only 2/11 vaccinees developed virulent infection (above the 2% parasitemia threshold triggering treatment); 7/11 vaccinees developed mild, self-curing infection (below 2%); and 2/11 vaccinees developed sterile immunity.

### Rationale for Antigen Selection

**CSP**

Several lines of evidence supported the focus on the CSP as a pre-erythrocytic stage vaccine candidate. The CSP is the major coat protein of the sporozoite[15], and monoclonal antibodies against the CSP can neutralize sporozoite infectivity[16, 17]; and provide protection in passive transfer experiments in animal models[18-20]. In murine models, T cell clones specific for CSP can confer protection against challenge with infectious sporozoites[21-24]. In addition, human volunteers protected from malaria by immunization with radiation attenuated *P. falciparum* sporozoites and clinically immune adult residents of malaria endemic areas produce both antibody and T cell responses directed against the CSP[25-30]. However, a large number of clinical trials of CSP-based vaccines using a variety of vaccine delivery systems and powerful adjuvants have shown only limited efficacy even when very high levels of antibody or moderate levels of CSP-specific T cell responses have been induced[31-37]. To date, the most promising CSP vaccine tested has been RTS,S, a recombinant protein based vaccine that has provided a protective efficacy of 47-85% against experimental challenge[38-40] and in exposure in the field[41-43]. These data support the immunogenic and protective potential of recombinant vaccines containing CSP, warranting exploration of further enhancement within an adenoviral vectored vaccine system.

**AMA1**

AMA1 is located in the apical organelles, initially in the necks of the rhoptry organelles, is partially transferred to the merozoite surface at about the time of schizont rupture, and is then taken into the erythrocyte during invasion[44, 45]. Several lines of evidence suggest that AMA1 is critical for parasite survival and hence, is an excellent vaccine target. AMA1 synthesis, stage-specific processing, and localization coincide with the timing of erythrocyte and hepatocyte invasion by merozoites and sporozoites respectively[45-47] and AMA1 has been implicated with a role in merozoite reorientation during the process of erythrocyte invasion[48, 49]. *In vivo*, in nonhuman primate models, immunization of rhesus monkeys with affinity-purified *P. knowlesi* AMA1 protein conferred partial protection against *P. knowlesi* challenge[50], immunization of Saimiri monkeys with baculovirus-expressed *P. fragile* AMA1 conferred partial protection against *P. fragile* and *P. falciparum* challenge[51], and immunization with a yeast-derived *P. falciparum* AMA1 FVO strain protein protected *Aotus* monkeys from homologous *P. falciparum* challenge[52]. In mice, immunization with AMA1 purified from red blood cell extracts of *P. yoelii* infected mice protected against *P. yoelii* challenge[53], and immunization with baculovirus or *E. coli* produced ectodomain of *P. chabaudi* AMA1 protected against *P. chabaudi* challenge[54]. The protective potential of AMA1 has been also demonstrated by passive immunization studies (antibody transfer)[53, 54].*In vitro*, polyclonal sera, monoclonal antibodies, IgG and Fab fragments against AMA1 can inhibit invasion of merozoites and sporozoites in a dose dependent manner[52, 55-60]. Finally, it has been demonstrated that CD4+ T cells acting independently of antibody contribute to protective immunity to *P. chabaudi* infection after immunization with AMA1 protein[61], and that T cells specific for cryptic epitopes on *P. chabaudi* AMA1 afford partial protection against *P. chabaudi* infection in nude mice[62]. Attempts to knock out the *P. falciparum* AMA1 gene have not yielded viable parasites, indicating that the protein plays a vital role in the parasite life cycle[63].

*Pf*AMA1 has been selected by several laboratories for development as a subunit vaccine against malaria. Previous clinical studies in humans have shown that AMA1, when presented as a recombinant protein in two different formulations, was safe and well tolerated. In one study, 5, 20 or 80 ug formulated on 800 ug aluminum hydroxide (Alhydrogel) were used to immunize malaria naïve volunteers[64]. In another study, AMA1 was formulated with the GSK proprietary adjuvant AS02A (Mark Polhemus, personal communication, and Mahamadou Thera, personal communication). Both studies showed the antigen to be safe, and the former study demonstrated functional humoral immune responses based on ELISA, confocal microscopy and GIA.

### Rationale for Vaccine Vector Selection – Recombinant Adenovirus serotype 5

Studies in animal models of malaria[65-70], HIV[71-74], Ebola[75, 76], tuberculosis[77], anthrax[78], human papillomavirus[79, 80], and a variety of other diseases[81, 82], have established that recombinant adenovirus vectors are potent inducers of antigen-specific immune responses and protective immunity against pathogen challenge.

In our laboratories at NMRC, we have evaluated recombinant adenovirus (Ad5 serotype) expressing malaria antigens with regard to their capacity to prime and/or boost antigen-specific immune responses and to induce protective immunity against pathogen challenge. The adenovirus vaccine platform has been extensively evaluated as both a stand-alone vaccine platform (homologous immunization regimen) and in combination with other technologies (heterologous immunization regimens). Data demonstrate that recombinant adenovirus vaccines administered in either homologous or heterologous immunization regimens are very effective at both priming and boosting the host immune response for CD8+ and CD4+ T cell responses as well as antibody responses and can confer sterile protection against parasite challenge (unpublished). These data (described below) confirm the potential of recombinant adenovirus as a vaccine vector and support clinical evaluation of the technology.

### Rationale for Clinical Trial Design

The success of any vaccine will depend upon its ability to effectively stimulate the differentiation of naïve cells and lead to the induction and maintenance of long-lived memory cells that provide a faster and stronger immunological response against subsequent exposure. Recent studies have defined two subsets of memory T cells, *effector memory cells* and *central memory cells*, which may exhibit distinct capacities to provide protective immunity[83-86]. Shortly after antigenic exposure, effector memory cells are the predominant population, and these differentiate over time into central memory cells.

Although the precise roles of the different cellular subsets in providing protective immunity against acute and chronic infections are still being elucidated, a recent study suggests that central memory cells may be better mediators of protective immunity as compared to effector memory cells[86]. Data suggest, therefore, that vaccine efficacy may be significantly impacted by immunization regimens that stimulate central memory cells. Because memory T cells are dynamic populations, which differentiate over time, we have hypothesized that the stage of cellular maturation may influence the ability of memory T cells to respond to recall antigens presented by booster immunizations. Thus increasing the interval between immunizations may allow time for the differentiation of effector memory cells to more effective central memory cells prior to boosting and thereby enhance vaccine efficacy.

- - - 1. Rationale for a long interval between prime and boost

In a series of nonclinical studies in the *P. yoelii* murine model, we have directly evaluated the impact of immunization regimen (both the number of immunizations, and the interval between prime and boost immunizations) on induction and maintenance of antigen-specific immune responses and protective efficacy of different vaccine technologies, including recombinant adenovirus vaccines. Data have established a significant impact of immunization regimen on vaccine-induced immunogenicity and protective efficacy, for recombinant adenovirus vaccines **(Figures 5.3.4A** and **5.3.4B; Table 5.3.4A**), plasmid DNA (data not presented), recombinant poxvirus vaccines (data not presented), and recombinant VEE replicon particle vaccines (data not presented), as well as for heterologous DNA prime/virus boost strategies (data not presented). Specifically, increasing the interval between immunizations resulted in enhancement in the magnitude and frequency of antigen-specific CD8+ T cell IFN-gamma responses and TNF-alpha responses, antigen-specific CD4+ T cell IFN-gamma and IL-2 responses, antigen-specific and parasite-specific antibody responses, and protective efficacy against sporozoite challenge. The impact on protective immunity of increasing the number of immunizations as compared with a single long interval between immunizations has been also evaluated. Data from those studies (not presented) showed that the interval between immunizations is a critical factor, rather than the number of immunizations *per se*. Although it has not been conclusively demonstrated, we believe that the enhanced immunogenicity and protective efficacy resulting from longer intervals may result from boosting the memory cell population following its differentiation to the central memory cell phenotype.

**Figure 5.3.4A**. Effect of immunization interval on induction of antibody responses in mice following homologous immunization with adenovirus vaccine. BALB/c mice were immunized with one or two doses of recombinant adenovirus expressing *Py*CSP according to different regimens. Antibody responses were evaluated by ELISA against recombinant *Py*CSP capture antigen. The regimens for groups 6 and 3, which provided two doses with an eight week interval between prime and boost, induced the highest titers; the regimens for groups 2 and 5, which provided two doses with a four week interval between prime and boost, induced the next highest titers; regimens consisting of two week intervals (groups 1 and 4) or single immunizations (groups 7 and 8) induced comparatively lower titers.

Group 1: 4 wk regimen = boost @ 2 wk post prime / challenge @ 2 wk post boost

Group 2: 6 wk regimen = boost @ 4 wk post prime / challenge @ 2 wk post boost

Group 3: 10 wk regimen = boost @ 8 wk post prime / challenge @ 2 wk post boost

Group 4: 10 wk regimen = boost @ 2 wk post prime / challenge @ 8 wk post boost

Group 5: 12 wk regimen = boost @ 4 wk post prime / challenge @ 8 wk post boost

Group 6: 16 wk regimen = boost @ 8 wk post prime / challenge @ 8 wk post boost

Group 7: 2 wk regimen = one dose / challenge @ 2 wk post immunization

Group 8: 8 wk regimen = one dose / challenge @ 8 wk post immunization

Group 9: naïve control


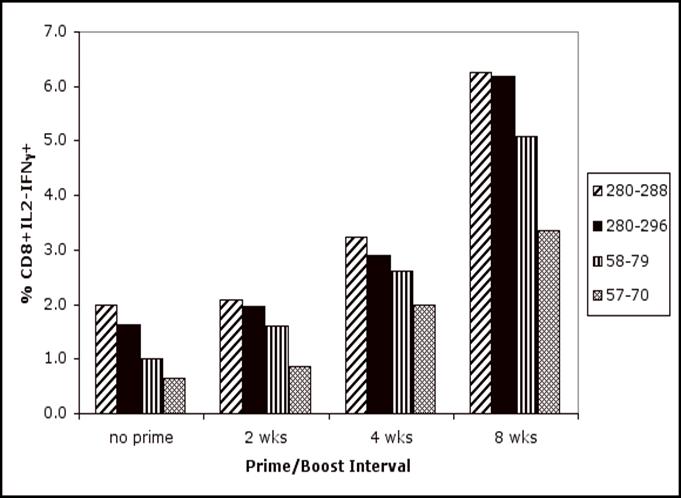


**Figure 5.3.4B.** Effect of immunization interval on induction of T cell responses in mice following homologous immunization with adenovirus vaccine. BALB/c mice were immunized with one or two doses of recombinant adenovirus expressing *Py*CSP according to different regimens as indicated in the legend to Figure 5.3.4A. Antigen-specific CD8+ T cell IFN-gamma responses were evaluated by intracellular cytokine staining (ICS) following *in vitro* restimulation with defined *Py*CSP peptides representing CD8+ or CD4+ T cell epitopes. Histograms represent the means of 6 mice per group, for groups 4 (2 wk interval), 5 (4 wk interval), 6 (8 wk interval) and 8 (no prime) assayed individually. Peptides 280-288, immunodominant CD8+ T cell epitope; 280-296, overlapping CD4+ and immunodominant CD8+ T cell epitopes, 58-79, subdominant CD8+ T cell epitope; 57-50, overlapping CD4+ and subdominant CD8+ T cell epitope As noted for antibody responses, CD8+ T cell IFN-gamma responses were highest in groups with an eight week interval between prime and boost

**Table 5.3.4A.** Effect of immunization interval on protective efficacy against sporozoite challenge in mice following homologous immunization with adenovirus vaccine. BALB/c mice were immunized with one or two doses of recombinant adenovirus expressing *Py*CSP according to different regimens as indicated in the legend to Figure 5.3.4A Protection was measured by presence of parasitemia on blood smear. As with the induction of antibody and T cell responses, protective efficacy was highest in the groups with the longest interval between immunizations. Protective efficacy was also seen in group 7, which received a single dose of vaccine.

We have also demonstrated enhanced immunogenicity by a longer interval between prime and boost immunizations in the *P. knowlesi* rhesus nonhuman primate model. In that model, we compared the effect of 1-month versus 6-month intervals between DNA priming and recombinant poxvirus boosting on T cell and antibody immunogenicity and protective efficacy against sporozoite challenge. As compared to the 1-month interval, the 6-month interval induced more robust IFN-gamma ELIspot responses for both *Pk*CSP and *Pk*AMA1 antigens (although these differences did not reach the level of statistical significance) and a trend to more robust antibody responses for three of the four *Pk*4 antigen components as assayed by ELISA. Importantly, parasitemia levels in the 6-month interval group, but not the 1-month interval group, were significantly different from the control group (*p*<0.05)(Student’s t test)(**Figure 5.3.4C**). These data are consistent with data in the murine model, where a longer interval between prime and boost immunizations resulted in enhanced vaccine-induced efficacy.

**Figure 5.3.4C** Effect of immunization interval on protective immunity in nonhuman primates. Rhesus monkeys were primed with a cocktail of plasmid DNAs encoding two pre-erythrocytic stage antigens (PkCSP, PkSSP2) and two erythrocytic stage antigens (PkAMA1, PkMSP1_42_ ) and boosted at 1 month or 6 months post prime with recombinant poxviruses encoding the same antigens. Immunogenicity and protective efficacy against sporozoite challenge were evaluated. Data points for parasitemia are shown for days when there were 3 or more untreated animals in any group.

Based on these studies of adenovectored malaria vaccines in mice and pox-vectored malaria vaccines in non-human primates, we have chosen to evaluate a long (four month) interval between priming and boosting immunizations in the clinical trial.

- - - 1. Rationale for a short interval between prime and boost

The number of memory T cells has been shown to correlate with the original T cell (clonal) burst size and a variety of factors can influence the outcome of memory T cell formation[87-89]. The presentation of additional antigen early during the induction phase of the immune response has been shown to lead to further expansion of effector cells and to increase the size of memory T cell population[90]. We have hypothesized, therefore, that increasing the initial T cell burst size (clonal burst) could also result in a significant increase in vaccine efficacy. Accordingly, in the *P. yoelii* murine model, we evaluated the effect on vaccine-induced immunity of a booster immunization with DNA or with a recombinant poxvirus given three days after a primary DNA immunization and compared this to a four week interval (**Figure 5.3.4D**)**.** Data suggest that increasing the initial burst size by one or more immunizations spaced at short intervals may positively impact the frequency and magnitude of memory T cells and protective effector function.

We propose to evaluate the potential benefits of a short interval in the clinic by administering two doses of vaccine ten days apart. We have chosen ten days in order to maximally stimulate the initial clonal expansion of the effector cell population, and to accomplish this prior to the development of neutralizing antibodies to the vector particle, which may cause a reduction in the immune response to the antigen encoded within the adenovectored vaccine[91, 92]. A ten day interval is also long enough to assess the safety of the priming dose of vaccine prior to the administration of the boosting dose, thereby enhancing the safety of the volunteers.

Section Addendum: As of 15Nov2007 it has been decided that the short interval concept will not be pursued in this study due difficulties with recruitment so that priority will be directed towards the two dose long interval regimen at this time as this is the most likely regimen to be protective. Reconsideration for the short interval concept could be addressed at a later time.

- - - 1. Rationale for the comparison of one vs two doses of vaccine

We have shown in mice that a single immunization with recombinant adenovirus encoding *Py*CSP can result in robust T cell and antibody responses (data not shown) and is capable of conferring protection against parasite challenge (**Table 5.3.4A**.). Moreover, protection induced by the single vaccine dose can be maintained for at least 6 months post immunization when those same protected mice are then rechallenged (**Table 5.3.4A**); indeed in the one study conducted to date, a one dose regimen was more effective than a two-dose regimen in inducing long-term protective immunity. The efficacy of a single dose immunization with recombinant adenovirus has been also reported by others in the malaria model[66, 67], as well as in other disease models including Ebola[75], tuberculosis[77], anthrax[78], papillomavirus[80], foot and mouth disease[93], and pseudorabies[94]. These data support clinical evaluation and comparison of one and two doses of recombinant adenovirus vaccines against malaria. Should a single dose prove superior in humans, this would provide multiple benefits including ease of administration and reduced costs.

Table 5.3.4A

|  | **2 week challenge** | | **6 month challenge** | |
| --- | --- | --- | --- | --- |
|  | **# protected / # challenged** | **% protection** | **# protected / # challenged** | **% protection** |
| Naïve | 0/10 | 0.0 | 0/14 | 0.0 |
| **Adeno ONE dose** | 9/10 | 90.0 | 4/9 | 44.4 |
| **Adeno TWO doses** | 3/14 | 21.4 | 0/2 | 0.0 |
| **DNA-Adeno** | 7/14 | 50.0 | 2/6 | 33.3 |

Section Addendum: As of 15Nov2007 it has been decided that a one dose regimen will not be pursued in this study due difficulties with recruitment so that priority will be directed towards the two dose regimen at this time as this is the most likely regimen to be protective. Should the two dose regimen prove efficacious, follow on de-escalation studies with one dose can be considered at a later time.

### Previous Human Experience with Adenovirus-vectored Vaccines

There is no previous experience with the NMRC-M3V-Ad-PfCA vaccine in humans. However, NMRC has obtained permission to cite proprietary information regarding the early results of clinical testing of a similar adenovirus-vectored HIV vaccine, VRC-HIVADV014-00-VP, undergoing clinical testing by the NIH Vaccine Research Center (VRC).

VRC-HIVADV014-00-VP is a combination vaccine designed to induce an immune response to HIV *in vivo*. VRC-HIVADV014-00-VP is comprised of four replication deficient recombinant serotype 5 adenoviral vectors that encode proteins from HIV-1 (clade B Gag/Pol, clades A, B, and C Env). These four adenovectors are very similar to the two which comprise the NMRC-M3V-Ad-PfCA vaccine. The identical E1-E3-E4 deleted adenovirus type 5 backbone (GV11D, GenVec, Inc., Gaithersburg, Maryland) is used in the construction of both the VRC-HIVADV014-00-VP vaccine and NMRC-M3V-Ad-PfCA vaccine. A summary of human experience with VRC-HIVADV014-00-VP is included in Appendix A.

### Nonclinical experience with NMRC-M3V-Ad-PfCA

We have conducted nonclinical studies of the Vector Seed Stocks (VSS) for the CSP and AMA1 adenovectors in murine (BALB/c) and swine (Yucatan minipigs, *Sus scrofa*) animal models. In mice, the VSS have been tested as individual vaccines, as components of a five-antigen cocktail (PfCSP, PfSSP2, PfLSA1, PfAMA1, PfMSP1_42_), or as the blended, two-antigen CA vaccine. In swine, only the blended CA vaccine has been tested (data not shown). Results demonstrate that both VSS are immunogenic, as determined by IFN-gamma ELIspot (**Figure 5.3.6 A**) and ICS assays for T cell responses, and ELISA assays for antibody responses (**Figure 5.3.6 B**). Data confirm that both CSP VSS and AMA1 VSS are immunogenic in laboratory animals and that there is no apparent inhibition of immune responses induced by multi-antigen combinations as compared with each antigen alone, for either T cell responses or antibody responses.

**Figure 5.3.6.A & B.** BALB/c mice were immunized with PfCSP VSS or PfAMA1 VSS, either alone or in combination with two or three additional antigens (PfSSP2, PfLSA1, PfMSP1-42), two times at 6 week intervals. T cell and antibody responses were evaluated pre- and at 2 weeks post each immunization by (A) IFNg ELIspot, or ICS, and (B) ELISA. CLAM = *Pf*CSP, *Pf*LSA1, *Pf*AMA1, *Pf*MSP1-42; CSLAM = *Pf*CSP, *Pf*SSP2, *Pf*LSA1, *Pf*AMA1, *Pf*MSP1-42. In Figure A, IFNg ELIspot responses are given for PfCSP, and in Figure B, antibody responses are given for PfAMA1 (primary immunogenicity outcome variables for PfCSP and PfAMA1, respectively, in the proposed clinical study).

Prior to the initiation of use in the proposed clinical trial, the Clinical Seed Stock (CSS) will be used to complete a nonclinical repeat dose safety study and Vaccine Drug Product (VDP) will be used to perform a nonclinical disaster check of the clinical material. Each of these studies is described briefly below.

- - - 1. Nonclinical Safety of the NMRC-M3V-Ad-PfCA Vaccine

A repeat dose safety study in rabbits was completed using CSS to cross-reference in its IND submission relevant safety data obtained from other studies of similar vaccines that share the adenovirus backbone/delivery system or the PfCSP and PfAMA1 transgenes of the NMRC-M3V-Ad-PfCA vaccine. This approach was approved by the FDA in a Pre-IND meeting held 14 April 2005 and reflects the fact that the vaccine belongs to a class of adenoviral vectored products, represented by therapeutic agents (gene transfer) and prophylactic vaccines, which have been previously reviewed by the FDA. Additionally, the malaria antigen inserts included in the NMRC-M3V-Ad-PfCA vaccine are synthetic gene constructs optimized for expression in mammalian cells (codon-optimized) and this type of construct has also been previously reviewed by the FDA in the context of vaccines for malaria and other diseases. The study and results are as follows;

A GLP repeat-dose safety study of the NMRC-M3V-Ad-PfCA vaccine was conducted. NMRC-MV-Ad-PfC and NMRC-MV-Ad-PfA, were cocktailed 50:50 for a final NMRC-M3V-Ad-PfCA dose of either 2 x 10^10^ pu/mL or 1 x 10^11^ pu/mL and administered in 1.0 mL intramuscularly to NZW Rabbits (n = 20/group; 10 male & 10 female) on SD 1, 11 and 32 (one dose in excess of the maximum number of doses to be tested in humans). Separate groups were administered Study Diluent or PBS in a similar fashion to serve as controls.

The vaccine was well tolerated, with no adverse effects on mortality, clinical observations, dermal injection site evaluation, body weights, food consumption, ophthalmologic findings, gross pathology, or histopathology. Likewise, there was no systemic toxicity or myotoxicity associated with vaccine administration. Some transient changes were noted including minimal erythema and edema at the injection site that usually resolved in 2 to 5 days and that did not increase in severity with repeat dose administration. There was also an accompanying histopathological finding of minimal to mild host inflammatory response in the skeletal muscle at the injection site. Transient increases in body temperature were noted within 24 hours of vaccine administration as compared to controls. These temperature elevations were not associated with any adverse clinical observations and always returned to normal prior to subsequent dosing. Likewise, minor, transient changes in clinical pathology parameters such as increased globulin, cholesterol and triglycerides, decreased albumin to globulin ratio, as well as transient shorter prothrombin times (on SD 3 only) were noted. These findings were not associated with any adverse clinical events and not considered to be toxicologically or biologically significant, but rather a part of a host inflammatory/immune response to vaccine administration.

- - - 1. Disaster Check Immunogenicity of the Clinical Lots

To verify that each VDP shows the expected potency and specificity, a non-GLP “disaster check” immunogenicity study was conducted in mice to demonstrate the induction of antigen-specific immune responses. The study involved immunization with each vaccine component individually (NMRC-MV-Ad-PfC and NMRC-MV-Ad-PfA) and with the blended vaccine (NMRC-M3V-Ad-PfCA), utilizing VDPs as the test material.

BALB/c (H-2d) female mice aged 3-5 weeks (n=6/group) were immunized intramuscularly with 1 x 10^8^ pu per dose in 100 µL volume. Five groups of mice received, respectively: (1) control adenovirus lacking the malarial transgene; (2) the NMRC-MV-Ad-PfC component alone, (3) the NMRC-MV-Ad-PfA component alone, (4) the blended NMRC-M3V-Ad-PfCA vaccine, or (5) NMRC-MV-Ad-PfC and NMRC-MV-Ad-PfA administered in separate sites. Two doses were administered on days 0 and 14. Sera were collected pre-immunization and 10 days after each immunization. At 14 days after the second immunization, mice were sacrificed and splenocytes harvested and processed for T cell studies.

Data from this study establish that NMRC-M3V-Ad-PfC and NMRC-M3V-Ad-PfA vaccines are immunogenic in BALB/c mice, as determined by induction of antibodies and T cells specific for PfCSP or PfAMA1 by ELISA and IFN-γ ELIspot, respectively. There was no significant difference in either T cell or antibody responses elicited when NMRC-M3V-Ad-PfC and NMRC-M3V-Ad-PfA were administered in separate sites (Gp 4) or as a blended cocktail in the same site (Gp 5) (p > 0.10). There was no significant difference in antibody responses at any timepoints when either NMRC-M3V-Ad-PfC or NMRC-M3V-Ad-PfA were administered individually (Gps 2 and 3, respectively) or in combination at either the same site (Gp 4) or at separate sites (Gp 5) (p> 0.10). However, there was a statistically significant difference in T cell responses to PfCSP peptide-pulsed targets (peptide 39-47, p = 0.0006; pooled overlapping peptides, p = 0.0002) but not PfCSP transfected targets (p > 0.10) when NMRC-M3V-Ad-PfC was administered individually (Gp 2) or in combination with NMRC-M3V-Ad-PfA at either the same site (Gp 4) or separate sites (Gp 5). There was no significant difference in T cell responses to PfAMA1 (Gp 3 versus Gp 4 or Gp 5).

# Study Objectives

## Part A- Dose-escalation

Primary objective:

- Assess the safety and tolerability of NMRC-M3V-Ad-PfCA, in a dose-escalation design, in healthy, malaria-naïve adults.

Secondary objective:

- Assess the immunogenicity of NMRC-M3V-Ad-PfCA in healthy, malaria-naïve adults.

## Part B- Regimen-comparison

Primary objectives:

- Assess the safety and tolerability of the two components individually (NMRC-MV-Ad-PfC, NMRC-MV-Ad-PfA) and when combined (NMRC-M3V-Ad-PfCA) in a two dose regimen.
- Assess the protective efficacy against sporozoite challenge (*Pf*, 3D7 strain) of the two components individually (NMRC-MV-Ad-PfC, NMRC-MV-Ad-PfA) and when combined (NMRC-M3V-Ad-PfCA) in a two dose regimen.

Secondary objectives:

- Assess immunogenicity of the two components individually (NMRC-MV-Ad-PfC, NMRC-MV-Ad-PfA) and when combined. (NMRC-M3V-Ad-PfCA) in a two dose regimen.

# Study Population

## Subjects

Healthy adult volunteers will be recruited under a protocol approved by the NMRC Institutional Review Board and the Army Surgeon General’s Human Subjects Research Review Board (HSRRB). Volunteers will be enrolled if they meet eligibility criteria described in Section 7.3 below. Enrollment will be restricted to adenovirus 5 seronegative or low-titer individuals (< 1:500, by a luciferase-based neutralizing antibodyassay; VRC, Bethesda[95]) for Part A of this trial while Part B will be a mix of Ad5 serpositive and seronegative with a minimum of (n/2)-2 low-titer volunteers per study group. This will allow continued assessment of the vaccine in the volunteers for whom the vaccine is more likely to be reactogenic (low-titer) while permitting evaluation of safety, tolerability, immunogenicity and efficacy in higher titer individuals as well. The incidence of adenovirus 5 seronegativity in the U.S. population is estimated to be between 40 and 67% with an incidence of 63% from a study using volunteers from the mid-Atlantic region[96]. Therefore the volunteer population of Part B will also be more representative of the target population as a whole. Prior to enrollment, volunteers must demonstrate adequate comprehension of the informed consent process by passing a written assessment of knowledge about the conduct, risks and benefits of participating in the trial with a score of at least 80% (Appendices J & K) with the requirement of satisfactorily verbalizing an understanding of any questions answered incorrectly.

For Part A- It is anticipated that approximately 40 volunteers will be screened to recruit fourteen eligible volunteers for participation. Twelve will be scheduled to receive one immunization (six per dose group) and two will be designated as alternates. The alternates will only receive immunization if one or two of the twelve volunteers scheduled for immunization drop out prior to receiving the immunization. In the case that one of the twelve volunteers drops out, one alternate will serve as a replacement and be immunized; if two of the twelve volunteers drop out, both alternates would serve as replacements and be immunized.

For Part B- It is anticipated that approximately one hundred and seventy-five volunteers will screened to recruit up to sixty eligible volunteers for participation in one of the three groups/regimens with a minimum of 10 to 20 volunteers for each regimen (plus a total of twelve infectivity controls participating for the purpose of sporozoite challenge only). Since the number of volunteers is large and the immunization and challenge schedule is complex, each dose regimen group will be split in half with two cohorts for each group. One set of cohorts (i.e. approximately 5-10 from each group: 3 thru 5) will be immunized and challenged at up to a 3 week stagger from the other set of cohorts. The Infectivity Control group will be split similarly (eg. 6/6) to accompany each of the cohort sets for challenge and immunologic follow-up**.**

Fourteen infectivity control volunteers will be recruited and screened for experimental challenge. Twelve will be challenged and two will be alternates and only participate in challenge if one or two of the scheduled control volunteers withdraw prior to challenge. The challenges will also be staggered by up to three weeks apart depending on mosquito availability, as mentioned above so that there will be eight infectivity controls asked to present for each of the two cohort challenges, but only 6 will be challenged with each cohort (the seventh and eighth being an alternate and only challenged if a scheduled control fails to show up, or cannot be challenged for some reason on that day). If less than 6 show up for a challenge all together a ll of those volunteers will be challenged down to a minimum of 4 volunteers. If less than 4 volunteers show up for challenge the challenge will need to be rescheduled.

## Sample Size and Power Consideration

The primary outcome variable for this initial Phase 1 study is safety, since the primary objective of this phase is to provide assurance that the frequency of severe vaccine related adverse events is below a certain threshold, indicating that it is acceptable to continue testing the vaccine in larger numbers of volunteers.

For example, in the dose escalation phase, 12 volunteers will receive injections with the vaccine. If none of these volunteers experience severe or serious vaccine related adverse events, then we have a 45% level of confidence that the true rate of these events in the general population would be less than 5% (assuming everyone in the general population received the vaccine). Similarly, no events in 12 volunteers provides a 70% level of confidence that the true rate of severe or serious vaccine related adverse events in the general population would be less than 10%, and we would have a 91% level of confidence that the true rate of such events in the general population is less than 20%. These figures are determined by using an Upper Bound Calculation for No Events based on The Rule of Three which is: Given no events in n trials, the 95% upper bound on the rate of occurrence is 3/n. [97].

With the inclusion of additional volunteers during the regimen-comparison phase of the trial, the level of confidence increases (as long as no severe or serious adverse events related to vaccine administration are observed). For example, with an anticipated n of between 24 to 72 (total number of anticipated vaccinees in the trial), the confidence levels that the true rate of severe or serious vaccine related adverse events in the general population is less than 5%, 10% or 20% are 69.9-97.3%, 90.9-99.9% and 99.18-99.99%, respectively. This would represent a substantial increase in confidence, providing sufficient assurance of safety to justify the testing of larger numbers of volunteers in subsequent trials.

The sample size (10 to 20 per group immunized, in the regimen-comparison phase) is also powered sufficiently to show statistically significant differences between regimens in immunogenicity and in protective efficacy. In anticipation of some attrition, twenty volunteers per group will attempted be recruited and a minimum of 10 to 20 volunteers per group (12 if just one group is immunized) will be immunized depending upon the number that present on day of immunization(s) and day of challenge. In two previous vaccine studies conducted by NMRC, sample sizes of five volunteers per group resulted in statistically significant differences in immunogenicity when groups were compared[98, 99].

## Subject Selection Criteria

### Inclusion Criteria

- Between the ages of 18-50 (inclusive)
- Negative results of HIV ELISA, HBsAg, anti-HCV antibody, and no other clinically significant abnormal laboratory results from screening
- Adenovirus 5 seronegative or low titer (reciprocal titer < 1: 500; Part A only)
- Able to provide written informed consent
- Complete an Assessment of Understanding and verbalize an understanding of any questions answered incorrectly.
- In good general health without clinically significant medical history or physical exam abnormalities at screening
- Willing to continue immunogenicity and clinical follow-ups for one year and telephone or mail (electronic/U.S. Postal) contact as a long term safety monitoring provision for an additional four years (totaling five years of participation; immunized volunteers only)
- Participants agree to use effective means of birth control (an FDA approved contraceptive, abstinence) between screening and 60 days following last clinical study visit or able to provide evidence of no reproductive capability.

### Exclusion Criteria

- Plan to participate (or have participated in the last 30 days) in any other research study including an investigational drug or device.
- History of malaria infection, exposure to malaria infection (i.e. travel to a malaria endemic region within 2 years prior to first immunization, history of residence of > 5 years in area known to have significant transmission of *P. falciparum)*, or receipt of a candidate malaria vaccine containing either the CSP or AMA1 antigens.
- Significant cardiovascular, hepatic, renal, hematologic, or immunologic abnormality either by history or laboratory examination. Includes bleeding or seizure disorders.
- Determined, using a non-invasive cardiac risk assessment tool, to have a cardiovascular risk profile that might place them at increased risk if they should develop malaria as a result of the study. (Part B only)
- A positive result on HIV testing at screening.
- A positive result on Hepatitis B or C testing at screening.
- An Adenovirus serotype 5 titer > 1:500 (Part A only).
- Splenectomy
- Use of immunosuppressive drugs (excluding nasal steroids or topical steroids) within thirty days of first scheduled immunization (Trial Day 0)/challenge
- Volunteers who have received immunizations (live or killed) within thirty days of either the first or second scheduled immunization.
- Volunteers who require immunizations for travel or other purposes during the immunization phase of the trial
- Volunteers receiving blood products within 120 days of immunization/challenge
- Serious Adverse Reaction to other vaccines (such as hives, anaphylaxis, respiratory difficulty, angioedema or abdominal pain [excluding abdominal pain caused by oral vaccines such as oral typhoid vaccine])
- Any other finding which would increase the risk of having an adverse outcome during treatment should the volunteer develop malaria as a result of the study

Additional Criteria for Females:

- Pregnant or breast-feeding females or those planning to become pregnant within the next year

# Study Design

## Summary

This open label trial will begin with a dose-escalation phase (Part A) (Groups 1 and 2) and then enter a regimen-comparison phase (Part B) (Groups 3, 4 and 5). The timeline below provides a summary of the study design and time course.

Volunteers will be sequentially assigned to the study groups with the restriction that common HLA types (defined members of HLA supertype families) will be distributed as evenly as possible among groups. In part A the goal is six volunteers per group, therefore eight volunteers will be enrolled (allowing two alternates). In part B the goal is to have at least a total of ten volunteers per dose regimen group (for groups 3, 4, 5) who can be evaluated for vaccine-induced immune responses and protective efficacy. Therefore, in part B, using the up to 3 week cohort stagger described previously, a total of ten to twenty volunteers will be assigned to each dose regimen group and immunized to allow for drop-outs prior to sporozoite challenge. A total of twelve volunteers will serve as infectivity controls for the sporozoite challenge as well. The anticipated drop-out rate (two volunteers per group) is based on previous experience.

As stated previously the groups would be prioritized based on recruitment/enrollment thresholds over the 90 day recruitment period. The basic priority for the regimens/groups is: 1) NMRC-MV-Ad-PfC, 2) NMRC-M3V-Ad-PfCA 3) NMRC-MV-Ad-PfA. This prioritization takes into account noted recruitment issues and considers the fact that the CSP antigen contained in the vaccine alone has been shown to protect in other clinical vaccine studies while AMA1 and the combination of the two antigens still await proof of efficacy in clinical trials (discussed in Section 5.3.2). In addition, in NHP models it is the combination of antigens that has proved protective. The table below outlines the group assignments based on the number of people recruited and fully enrolled after successful screening. Note that with all three groups being filled a goal of 16-20 per group is desired. However as the number of groups to be designated for filling drop with declining enrollment numbers the minimum number of volunteers for a group drops to 10 while the maximum remains at 20. In addition, if the total number of immunized volunteers goes below 30 it may be possible to challenge all at one time with only 6 infectivity controls as opposed to cohorting on two separate days for challenge with 6 infectivity controls needed for each day.

| # Recruits for Part B | Part B Study Composition | |
| --- | --- | --- |
|  | Group | n |
| n = 60 | Group 3  Group 4  Group 5 | 20  20  20 |
| n=40-59 | Group 3  Group 4  Group 5 | n/3  n/3  n/3 |
| n = 39 | Group 3  Group 4  Group 5 | 13  13  13 |
| n=21-38 | Group 3  Group 4  Group 5 | n/2  n/2  0 |
| n = 20 | Group 3  Group 4  Group 5 | 10  10  0 |
| n = 12-19 | Group 3  Group 4  Group 5 | 0  12-19  0 |

The dose-escalation phase is formally separated from the regimen-comparison phase by three months to allow adequate assessment of safety in the first 12 volunteers receiving the vaccine (Group 1, n=6; Group 2, n=6). Safety reviews will be conducted utilizing data that extends two weeks past the Group 2 immunization. The SMC will review the data and a report compiling safety review data will be prepared and submitted to the Sponsor, USAMRMC ORP, relevant IRBs and FDA. Immunogenicity assessment from Part A is not a prerequisite for continuation to Part B as there is no immunologic correlate of protection, but will be submitted for review prior to challenge of Group B per FDA recommendations in the pre- IND meeting of April 14, 2005. In addition, any comments and correspondence by the FDA will circulated to the Sponsor, USAMRMC ORP and relevant IRBs.

The regimen-comparison phase is based on nonclinical data obtained by NMRC and by others demonstrating that the immunization regimen can significantly impact vaccine-induced immune responses and protective efficacy (Section 5.3.4 provides the rationale for the clinical trial design).

In Groups 4 and 5, vaccines based on CSP and AMA1 will be assessed separately. These results when compared to the results from volunteers who received the two antigen vaccine combination will allow for an evaluation of interference or synergy. Both interference[100, 101] and synergy[102] have been seen in murine, swine, and nonhuman primate models of malaria utilizing genetic vaccines.

## Go-No Go criteria for initiating the clinical trial

The following criteria must be met in order to proceed with the clinical trial: (1) nonclinical safety studies: no serious test article-related toxicities in rabbits; (2) disaster-check immunogenicity study in mice: significant immunogenicity relative to controls for both *Pf*CSP and PfAMA1 (p<0.05 in 2-tailed t test)(see Section 5.3.6.2); (3) allowance of the IND by the FDA and (4) IRB approval of the human use protocol.

## Go-No Go criteria for transition from Part A to Part B

Progression to immunization in the regimen-comparison phase (Part B) will require: (1) approval by the Safety Monitoring Committee of Part A safety and tolerability data: (2) submission of a written report summarizing Part A safety findings to the Sponsor, USAMRMC ORP, relevant IRBs and FDA and; (3) submission of any comments and correspondence by the FDA regarding the transition from Part A to Part B to the Sponsor, USAMRMC ORP and relevant IRBs; (4) Minimum of 12 volunteers available for immunization as outlined above in Section 8.1.

Further immunogenicity data (other than proof that each construct induces antigen-specific immune responses in the “disaster check” immunogenicity study – see section 5.3.6.2) will not be required for progressing to the regimen-comparison phase because there is no established immunological correlate of protection in malaria. Similarly, there will be no immunological criteria established for conducting the challenge since immunological data cannot predict the likelihood of protection. While non-human primate (NHP) studies can provide proof-of-principle for a general approach to vaccine development, there are no data to correlate immune responses or protective efficacy in NHP with human results, and there is even some evidence that in certain circumstances such NHP studies may give incongruous results[103]. The lack of immunologic correlates, human or NHP, is so apparent among malaria vaccinologists that the Malaria Vaccine Initiative has included development of such correlates as one of its top priority initiatives as well as declaring that until such correlates can be identified, “. . . human volunteers are the only means to assess [vaccine] candidate efficacy and duration of protection.”[104] Therefore, volunteers in good health in this study will still be challenged without regard to the immunogenicity data obtained following vaccination because there is no way to predict which volunteers will be protected based on immunological data alone, whether it is from humans or NHP. However, though an immunogenicity assessment from Part A is not a prerequisite for continuation to Part B, an assessment will be submitted for review prior to challenge of Group B per FDA recommendations in the Pre-IND Meeting of April 14, 2005. All comments and correspondence with regard to this will be circulated to the Sponsor, USAMRMC ORP and relevant IRBs.

## Go-No Go criteria for transition to future clinical trials

Protection data are preferable in order to select the best regimen for testing in future clinical trials, again because there is no clear immunological correlate of protective immunity. However, evidence of protection such as delayed or absent parasitemia in immunized volunteers is not required for transition to follow-on trials, which may involve prime-boost approaches that could substantially increase the protective efficacy of the NMRC-M3V-Ad- PfCA vaccine. However, we will require evidence of significant immunogenicity in order to proceed with future clinical trials. We will require positive immune responses relative to control (naïve volunteers) for T cell responses against PfCSP and antibody responses against PfAMA1, in the absence of significant inhibition in the two antigen combination relative to each antigen alone, as defined by:

- IFN-γ ELIspot (PfCSP): > 50 spot forming cells per million (for summed responses against all peptides)

- Antibody ELISA (PfAMA1): OD405 @ 1/100 serum dilution > mean + 2 std dev control

# Study Duration

Each immunized volunteer will participate actively (presenting regularly for clinical visits) for approximately one year from first immunization through completion of clinical visits and data collection. For an additional approximately four years, contact (by phone or mail, electronic/U.S. Postal, unless a face-to-face meeting is clinically indicated) will occur annually (every 12 months +/- one month) to collect long-term safety outcome data (Appendix R).

The anticipated duration of screening and active immunized volunteer participation for parts A and B of this study from the time of first screen to last clinical visit is estimated to be about one year. Collection of long term safety data will continue an additional four years.

Infectivity control volunteers will participate from enrollment for up to 1 year out from challenge. This is to permit further evaluation of the long term immune response to the challenge as compared to those who were immunized.

# Study Procedures

## Recruitment

Volunteers will be recruited from the DoD beneficiary population (active duty or retired military and dependents) as well as from the community. For the volunteers from the community, per SECNAVINST 3900.39D, mechanisms for unanticipated hospitalization due to research related injury will be handled by either SECNAV Designee Request for the study (if granted), and/or care provided for at an Army hospital or clinic per AR 70-25 as outlined in Section 26 below. Recruitment will be in a non-coercive manner and will be performed by civilians when done within the military to avoid any perception of coercion by rank. Advertisements will be in various forms of printed flyers and publications and other media forms such as radio or internet may be explored or considered. In addition, interested active duty military will be given an approval form required to be signed by their supervisor/superior and presented at screening before they will be allowed to continue beyond screening (Appendices H, I & FF). Materials used for recruitment will be prepared and submitted for review and approval by the relevant IRBs and the USAMRMC HSRRB prior to use as they are developed including briefing material.

## Screening Visit

After the initial recruitment meeting volunteers will first meet with a member of the study team and review the details of the protocol, ask any questions, complete and review an Assessment of Understanding for the pertinent part of the study and sign the Informed Consent document (Appendices E, F & BB) and HIPAA Authorization (Appendices II, JJ & KK), and all associated consents, forms and authorizations including HIV testing (Appendices AA & GG). Volunteers must demonstrate adequate comprehension of the informed consent process by passing a written assessment of knowledge about the conduct, risks and benefits of participating in the trial with a score of at least 80% (Appendices CC, J & K) with the requirement of satisfactorily verbalizing an understanding of any questions answered incorrectly. At this initial study visit, information will be collected as part of the registration process for identifying, contacting and compensating the volunteer (thus the SSN) for their participation. It likewise provides emergency contact data as well as military/work data in case commands need to be notified of an emergency or a reportable disease identified on screening. The remaining demographic data such as ethnic background assists with research as some ethnicities have a higher preponderance of certain HLA types then others. Some of this data will be put in a database indicated in the Informed Consent Document section entitled “NMRC Clinical Trials Program Database”. This section and the *“Screening”* on the ICD explain in detail to the volunteer the intended purpose. In addition, information will be collected to confirm that health and medical history eligibility requirements are met (including review of any known allergy to chloroquine in the event treatment of malaria is required; if the volunteer has intolerance to chloroquine, an alternative medication, atovaquone/proguanil (Malarone), will be used.). This process will include:

1. A review of the volunteer’s medical history per volunteer’s report.
2. A physical examination including collection of vital signs.
3. Clinical Laboratory evaluation (at NNMC Clinical laboratory) including:
   - Complete blood count (CBC)
   - Serum chemistries (Creatinine, Glucose, AST, ALT,)
   - Urinalysis
   - Hepatitis B surface antigen (HBsAg) and Hep C antibody,
   - HIV ELISA (Western Blot if indicated). (Pre-test HIV counseling will be provided and consent obtained prior to evaluation of HIV serology.)
   - Urine β-hCG (for all females of reproductive potential)
   - G6PD testing for Part B volunteers, if no proof of prior testing available. (If G6PD deficient then atovaquone/proguanil will be used for treatment should those volunteers become parasitemic.)
4. Adenoviral serology (Contract lab: NVITAL at the NIH Vaccine Research Center).
5. Intermediate level Class I and II HLA typing (Contract lab: DOD Bone Marrow Center).

A baseline 12 lead ECG will be obtained on all volunteers taken to malaria challenge. This will be either an existing ECG obtained within one year of the date of challenge, or a new ECG that will be obtained by the study team prior to challenge. The purpose of the ECG will be to have a baseline ECG to serve as a comparison in case an ECG is clinically indicated during the challenge follow-up.

Once the requisite number of volunteers have been successfully screened and meet all criteria, subjects will be sequentially assigned to immunization groups for Part A and similarly for Part B after that recruitment and screening process. Infectivity controls for Part B will be recruited and screened in separate process. Based on seropositivity rates for adenovirus serotype 5 it is expected that approximately 18-20 volunteers will need to be enrolled and screened for Part A with only the first 12 who meet all criteria being vaccinated. All others who do not meet criteria will be disenrolled. Similarly for Part B (those immunized and infectivity controls) it is expected that 90-95 volunteers will needed to be screened to obtain the 58 for this part that meet all criteria. Those not meeting criteria will be disenrolled. Thus for the entire study it is anticipated that a minimum of 115 volunteers will need to be enrolled and screened to meet the 84 total that is needed with an expectation of a max of 175 volunteers to be screened as previously mentioned in Section 7.1.

As mentioned previously in Section 8.1. The recruitment/screening period for Part B will take place over a 90 day period. Near the end of that 90 day period the group assignment determination will be made as outlined in Section 8.1. If the maximum number of volunteers to be immunized (n=60) is fully enrolled before the end of that period then it is not necessary to wait the full 90 days, assuming all other criteria are met.

## Immunization Visits

The vaccine components will be kept in a monitored, alarmed, -70^O^C freezer used exclusively for vaccine storage, and thawed to room temperature within four hours of administration. The two antigen components will be in separate, single-use, screw-capped sterile cryovials. A pharmacist or appropriately trained personnel will prepare each syringe, according to the established SOP (included as Appendix B). Vaccine will be administered intramuscularly.

### Prior to Immunization

#### *7 days Prior to Immunization*

- Abbreviated physical examination with vital signs collected and recorded.
- Medical history review - volunteers will be asked about any symptoms of any type they may be experiencing or any adverse experiences that have occurred since the previous study visit.
- Laboratories –
  - Urine B-hCG in females
  - Research serum/blood collected/stored for immunology (see section 10.7.2)

In addition, volunteers will be given temperature logs (Appendix T) and digital thermometers at this time. They will be instructed on their use and will keep a temperature log starting 7 days prior to immunization, recording oral temperature twice a day. The temperature log will be handed in and reviewed by study team on the Day 7 visit following each immunization received.

#### *Day of Immunization prior to injection*

- Abbreviated physical examination with vital signs collected and recorded.
- Medical history review - volunteers will be asked about any symptoms of any type they may be experiencing or any adverse experiences that have occurred since the previous study visit.
- Laboratories –
  - CBC
  - Serum Chemistries (Creatinine, AST, ALT,)
  - Urinalysis
  - Urine B-hCG in females

The following adverse events associated with vaccine immunization constitute absolute contraindications to further administration of vaccine. If any of the following adverse events occur during the study, the subject must be withdrawn and must be followed until resolution of the event:

- - - Anaphylactic or any other immediate-type, severe systemic reaction following the administration of vaccine.

In addition, pregnancy will constitute an absolute contraindication to further administration of vaccine or challenge. If a subject were to become pregnant after immunization the subject would be referred to an obstetric care provider for follow-up. The subject would still be asked to continue follow-up visits and, at the direction of the obstetric care provider, have follow-up routine safety labs (complete blood count, serum chemistries as defined above, but no immunologic follow-up labs unless directed). The subject would also be asked to report on the outcome of the pregnancy.

The following adverse events constitute relative contraindications to administration of vaccine at that point in time. If any one of these adverse events occurs at the time scheduled for immunization, the subject may be vaccinated at a later date, within the time window specified in the protocol, or withdrawn at the discretion of the PI. The subject must be followed until resolution of the event, as with any adverse event.

- Acute illness at the time of enrollment including but not exclusive to ocular, respiratory, gastrointestinal, genitourinary, dermatologic or musculoskeletal complaints or other concerning symptoms as determined by the clinical investigator. Should any questions arise the Medical Monitor would be consulted.
- Oral temperature of ≥ 38°C (100.4°F) at the time of immunization.

### Immunization

Immunization will be via standard IM injection in the deltoid muscle. The left arm or non-dominant arm will be used unless there is a compelling reason to use other arm, at which point clinical judgment will be deferred to and clear documentation will be provided. If assigned to a group in which two immunizations will be received, the same arm will be used for each immunization.

### Post-Immunization

Volunteers will remain for an observation period of approximately 30 minutes post-immunization to allow the study team to confirm that there are no immediate reactions that occur in response to vaccination and to provide immediate care for any reactions that do occur.

Observations of the injection site and any spontaneously reported events will be recorded on the visit worksheet. Vital signs (oral temperature, heart rate, blood pressure (sitting), respiratory rate) will be recorded at the end of the observation period.

## Post Immunization Follow-up Visits

Volunteers will receive either one or two immunizations depending on group assignment. Following each immunization the same schedule detailed below will apply:

- Days -7 through 7 (i.e. beginning seven days before FIRST immunization and continuing for seven days following each immunization), volunteers will keep a temperature log (Appendix T), recording oral temperature twice a day with a digital thermometer that will be provided. The temperature log will be handed in and reviewed by study team on the Day 7 visit following each immunization received.
- Day 1 - Volunteers will be contacted by telephone for a brief safety check and to review diary card information and procedures. (Part A Volunteers only)
- Days 2, 7, 10, 14, and 28 (Part A) and days 1, 2, 3, 5, 7, 14 and 28 (Part B) following each immunization.
  - Abbreviated Physical Examination (including collection of vital signs)
  - Collection of solicited and unsolicited adverse events including:
    - Symptoms:
      - local reactogenicity - pain, redness, swelling, limitation of motion, regional lymphadenopathy
      - systemic adverse events - fever, chills, diarrhea, malaise, myalgia, arthralgia, stomach upset or vomiting.
      - potentially related to upper respiratory infection or conjunctivitis – including cough, coryza, congestion, pharyngitis, eye pain or irritation.
      - Potentially related to a urinary tract inflammation/infection – including frequency, dysuria or hematuria.
    - Signs:
      - tenderness, induration or erythema at the injection site
      - regional lymphadenopathy
      - signs suggestive of upper respiratory infection, conjunctivitis, gastroenteritis or urinary tract inflammation/infection.
  - Safety Laboratories (Clinically significant abnormal values will be tabulated and a trend analysis performed as indicated at any point during the study)
    - - CBC
      - Serum Chemistries (as defined above) Part B volunteers on days 2,7,14,28 only
      - Urinalysis (Day 0 and 14 only)
- Research serum/blood collected/stored for immunology (see section 10.7.2) on days 10 and 28.for Part A and on day 28 following first immunization, prior to second immunization, day of challenge, and 28 days post challenge for Part B.
- Days 0 through 28 - In the case that a volunteer is found to have an upper respiratory infection or conjunctivitis, gastrointestinal illness or urinary tract infection/inflammation at any time during this period, the volunteer will have specimens collected for adenoviral culture from the appropriate site to include swabs of nasopharynx or conjunctivae, or samples of urine or stool. If any samples are positive for adenovirus, cultures will be sent to a research laboratory for further analysis to assess for the presence of specific sequences from the vaccine vector using procedures based on previously published methodology. (Wang et al. 2003). Specifically, PCR analysis will be performed with DNA from the adenovirus isolate and primers within the *P. falciparum* gene and flanking the region where the *P. falciparum* gene is inserted into the adenovirus genome.

## Compliance Ranges and Holding Rules

**Part A:** Systemic adverse events: The initial immunization of Group 2 (dose escalation) will be put on hold, subject to discussion by the investigators, medical monitor and Sponsor as required, if during a four-day period after immunization (day of immunization and 3 subsequent days), 2/6 subjects immunized in Group 1 (low dose) report grade 3 (severe, preventing daily activity) systemic/general solicited or unsolicited adverse events considered related to immunization, that last at least 48 hours without decreasing in intensity OR for any SAE related to immunization.

Laboratory adverse events: Immunizations will be put on hold if 2/6 subjects develop a Grade 3 laboratory adverse event considered to be associated with immunization.

If immunizations are put on hold, the Medical Monitor, the SMC, Sponsor, USAMRMC ORP relevant IRBs, and FDA will be notified. Lifting the hold will follow approval for continuation from the Medical Monitor and the SMC as well as authorization from the IRBs.

A report summarizing all safety findings from Part A will be submitted to the SMC, Sponsor, USAMRMC ORP, relevant IRBs and FDA.

**Part B:** Following the first immunizations in Groups 3 thru 5, the study will be put on hold if during a four-day period after immunization (day of immunization and 3 subsequent days), 6/20 (or > 30% if less than 20 per group) of subjects (e.g. 3/10 per cohort) report grade 3 (classified as severe and also preventing daily activity despite treatment) solicited or unsolicited adverse events found to be clinically significant and considered related to immunization, that last at least 48 hours without decreasing in intensity OR for any SAE probably or definitely related to immunization, (regardless of time elapsed since immunization) as confirmed by the investigator and by the medical monitor.

Laboratory adverse events: Immunizations will be put on hold if 6/20 (or > 30% if less than 20 per group) of subjects (e.g. 3/10 per cohort) develop a Grade 3 laboratory adverse event of the same laboratory parameter and is considered to be associated with immunization and found to be clinically significant, as confirmed by the investigator or by the medical monitor.

Following the 16 week dosing interval all immunizations will be completed so there will not be further immunizations to hold. Only sporozoite challenge will remain; this can be delayed or cancelled for any volunteer whose health status is such that challenge could pose an inappropriate risk. Any volunteers that are not healthy at the time of challenge need clearance by the investigator with the medical monitor to be additionally be consulted as appropriate.

## Experimental Challenge with Malaria Infected Sporozoites

In Part B, a challenge with viable, infectious sporozoites two to four weeks after the last immunization will be conducted using five infectious mosquito bites in order to assess protective immunity and allow for evaluation of surrogate markers of protection. As a fair amount of time will have elapsed for most volunteers from the initial consent process and the imperative for the volunteers to understand the challenge process and follow-up procedures to ensure their safety, the process and follow-up will be reviewed and an Assessment of Understanding (Appendix Y) will be completed pertaining to the challenge with a review of any incorrectly answered questions prior to challenge. This assessment tool will also be used for the Infectivity Control group as part of their informed consent process.

To prepare infected mosquitoes, *P. falciparum* asexual and sexual erythrocytic stage parasites are grown in normal human erythrocytes using standard culture medium containing 10% heat inactivated (56^O^C, 30 min) normal human serum. All erythrocytes and serum are obtained from donors at low risk for both hepatitis and HIV infection and whose serum does not contain hepatitis B surface antigen or antibodies to hepatitis C, *Treponema pallidum* or HIV. This blood and serum for culture are purchased from a commercial source and each shipment carries a certificate of analysis certifying that the blood products were negative or non-reactive for the above pathogens.

The mosquito species is *Anopheles stephensi*. Colonies of *A. stephensi* have been maintained at NMRC and the Walter Reed Army Institute for Research (WRAIR) for several decades. To date, no adventitious agent of disease has been identified as transmitted by these colonies of mosquitoes. Based on theoretical concerns raised by the FDA, we have switched the mosquito larvae to a fish-food diet that does not contain any bovine products.

Approximately 14 to 21 days before each challenge, female mosquitoes from our secure insectaries will be infected with gametocytes of the *Pf* NF54 strain, or the 3D7 clone of NF54, through membrane feedings on the *P. falciparum* cultures.

### Challenge procedure

In order to challenge volunteers, mosquitoes infected via membrane feeds 14 to 21 days prior to challenge and containing sporozoites in their salivary glands will be allowed to feed on the volunteers. This will be done in the absence of chloroquine or other antimalarial drugs. For each volunteer, five mosquitoes will be allowed to feed. The mosquitoes will then be dissected to confirm the presence of a blood meal, and determine the infectivity rate and the salivary gland score. Additional mosquitoes will be allowed to feed until a total of five infected mosquito bites, with a minimum 2+ salivary gland score (Appendix C), have been achieved.

### Parasitemia

Microscopy will be performed on thick smears using a standard, validated SOP (Appendix D) by an experienced microscopist examining 10 ul of blood under high power, with positive findings confirmed by a second reader. Parasitemia will be defined as at least two malarial parasites noted on the thick smear and quantified as the number of parasites per ul. Quantification is achieved by placing 10 ul of blood evenly onto a 1 by 2 cm scored area of the slide and then, following Giemsa staining, reading one tenth of this area (five passes along the 1 cm direction with a 100x objective = approximately 360 hpf) and counting the number of parasites. At a later time point, the results of blood smears may be correlated with quantitative real time polymerase chain reaction (QRT-PCR) from the same sample to evaluate the potential of QRT-PCR to detect parasitemia earlier than microscopy.

### Management of Volunteers POST CHALLENGE (PC)

Volunteers will be instructed with regard to the signs and symptoms of malaria including: fever (oral temperature of > 38ºC [100.4°F]), chills, rigors (shaking chills), sweats, moderate to severe headache, dizziness, malaise, fatigue, insomnia, joint pain, muscle pain, neck ache, nausea, vomiting, stomach/abdominal cramps, or diarrhea. Because there is good reason to assume that the challenge controls exposed to malaria-infected mosquitoes will develop blood stage malaria, and that the immunized volunteers may also develop blood stage malaria, it is critical that monitoring be performed.

Following challenge, volunteers will be observed for 30 minutes for an immediate reaction to the mosquito bites. The local area surrounding the location of the mosquito bites will be inspected and vital signs will be collected. Any AEs, solicited or unsolicited, will be recorded.

The time period from the point of exposure (challenge day) to the time of detection of malaria symptoms is generally 7-12 days. Therefore for the first six days volunteers will be allowed to return to their usual routine. Volunteers will be reminded of 24 hour contact information for the study team and will be reminded to contact the study team immediately in the event of any signs or symptoms of illness.

Follow-up from Day 7 to Day 17 post challenge will require overnight stays at a monitored site, such as a hotel, unless the PI determines that it is safe for a particular volunteer to spend one or more nights during the monitoring period at his/her home. Regardless, twice daily checks with the study team will be performed to provide close monitoring of blood smears, and clinical assessments. This will be achieved as follows: during the day, volunteers are free to pursue normal activities, but each evening, they are required to report to the venue for overnight stay, check in with the study team, remain at the hotel during the night, and report to the study staff again in the morning prior to leaving. Blood smears will be collected daily if volunteers are without symptoms, and at least twice daily if symptomatic. A nurse will be available at the hotel 24 hours a day, and each night a study physician and microscopist will remain at the hotel to care for volunteers. If blood smears are negative, daily examinations and blood smears will continue until Day 17. Following Day 17 and for an additional 4 days through day 21 volunteers will be followed in the clinic with daily smears and clinical assessments. If at any time post challenge, fever or other signs and symptoms of malaria develop, blood films will be obtained..

Volunteers with malaria, defined as finding malaria parasites on thick smear examination, will be treated under direct observation. In the rare event that a volunteer needs to be hospitalized, this will be done at NNMC, Bethesda. Volunteers will be instructed not to travel outside of the Washington, D.C. metro area from the day of challenge to eight weeks after challenge. If a volunteer must travel, proper arrangements will be made to ensure maximum contact (e.g. numbers of mobile phone, pager, home phone) between the volunteer and the study team and if this is not possible, volunteers will be treated with oral medication presumptively for malaria (in the same fashion as described below in the event of a positive blood smear).

An immunized individual who does not have complete protection may have a prolongation of the pre-patent period post challenge. The pre-patent period for *P. falciparum* in humans normally ranges from 9-12 days. In our previous studies the pre-patent period varied from 7-21 days[105, 106]. It is possible that due to varying methods of immunization, it could be longer than 21 days. For this reason, immunized volunteers who are still negative 21 days post challenge will be followed for an additional 7 days with every other day clinical assessments and smears, followed by an additional four weeks with weekly smears until eight weeks from the time of challenge have passed. Immunized volunteers who have not exhibited signs or symptoms of malaria within eight weeks will be considered very unlikely to develop malaria. They will be further monitored as described for all volunteers in the next paragraph. Infectivity controls will be monitored in a similar fashion as described above.

Volunteers will come to clinic for a semi-closeout visit at 12 weeks (3 months) post challenge. Active clinical follow-up (meaning face-to-face visits, periodic safety labs, periodic malaria blood smears, and measurements of immunogenicity) to document the duration of the immune response as well as any adverse events will extend to week 12 post challenge. In addition, there will be two optional visits at 6 and 12 months post the second immunization dose for those volunteers willing to come back to the clinical trials center, in order to obtain blood for long term assessment of immune responses. Although these two blood draws are not required for meeting criteria for completing the active phase of the clinical trial, they will be carried out whenever possible.

If volunteers become blood smear positive at any point, they will be immediately treated with chloroquine (or another anti-malarial medication such as atovaquone/proguanil (Malarone™) if allergy or contraindication to chloroquine is noted i.e. G6PD deficiency) for three days (25 mg/kg body weight total dose; Adult dosing of: Chloroquine = Loading dose of 1000mg followed in 6 hours by 500 mg then 500mg again 24 and 48 hours later; Atovaquone/Proguanil = 1 gm/400 mg po qd for 3 days), as the 3D7 and NF54 challenge strains of *P. falciparum* are chloroquine-sensitive. A volunteer will be considered fully protected if parasites are never detected in the bloodstream during follow-up, and partially protected if the detection of parasitemia is delayed relative to control volunteers.

If for whatever reason, a volunteer decides or needs to withdraw from the study in the first 30 days after being challenged with malaria (including day of challenge) but before developing signs or symptoms of malaria and/or parasitemia, they will require treatment to prevent the possibility of developing a patent infection. If withdrawl is on the day of challenge or the day after challenge then primaquine (30 mg base; 2 tablets) will given po for 4 days beginning immediately plus standard prophylactic dosing of chloroquine at 500mg (300 mg base; 1 tablet) weekly for 4 weeks would be used. However, if withdrawl is between 2 and 30 days then the volunteer would receive only chloroquine as a treatment regimen (outlined in the previous paragraph) followed by the same 4 week prophylactic regimen for those withdrawing on the day of or day after challenge. There is a slight risk of the volunteer developing infection though this is not anticipated as the parasite is choloroquine sensitive and the treatment plus prophylactic regimen should cover even those who may have delayed patency for whatever reason.

Furthemore, all volunteers will be instructed to contact the study team any time, within a year, post challenge should they experience fever, as an extra precaution to determine if a blood smear needs be collected and checked. If volunteers leave the study prior to the passage of one year post challenge and move outside of the Washington, D.C. area, they will be instructed to inform their medical care providers that they have been exposed to malaria and must have a blood smear checked whenever they develop a fever. In addition, they should request that their medical care providers contact the study physicians for advice concerning the diagnosis and treatment of malaria infection. If they leave the study prior to the passage of one year post challenge but remain in the area, they will be instructed to contact the study team whenever they have a fever within one year after a challenge so that a blood smear can be checked and treatment can be provided if indicated.

If for some reason no volunteers become infected, including the infectivity controls, indicating a failed challenge, a repeat challenge with a new batch of mosquitoes will have to be considered versus not pursuing another challenge. It must be noted that this has never occurred in the collective history of sporozoite challenges at NMRC or WRAIR and trying to determine ahead of time what the immediate course of action would be is impossible as the scenarios are endless. However, if this were to happen an emergency meeting of the investigators, associates and medical monitor would be called and some of the factors that would be considered include: causes for the failure, volunteer numbers and compliance/time frame for a possible repeat of the challenge as well as insectary issues. Regardless, the utmost concern would be for safety considerations for the volunteers. Infectivity controls would be offered the option to participate in a repeat challenge if this option was decided on with the plan for recruiting more volunteers to serve as infectivity controls to replace those that opted out of re-challenge.

### Treatment of Malaria for Infected Volunteers

Volunteers who develop blood stage malaria during follow-up will be treated with standard therapy using chloroquine or atovaquone/proguanil if cholorquine is contraindicated. If any volunteer has a history of inability to tolerate chloroquine (including allergic reaction), he/she would receive treatment with the alternative regimen of atovaquone/proguanil, to which the parasite is sensitive) using standard therapy. The infection will be treated as soon as parasites are identified by thick smear. All positive blood films will eventually be confirmed by at least one other microscopist with experience in the identification of malaria. Following treatment, daily blood films will continue until three consecutive smears are negative. A volunteer who develops malaria, is treated, and has 3 consecutive negative malaria smears, will not need to remain in the hotel. Blood films will be saved for later re-examination. CBC and serum chemistries will be performed, as clinically indicated when parasites are detected, and will be repeated as needed to confirm resolution of significant laboratory abnormalities.

It is anticipated that treatment will be curative; recrudescence has not occurred in any of the infected volunteers Nonetheless, once weekly evaluations will be performed for at least four weeks after treatment to evaluate the unlikely possibility of recrudescent infection. Treated infectivity control volunteers will be followed similarly. In addition, as with all subjects who have been challenged, treated volunteers will be advised to contact the study team, or to advise their personal physician of their participation in this malaria study, if fever, shaking chills, or other symptoms possibly related to malaria develop at any time within one year after challenge. In the unlikely event that malaria recurs, the individual will be retreated with chloroquine or an alternative regimen if appropriate.

To date, under our supervision, no severe, serious or life-threatening adverse events have been experienced by any of the hundreds of volunteers challenged in this manner. The most significant symptom experienced to date was orthostatic hypotension related to dehydration in a single volunteer who had been experiencing nausea and diarrhea two days following treatment for malaria infection; this volunteer received a liter of intravenous normal saline with resolution of her light headedness on standing. This was classified as a Grade 2 adverse event (“moderate”). Among volunteers who acquire malaria during challenge, approximately 20-40% have no symptoms and the remainder experience an illness akin to a mild to moderate influenza infection.

## Clinical Assessments

For safety purposes, a medical history, heath review, physical examination and laboratory examination will be performed at the time of screening. Health review, physical examination and laboratory examination will be repeated prior to immunization and at follow-up visits. Volunteers will also be required to complete temperature logs for the first week following immunization to increase accuracy in the reporting of fever. . Of note, volunteers will begin to fill out the temperature logs starting 7 days prior to immunization; this will allow for collection of baseline temperature data.

### Physical exams

At screening and at end of study a physical examination will be performed. In addition at any time a study physician feels it is clinically indicated a physical exam will be repeated.

At follow-up visits (after immunizations) abbreviated physical exams including vital signs, immunization site inspection, examination of eyes and respiratory system will be completed. In addition a review of medical history since previous visit, including any medication changes, will be conducted at each visit.

### Laboratories

Screening laboratories will include adenovirus 5 serology, HbsAg, anti-HCV antibody, and HIV ELISA. In addition at the time of screening, HLA typing will be completed and safety labs (as detailed below) will be collected.

Safety laboratories after screening will consist of CBC, Chemistries (including, Creatinine, AST, ALT,), UA, Beta-hCG measured from urine (for females) and will be completed as per protocol schedule. Any or all of these laboratories as well as any others deemed clinically indicated may be obtained on unscheduled days during the course of the study for the safety of volunteers.

Research serum will be collected and stored/archived for immunology (IFAT, anti-PfCSP and anti-AMA antibodies), and PBMCs will be isolated and stored. In addition, volunteers will be asked as part of the consent process if they will authorize any left over blood (in accordance with proper submission and approval guidelines) to be used for future research purposes not presently defined.

####

### Volunteer Temperature Logs

Volunteers will be required to complete daily temperature logs recording temperatures twice daily (using a thermometer that will be provided to each volunteer).These temperature logs will be maintained for a period of seven days before the first immunization to give volunteers an opportunity to get used to the recording and ask any questions that arise. This also provides the opportunity for the study team to distinguish between immunization related and other pre-existing baseline events. The diaries will be kept for seven days following injection and will be collected at the Day 7 post-vaccination clinical visit. For volunteers who receive two injections they will be required to keep a daily temperature log for seven days before and after each injection.

### Long Term Follow-up

Following active study participation and associated scheduled visits (lasting approximately one year post-challenge) immunized volunteers will be followed up for extended safety monitoring purposes by phone call or mail (electronic/U.S. Postal) twice per year (every six months +/- 1 month) for an additional four years using a report form (Appendix R). Volunteers will again be reminded that at any time during the first year that they develop a fever they should contact the study team as a precaution.

Volunteers will also be asked at the time of consent and again at the time of completion of active clinical follow-up to verify their own contact information and to provide contact information for at least one additional person in order to assist with locating the volunteer during the subsequent four years.

## Specimen Collection/Processing/Storage

### Blood specimens

Blood specimens will be collected by standard venipuncture for safety purposes and for research purposes. Repeated blood collections via venipuncture will be performed at various time points during the screening period, immunization period, and challenge period.

The amount of blood drawn will be tracked and will not exceed 525 mL over any eight week period whether drawn at a single time point or at multiple time points.

Other than for screening purposes and post challenge blood smears, blood will be drawn from control volunteers only if there is a clinical indication.

### HLA Typing

This will be done through contract agreement between NMRC (via Henry M. Jackson Foundation) and C. W. Bill Young/ DoD Marrow Donor Program (via Georgetown University). As part of screening a blood specimen for HLA typing will be collected, labeled using a numeric code and delivered to the lab. The HLA typing will be completed and the results will be sent via fax to the NMRC study team using the clinical trials office area in BLDG 141 on the Bethesda campus.

## Concomitant Medications

Any pre-existing conditions that require routine or intermittent medications should be discussed at the time of screening and a study physician will determine if participation is safe and will not interfere with any data being collected. If participation is permitted, the concomitant medication(s) will be recorded. Any new medications required during the course of participation in the study must be discussed with the study team prior to dosing to insure both safety of the volunteer and integrity of the data being collected. Information regarding all OTC and/or prescription medications taken will be solicited and recorded at each scheduled study visit.

## Withdrawal / Drop-outs

Volunteers will be reminded that participation is completely voluntary. Volunteers in all groups may decide not to participate or withdraw consent for continued participation at any time without penalty or losing any benefits to which they are entitled.

However, depending upon the time point in the study, sudden withdrawal without medical follow-up could be unsafe. For example, clinical follow-up after a challenge (to ensure proper diagnosis and medical treatment if a volunteer develops malaria) is important to insure the safety of the volunteer, even if they no longer consent to have their information used for research purposes. Therefore volunteers who withdraw from the study may be asked to complete clinical follow-up visits and may require anti-malaria medication treatment for safety purposes.

## Study Termination

The study team may terminate the study, or the participation of a particular volunteer, with or without the volunteer’s agreement if the study team believes it is in the volunteer’s best interest. For example volunteers could be discontinued if they develop a health condition that would make continued participation dangerous in the opinion of the principal investigator or medical monitor. A volunteer’s participation may also be terminated for failure to comply with the procedures or instructions provided by the study team.

Finally it is also understood by the study team, and will be explained to each volunteer, that the NNMC IRB, as the oversight IRB, the NMRC IRB, the Department of the Navy Bureau of Medicine and Surgery (BUMED), or the Sponsor (USAMMDA) have the right to end this research study at any time.

However, depending upon time point in the study, sudden termination by the investigators or oversight boards without providing medical follow-up could be unsafe just as it is for voluntary withdrawal. For example, clinical follow-up after a challenge (to ensure proper diagnosis and medical treatment if a volunteer develops malaria) is important to insure the safety of the volunteer, even if they no longer consent to have their information used for research purposes. Therefore if either the study, or a volunteer’s participation, is terminated the participant(s) may be asked to complete clinical follow-up visits or anti-malaria medication treatment for safety purposes.

# Study Product

Two replication-deficient adenovirus vectors expressing *P. falciparum* CSP or *P. falciparum* AMA1 have been generated. GenVec’s designation for these constructs are Adt.PfCSP.11D (NMRC designator: NMRC-MV-Ad-PfC) and Adt.PfAMA1.11D (NMRC designator: NMRC-MV-Ad-PfA).

The GV11 adenovirus backbone was chosen for malaria vaccine vector generation to provide increased transgene capacity and to reduce the risk of replication competent adenovirus (RCA) appearing via recombination events or via mutation during manufacturing.

The *P. falciparum* genes in the two constructs are inserted into the E1 region, with transcription initiated by a human cytomegalovirus (CMV) immediate-early promoter and terminated by a SV40 polyadenylation sequence. Adt.PfCSP.11D contains a synthetic, codon-optimized *P. falciparum* (3D7 strain) CSP gene, and Adf.PfAMA1.11D contains a synthetic, codon-optimized *P. falciparum* (3D7 strain) AMA1 gene.

The NMRC-M3V-Ad-PfCA Vaccine is formulated in a buffered saline solution called Final Formulation Buffer.

## Dose Groups

**Part A (Dose-escalation):**

Group 1 (NMRC-M3V-Ad-PfCA): 1 x 10^10^ pu each construct, 2 x 10^10^ pu total dose

Group 2 (NMRC-M3V-Ad-PfCA): 5 x 10^10^ pu each construct, 1 x 10^11^ pu total dose (five-fold increase)

**Part B (Regimen-comparison):**

Group 3 (NMRC-M3V-Ad-PfCA): 1x 10^10^ each per construct, 2 x 10^10^* pu total dose (two antigens)

Group 4 (NMRC-MV-Ad-PfC) and 5(NMRC-MV-Ad-PfA): 1 x 10^10^ each per construct, 1 x 10^10^*pu total dose (one antigen only)

* Anticipated dose based on safety, tolerability and available immunogenicity data from Part A.

## Route of Administration

Antigens will be vialed separately, combined into a third vial at the time of administration (for the two antigen vaccine), and injected intramuscularly at one site in the deltoid muscle. For volunteers who receive two immunizations, both will be given in the same arm. The left arm will be used unless there is a strong preference by the volunteer, or a medical indication, to use the right arm. Vaccine preparation for administration will be performed by a pharmacist or appropriately trained personnel within four hours of administration. For a detailed review of vaccine preparation see SOP in Appendix B or in Section 6.2 of the Investigator’s Brochure.

# Adverse Experiences

The recording of adverse events is an important aspect of study documentation. It is the responsibility of the investigator to document all adverse events according to the detailed guidelines set out below. The subjects will be instructed to contact the investigators immediately should they manifest any signs or symptoms they perceive as concerning.

## Adverse event definition

An adverse event includes any noxious, pathological or unintended change in anatomical, physiological or metabolic functions as indicated by physical signs, symptoms and/or laboratory values occurring in any phase of the clinical study whether or not considered to be associated with the study vaccine or other study procedure. This includes an exacerbation of pre-existing conditions or events, intercurrent illnesses, or vaccine or drug interaction. Anticipated day-to-day fluctuations of pre-existing conditions which do not represent a clinically significant exacerbation need not be considered adverse events. Discrete episodes of chronic conditions occurring during a study period will be reported as adverse events in order to assess changes in frequency or severity.

Adverse events recorded after challenge in individuals who become parasitemic will be considered most likely related to malaria infection and the relationship to vaccine will be recorded as “unrelated”.

## Reporting Procedures for Adverse Experiences

Pre-existing conditions or signs and/or symptoms (including any that are not recognized at study entry but are recognized during the study period) will be recorded on the Medical History form.

All adverse experiences will be described on the case report form using standard medical terminology in order to avoid the use of ambiguous or colloquial expressions. The investigator will evaluate all adverse experiences as to their severity and relationship to the investigational product, and will report outcome and action taken.

The period for reporting of solicited adverse experiences includes 14 days following each immunization for non-serious AEs and from the time of first immunization through the end of active study participation (1 year) SAEs (the SMC report on AE’s prepared following the immunizations in Part A will include solicited AE’s through 14 days post immunization of Group 2).

At each visit/assessment, all adverse events either observed or reported by the subject spontaneously or in response to a direct question will be evaluated. The nature of each event, date and time (where appropriate) of onset, outcome, intensity and relationship to immunization will be established and details of corrective action taken will be documented. As a consistent method of soliciting adverse events, the subject will be asked a non-leading question such as: "Have you felt different in any way since receiving the vaccine or since the previous visit?"

Adverse events already documented in the CRF, i.e. at a previous assessment, and designated as ‘ongoing’ will be reviewed at subsequent visits. If these have resolved, the documentation in the CRF will be completed. If an adverse event increases in frequency or intensity during a study period, a new record of the event will be started.

### Solicited Adverse Events

Solicited local and general adverse events

|  | **Adverse events** |
| --- | --- |
| **Local (injection site)** | Pain at the injection site |
|  | Swelling at the injection site |
|  | Redness at the injection site |
|  | Limitation in arm motion |
|  | Tenderness |
|  | Regional Adenopathy |
|  | Induration |
| **General** | Fever* (Oral Temperature) |
|  | Chills |
|  | Gastrointestinal (Nausea/vomiting) |
|  | Headache |
|  | Malaise |
|  | Myalgia |
|  | Fatigue |
|  | Joint pain |
|  | Diarrhea |
|  | Dysuria |
|  | Congestion |
|  | Conjunctivitis/pink eye (eye pain/irritiation) |
|  | Cough |
|  | Coryza |
|  | Pharyngitis |
|  | Urinary frequency |
|  | Hematuria |

*Fever is defined as oral temperature ≥ 38°C or 100.4°F

### Unsolicited adverse events

Space on the source documents and CRF will be allocated for the recording of unsolicited symptoms. Unsolicited symptoms are adverse events reported by the subjects that are different from those solicited.

## Serious Adverse Experiences (Events)

A *serious adverse experience (SAE)* may be defined as an adverse experience that:

- results in persistent or significant disability/incapacity;
- results in or prolongs in-patient hospitalization;
- results in congenital anomaly/birth defect;
- is life-threatening -- defined as an event in which there is, in the judgment of the investigator, an immediate risk of death. This does not include an AE that, had it occurred in a more serious form, might have caused death;
- is fatal.
- important medical events that may not result in death, be life-threatening, or require hospitalization may be considered serious when, based upon appropriate medical judgment, they require medical or surgical intervention to prevent one of the outcomes listed in this definition.

The investigator will assess all **serious** AEs as being either **related** (including definitely, probably or possibly) or **unrelated** to the experimental product or procedure.

**The principal investigator will forward SAE information to the medical monitor, the Sponsor, the NNMC IRB, the oversight IRB, the NMRC IRB and to the USAMRMC HSRRB as soon as possible.** The Sponsor will be responsible for reporting fatal or life-threatening adverse events to the FDA within seven calendar days, and other SAEs within 15 calendar days.

Specific guidelines for reporting to USAMRMC HSRRB are as follows: *Unanticipated problems involving risk to subjects or others, serious adverse events related to participation in the study, and all subject deaths should be promptly reported by telephone (301-619-2165), by e-mail (hsrrb@amedd.army.mil), or by facsimile (301-619-7803) to the U.S. Army Medical Research and Materiel Command, Human Subjects Research Review Board (HSRRB). A complete written report should follow the initial notification. In addition to the methods above, the complete report can be sent to the U.S. Army Medical Research and Materiel Command, ATTN: MCMR-ZB-PH, 504 Scott Street, Fort Detrick, Maryland 21702-5012.*

## Unexpected Adverse Experiences

An unexpected adverse event is any adverse experience, the specificity or severity of which is not consistent with the current investigator brochure.

**ADVERSE EXPERIENCES THAT ARE BOTH SERIOUS AND UNEXPECTED WILL BE IMMEDIATELY REPORTED BY TELEPHONE TO THE MEDICAL MONITOR. IN ADDITION, INFORMATION WILL THEN BE SENT BY FACSIMILIE TO THE NNMC IRB CHAIR. A WRITTEN REPORT WILL FOLLOW THE INITIAL TELEPHONE CALL WITHIN THREE WORKING DAYS TO THE MEDICAL MONITOR, IRB CHAIRs USAMRMC HSRRB AND SPONSOR. THE PI WILL ADVISE THE SPONSOR OF ADVERSE EVENTS DEEMED BY THE MEDICAL MONITOR THAT REQUIRE IMMEDIATE REPORTING TO THE FDA.**

The Medical Monitor will review all serious and unexpected adverse events and provide an unbiased written report of the event within ten calendar days of the initial report to the PI, relevant IRBs, Sponsor and USAMRMC HSRRB. At a minimum, the Medical Monitor should comment on the outcomes of the adverse event and the relationship of the adverse event to the test article. The Medical Monitor will indicate whether he concurs with the details of the report provided by the study investigator to the relevant IRBs, USAMRMC HSRRB and the Sponsor. See previous section for specific guidelines for reporting to USAMRMC HSRRB.

## Classification of Adverse Experiences

### Severity/Intensity

Severity/Intensity of AEs will be assessed by the investigator. A 0 to 4 grading scale will be used. The following general guidelines assist with the classification of AE’s:

Grade 0 = Minimal: is a barely noticeable discomfort and does not interfere with the subject's normal function. The AE resolves spontaneously.

Grade 1 = Mild: is a noticeable discomfort that does not interfere in a significant manner with the subject's normal daily activities. The AE resolves spontaneously, or may require minimal therapeutic intervention.

Grade 2 = Moderate: produces limited impairment of function, requires therapeutic intervention, but does not prevent normal daily activities once treated.

Grade 3 = Severe: results in a marked impairment of function, requires therapeutic intervention and prevents normal daily activities even with treatment.

Grade 4 = Serious: defined in preceding section, 12.3.

Normally, the intensity recorded for any adverse event will be the highest intensity in a 24-hour period. The 24-hour period will be a calendar day with the division between one day and the next occurring during sleep (i.e., pain experienced prior to falling asleep is attributed to the previous day even if occurring past midnight).

Specific AE’s defined as potential side effects for this study, including laboratory abnormalities, are listed in the following table along with the defined criteria that will be used for grading their severity / intensity.

**Intensity grading of local solicited adverse events**

| **Adverse event** | **Intensity grade** | **Intensity** |
| --- | --- | --- |
| Pain at injection site | 0 | Absent |
|  | 1 | Painful on touch |
|  | 2 | Painful when limb is moved |
|  | 3 | Pain that prevents normal everyday activities |
| Redness at injection site | 0 | 0 – 24 mm without necrosis |
|  | 1 | 25 - 50 mm without necrosis |
|  | 2 | 51 - 100 mm without necrosis |
|  | 3 | > 100 mm without necrosis |
| Swelling at injection site | 0 | 0 – 24 mm without necrosis |
|  | 1 | 25 - 50 mm without necrosis |
|  | 2 | 51 - 100 mm without necrosis |
|  | 3 | > 100 mm without necrosis |
| Induration | 0 | 0 – 24 mm without necrosis |
|  | 1 | 25 - 50 mm without necrosis |
|  | 2 | 51 - 100 mm without necrosis |
|  | 3 | > 100 mm without necrosis |

Swelling will also be graded according to the functional scale above. Per CBER guidelines and unless otherwise specified, Grade 4 would be those that require an ER visit or hospitalization and/or necrosis at the local injection site.

Intensity grading of general solicited adverse events

| **Adverse event** | **Intensity grade** | **Intensity** |
| --- | --- | --- |
| Fever | 0 | < 38°C or 100.4°F |
|  | 1 | 38.0 – 38.4 °C or 100.4 – 101.1 °F |
|  | 2 | > 38.5 to 38.9 °C or 101.2 -  102.0 °F |
|  | 3 | > 39 – 40 °C or 102.1 – 104 °F |
| Tachycardia | 0 | < 101 beats per minute |
|  | 1 | 101 – 115 beats per minute |
|  | 2 | 116 – 130 beats per minute |
|  | 3 | > 130 beats per minute |
| Bradycardia | 0 | > 50 beats per minute |
|  | 1 | 50 – 54 beats per minute |
|  | 2 | 45 – 49 beats per minute |
|  | 3 | < 45 beats per minute |
| Hypertension (systolic) | 0 | < 141 mm Hg |
|  | 1 | 141 – 150 mm Hg |
|  | 2 | 151 – 155 mm Hg |
|  | 3 | > 155 mm Hg |
| Hypertension (diastolic) | 0 | < 91 mm Hg |
|  | 1 | 91 – 95 mm Hg |
|  | 2 | 96 – 100 mm Hg |
|  | 3 | > 100 mm Hg |
| Hypotension (systolic) | 0 | < 85 mm Hg |
|  | 1 | 85 – 89 mm Hg |
|  | 2 | 80 – 84 mm Hg |
|  | 3 | < 80 mm Hg |
| Respiratory Rate | 0 | < 21 breaths per minute |
|  | 1 | 21 – 24 breaths per minute |
|  | 2 | 24 – 27 breaths per minute |
|  | 3 | > 27 breaths per minute not requiring intubation |
| Headache | 0 | Normal |
|  | 1 | Easily tolerated, no interference with activity |
|  | 2 | Interference with activity or repeated use of non-narcotic pain reliever |
|  | 3 | Prevents daily activity or repeated use of narcotic pain reliever |
| Fatigue | 0 | Normal |
|  | 1 | Easily tolerated, no interference with activity |
|  | 2 | Interferes with normal activity |
|  | 3 | Prevents normal activity |
| Gastrointestinal | 0 | Normal |
| (*Nausea/Vomiting)* | 1 | Easily tolerated or 1-2 episodes per 24 hours |
|  | 2 | Interferes with normal activity >2 episodes per 24 hours |
|  | 3 | Prevents normal activity, requires outpatient IV hydration. |
| (*Diarrhea)* | 0 | Normal |
|  | 1 | 2-3 loose stools or <400gm per 24 hours |
|  | 2 | 4-5 loose stools or 400-800 gm per 24 hours |
|  | 3 | 6 or more watery stools or > 800gm per 24 hours or requires outpatient IV hydration |
| Malaise | 0 | Normal |
|  | 1 | Easily tolerated, no interference with activity |
|  | 2 | Interferes with normal activity |
|  | 3 | Prevents normal activity |
| Myalgia | 0 | Normal |
|  | 1 | Easily tolerated, no interference with activity |
|  | 2 | Interferes with normal activity |
|  | 3 | Prevents normal activity |
| Joint pain | 0 | Normal |
|  | 1 | Easily tolerated, no interference with activity |
|  | 2 | Interferes with normal activity |
|  | 3 | Prevents normal activity |

**Per CBER guidelines Grade 4 would be any value beyond the highest or lowest value of Grade 3 as it relates to the specific test value and/or is life threatening so as to require and ER visit or hospitalization.**

Intensity grading of laboratory abnormalities

| **Adverse event** | **Intensity grade** | **Intensity** |
| --- | --- | --- |
| WBC Increase | 0 | Normal (3,500-11,000 cell/mm^3^) |
| Grade 1 | 1 | 11,000 – 15,000 cell/mm^3^ |
| Grade 2 | 2 | 15,000 – 20,000 cell/mm^3^ |
| Grade 3 | 3 | 20,000 – 25,000 cell/mm^3^ |
| WBC Decrease | 0 | Normal (3,500-11,000 cell/mm^3^) |
| Grade 1 | 1 | 2,500 – 3,500 cell/mm^3^ |
| Grade 2 | 2 | 1,500 – 2,499 cell/mm^3^ |
| Grade 3 | 3 | 1,000 – 1,499 cell/mm^3^ |
| Lymphocyte Decrease | 0 | Normal (>1,000) cell/mm^3^) |
| Grade 1 | 1 | 750 – 1,000 cell/mm^3^ |
| Grade 2 | 2 | 500 – 749 cell/mm^3^ |
| Grade 3 | 3 | 250 – 499 cell/mm^3^ |
| Neutrophil Decrease | 0 | Normal (> 2,000 cell/mm^3^) |
| Grade 1 | 1 | 1,500 – 2,000 cell/mm^3^ |
| Grade 2 | 2 | 1,000 – 1,499 cell/mm^3^ |
| Grade 3 | 3 | 500 – 999 cell/mm^3^ |
| Eosinophil Increase | 0 | Normal (< 650 cell/mm^3^) |
| Grade 1 | 1 | 650 – 1,500 cell/mm^3^ |
| Grade 2 | 2 | 1,501 – 5,000 cell/mm^3^ |
| Grade 3 | 3 | >5,000 cell/mm^3^ |
| Hemoglobin (Males) | 0 | Normal (13.2 – 17.1 gm/dl) |
| Grade 1 | 1 | 12.5 – 13.1 gm/dl |
| Grade 2 | 2 | 10.5 – 12.4 gm/dl |
| Grade 3 | 3 | 8.5 – 10.4 gm/dl |
| Hemoglobin (Females) | 0 | Normal (11.7 – 15.5 gm/dl) |
| Grade 1 | 1 | 11 – 11.6 gm/dl |
| Grade 2 | 2 | 10 – 11.5 |
| Grade 3 | 3 | 8.0 – 9.9 gm/dl |
| Platelets | 0 | Normal (>140,000) |
| Grade 1 | 1 | 125,000 – 140,000 |
| Grade 2 | 2 | 100,000 – 125,000 |
| Grade 3 | 3 | 25,000 – 99,000 |
| Creatinine | 0 | Normal Female 0.4 – 1.1 mg/dL  Normal Male .6-1.2 mg/dL |
| Grade 1 | 1 | Female 1.1 - 1.9 mg/dL  Male 1.2 -1.9 mg/dL |
| Grade 2 | 2 | 1.9 - 2.2 mg/dL |
| Grade 3 | 3 | 2.2 – 2.5 mg/dL |
| Blood Urea Nitrogen (BUN) | 0 | < 23 mg/dL |
| Grade 1 | 1 | 23 – 26 mg/dL |
| Grade 2 | 2 | 27 – 31 mg/dL |
| Grade 3 | 3 | > 31 mg/dL |
| Alkaline Phosphatase | 0 | Normal |
| Grade 1 | 1 | 1.1 - 2.0 times upper limit of normal (ULN) |
| Grade 2 | 2 | 2.1 - 3.0 times ULN |
| Grade 3 | 3 | 3.0 - 10 times ULN |
| AST | 0 | Normal |
| Grade 1 | 1 | 1.1 - 2.5 times upper limit of normal (ULN) |
| Grade 2 | 2 | 2.6 – 5.0 times ULN |
| Grade 3 | 3 | 5.1 - 10 times ULN |
| ALT | 0 | Normal |
| Grade 1 | 1 | 1.1 - 2.5 times upper limit of normal (ULN) |
| Grade 2 | 2 | 2.6 – 5.0 times ULN |
| Grade 3 | 3 | 5.1 - 10 times ULN |
| Total Bilirubin – when accompanied by any  increase in Liver Function Test  increase by factor | 0 | Normal |
| Grade 1 | 1 | 1.1 – 1.25 times upper limit of normal (ULN) |
| Grade 2 | 2 | 1.26 – 1.5 times ULN |
| Grade 3 | 3 | 1.51 – 1.75 times ULN |
| Total Bilirubin – when Liver Function Test  is normal; increase by factor | 0 | Normal |
| Grade 1 | 1 | 1.1 - 1.5 times upper limit of normal (ULN) |
| Grade 2 | 2 | 1.6 – 2.0 times ULN |
| Grade 3 | 3 | 2.1 – 3.0 times ULN |
| Urinalysis | 0 | None detected |
| *(Protein)* | 1 | Trace |
|  | 2 | 1+ |
|  | 3 | 2+ |
| *(Glucose)* | 0 | None detected |
|  | 1 | Trace |
|  | 2 | 1+ |
|  | 3 | 2+ |
| *(Microscopic Blood)* | 0 | None detected |
|  | 1 | Trace |
|  | 2 | 1+ |
|  | 3 | 2+ |

**Per CBER guidelines Grade 4 would be any value beyond the highest or lowest value of Grade 3 as it relates to the specific test value and/or is life threatening so as to require and ER visit or hospitalization.**

### Relationship to participation in study

All adverse experiences that occur will be evaluated by the investigator and a determination will be made as to the likelihood that the adverse experience is related to a study procedure (e.g., venipuncture, immunization, malaria challenge, chloroquine administration) rather than to the vaccine test article. The relationship classification used refers to the relationship, in the investigator’s assessment, of the Adverse Experience TO THE VACCINE.

# Endpoints

Safety and tolerability results, starting from the day of immunization of Group 1 until 2 weeks after immunizing Group 2, will be reviewed by the SMC and a written report will be submitted to the Sponsor, USAMRMC ORP relevant IRBs, and FDA prior to beginning part B. Any comments and correspondence by the FDA regarding the transition from Part A to Part B will also be submitted to the same parties prior to beginning Part B. Immunogenicity assessment from Part A is not a prerequisite for continuation to Part B.

**Part A**:

Primary endpoints:

- Documentation of occurrence, severity and duration of vaccine related solicited symptoms over a 14-day follow-up period (day of vaccination and 13 subsequent days).
- Documentation of occurrence, severity and duration of vaccine related unsolicited symptoms, abnormal physical findings and abnormal laboratory values over a 30 day follow-up period (day of immunization and 29 subsequent days).
- Documentation of occurrence, severity and duration of vaccine related serious adverse events during the one year active study period as defined in IAW 21 CFR 312.32(a).

Secondary endpoints:

- CSP: Analysis of IFN-γ ELIspot against synthetic peptides derived from PfCSP using PBMCs collected at screening/pre-immunization, 10 & 28 days post immunization.
- AMA1: Analysis of ELISA (against recombinant PfAMA1 protein capture antigen) for sera/plasma collected at screening/pre-immunization, 10 & 28 days post immunization.

Tertiary endpoints:

- CSP: Analysis of ELISA (against recombinant CSP protein capture antigen) for sera/plasma collected at screening/pre-immunization, 10 & 28 days post immunization.
- AMA1: Analysis of IFN-γ ELIspot against synthetic peptides derived from PfAMA1 and/or recombinant PfAMA1 protein using PBMCs collected at screening/pre-immunization, 10 & 28 days post immunization.
- Analysis of growth inhibition assay (GIA) at screening/pre-immunization, 10 & 28 days post immunization.
- Analysis of anti-sporozoite immunofluorescence assay (IFA) titers at screening/pre-immunization, and 10 & 28 days post immunization.

**Part B**:

Primary endpoints:

- Documentation of occurrence, severity and duration of solicited symptoms over a 14-day follow-up period (day of vaccination and 13 subsequent days).
- Documentation of occurrence, severity and duration of unsolicited symptoms, abnormal physical findings and abnormal laboratory values over a 30 day follow-up period (day of immunization and 29 subsequent days).
- Documentation of occurrence, severity and duration of serious adverse events during the one year active study period, as defined in IAW 21 CFR 312.32(a).
- Determination of vaccine efficacy (development of parasitemia and time to development of parasitemia) after sporozoite challenge two to four weeks post-final immunization.

Secondary endpoints:

- CSP: Analysis of IFN-γ ELIspot against synthetic peptides derived from PfCSP using PBMCs collected at screening/pre-immunization, 28 days post first immunization, prior to second immunization, day of challenge (before challenge) and 28 days post challenge.
- AMA1: Analysis of ELISA (against recombinant PfAMA1 protein capture antigen) for sera/plasma collected at screening/pre-immunization, 28 days post first immunization, prior to second immunization, day of challenge (before challenge) and 28 days post challenge.

Tertiary endpoints:

- CSP: Analysis of ELISA (against rec. CSP protein capture antigen) for sera/plasma collected at screening/pre-immunization, 28 days post first immunization, prior to second immunization, day of challenge (before challenge) and 28 days post challenge.
- AMA1: Analysis of IFN-γ ELIspot against synthetic peptides derived from PfAMA1 and/or recombinant PfAMA1 protein using PBMCs collected at screening/pre-immunization, 28 days post first immunization, prior to second immunization, day of challenge (before challenge) and 28 days post challenge.
- Analysis of GIA at screening/pre-immunization, 28 days post immunization, day of challenge (before challenge) and 28 days post challenge. Analysis of anti-sporozoite immunofluorescence assay (IFA) titers at screening/pre-immunization, 28 days post first immunization, prior to second immunization, day of challenge (before challenge) and 28 days post challenge.

## Safety

During the immunization period, serial clinical laboratory assays and clinical evaluations will assess safety and tolerability as noted above. The Clinical Toxicity Criteria as defined in section 12.5.1 will be used to grade adverse event severity and adverse events will also be classified according to relationship to immunization (definitely related, probably related, possibly related or unrelated) and whether or not they are expected or unexpected. Serious adverse events will be defined and reported according to the requirements of the Code of Federal Regulations.

# Immunology Definitions

In order to perform immunogenicity assays, fresh heparinized blood and plasma/serum will be collected at screening/pre-immunization, 10 days post immunization , prior to second immunization, day of challenge (before challenge) and 28 days post challenge. At each draw for immunology labs, approximately 130 ml of whole blood will be collected for PBMCs that will be used fresh or frozen (stored in liquid nitrogen) for ELIspot and intracellular cytokine staining assays. Likewise, at each draw for immunology labs approximately 20 ml of blood for plasma/serum will be stored (at –70^O^C) frozen for IFAT and ELISA. This results in a total of 150 ml of blood drawn for each draw for immunogenicity testing.

Immunogenicity will be assessed by measuring antibodies and T cell responses. Antibody levels in sera will be assessed by IFAT against air-dried sporozoites and blood stage parasites as well as ELISA against recombinant proteins and/or synthetic peptides ten days and four weeks after each immunization. Antibody-mediated inhibition will also be measured by GIA using homologous 3D7 *P. falciparum* parasites in a 1-cycle static assay as correlate of the potential parasite-neutralizing activity of the antibodies *in vivo.*

Induction of antigen-specific T lymphocytes will be assessed by several methods. As soon as volunteers enroll in the study, approximately 130 mL of whole blood will be drawn for separation and freezing of cells for subsequent assessment of pre- and post-immunization T cell responses assessed simultaneously in blinded fashion. Prior to the first immunization and following each immunization, up to 130 mL of whole blood will be drawn for measuring T cell responses. PBMC will be stimulated *in vitro* with synthetic peptides based on *P. falciparum* antigens, and cytokines, including but not limited to IFN-gamma, will be evaluated by the ELIspot method and/or intracellular staining for cytokine coupled to FACs analysis. Two types of ELIspot assays may be performed: *ex vivo*, meaning a 36 hour culture period to assess effector cell populations, and “cultured” ELIspot, meaning a 7-14 day culture period to assess memory cell populations. The shorter incubation is appropriate for measuring the existing, circulating effector T cell population with specificities for the test antigen, while the longer incubation assesses the population of memory cells capable of proliferation after several days in the presence of antigen.

Each PBMC or serum/plasma sample will be assayed by ELIspot or ELISA assays in triplicate or quadruplicate. For each triplicate or quadruplicate, outliers will be rejected if any single triplicate (or quadruplicate) value contributes more than 50% of the standard deviation of the triplicate (or quadruplicate) and if its value is three-fold greater or less than the average of the remaining two (or three) values. After removing outliers, the mean spot forming cells (SFCs, ELIspot), OD (ELISA, at each serum dilution) or fluorescent signal (flow) obtained in negative control wells/tubes (PBS or malaria-naïve sera) will be subtracted from the value of each well/tube. Negative counts, if any are generated by this background subtraction, will be converted to zero. The mean and standard deviation of the test sample will then be calculated. Antibody levels will be log-transformed before analysis. Previous experience with ELIspot, ELISA, and flow-based assays suggests that the total counts will be normally distributed; if they are not, then non-parametric methods will be used for the comparisons.

For GIA, sera will be heat inactivated and dialyzed against culture media made pH 7.4 with sodium hydroxide instead of sodium bicarbonate (RPMI-NaOH). Sera (20% v/v) will be cultured 2 days with 0.2% initial parasitemia (trophozoites) 4% hematocrit in triplicate 150ul static cultures in 48-well plates. Cultures will be harvested and stained with Hoechst dye 33342 and the new trophozoites counted in 40,000 erythrocytes by flow cytometry. Growth inhibition will be calculated from final parasitemias as: Inhibition = (control-test)/control, where control is the final parasitemia with pre-immune serum. Selected positive sera and controls will be retested and titered out under 4 conditions:

3D7 static;

3D7 suspension

FVO static

FVO suspension.

(Using the heterologous FVO parasite in GIA will give an indication of possible cross-protection.)

Growth inhibition assays (GIA) will be reported in a tabular manner as the percent growth inhibition seen at a given serum dilution compared with a negative control serum, such as a pre-immune serum from the same volunteer.

Immunological outcomes expressed as means (e.g., magnitude of responses, titer of antibodies as determined by endpoint dilution, OD values, concentration of cytokines as determined by reference to positive standard controls) will be compared between groups using the Student’s *t*-test (two-tailed), paired if pre-immunization values are compared with post-immunization values, unpaired if comparisons are made between groups. Those outcomes expressed as proportions (prevalence of antibodies, frequency of cytokine responses, frequency of positive assays) will be compared between groups by χ^2^ test. In all analyses, *p* < 0.05 will be considered statistically significant. True difference in response proportions relative to negative controls will be also calculated (95% CI) for flow cytometry data: if the confidence interval is entirely above 0.05%, the response is positive; if the confidence interval is entirely below 0.05%, the response is negative; if the confidence interval overlaps with 0.05%, the response is indeterminate. Assay sensitivity, specificity, and positive and negative predictive values (+ 95% CI) (with respect to protective immunity) will be established using standard formula.

## Humoral Responses

For IFAT, seroconversion will be defined as a four-fold rise, as compared to the pre-immune serum, against intact *P. falciparum* sporozoites or blood stage parasites by IFAT. For ELISA, seroconversion will be defined as a mean optical density of ELISA assays (conducted in triplicate or quadruplicate) against recombinant protein or synthetic peptide antigens at a specified (e.g., 1:100 or 1:200) dilution of serum that is greater than the mean plus two standard deviations of the mean of triplicate pre-immunization serum at the same dilution. A serum will be considered to be GIA positive if, compared with a negative control serum, the growth inhibition value is 10% or more and with a two-tailed students’s t test p value of less than 0.05. Response to vaccinations will be calculated relative to each individual volunteer’s pre-immune results for each assay.

## Cellular Responses

A positive response for ELIspot will be defined as (1) a statistically significant difference between the number of spot forming cells in quadruplicate test wells and quadruplicate control wells, plus (2) at least a doubling of spot forming cells in test wells relative to control wells, plus (3) a difference of at least five spots between test and control wells. Samples will be considered positive by intracellular cytokine staining assays if (1) the response to test antigens is greater than 0.01%; (2) the response to test antigens is > two-fold background responses to the control protein and PBS-treated samples, and (3) there is a true difference in response proportions (95% confidence interval). For both assays, responses to vaccinations will be calculated relative to pre-immune specimens.

# Clinical Definitions

## Protective Efficacy

Protection will be defined as a statistically significant delay in the day of onset of parasitemia, or a significant difference in the number of volunteers becoming parasitemic, when vaccinees are compared to controls.

# Human Subjects Protection Considerations

## Risks of study participation

Risks associated with immunization include local inflammatory reactions, lymphadenitis, persistent pruritis, induration or sterile abcess at the injection site, or larger local reactions involving the whole forearm including regional lymphadenitis, and systemic reactions including fever, myalgia, nausea, fatigue, headache. Severe allergic reactions such as anaphylaxis are also possible. There is additionally the theoretical risk of adenovirus infection, although the vaccine vector is non-replicating. However, volunteers will we checked for symptoms of upper respiratory infection, gastroenteritis, urinary tract infection and conjunctivitis in order to monitor this possibility. Risks associated with challenge include allergic reactions to mosquito bites and the development of malaria infection.

Some new data from a different study using Ad5 vaccine as the carrier for synthetic HIV genes was recently presented. It showed that there were more HIV infections in volunteers with pre-existing antibodies to Ad5 who were immunized with the vaccine than in volunteers who received placebo. The overall risk for acquiring HIV did not go up for those who were vaccinated and had pre-exisiting Ad5 antibodies, but rather the risk appeared to decrease for those with pre-existing Ad5 antibodies in the placebo group. At this time it appears to be due to the affect of the Ad5 backbone and not the HIV inserts. However, this study was with a different Ad5 vaccine for a different disease in a population at very high risk for HIV so it is to early to draw conclusions about this finding. The reasons for this result are still being studied. The details of this have been added as a fact sheet to Appendix A. However, until this early data can be studied further, there is the possibility that people with pre-existing antibodies to Ad5 who receive an Ad5 vaccine may be at higher risk of becoming infected with HIV upon exposure than those with the same level of pre-existing Ad5 antibodies who are not immunized with an Ad5 vaccine.

Additional risks include possible side effects of chloroquine (and of Malarone if this alternative drug is used) which include nausea, vomiting, diarrhea, abdominal pain, dizziness, headaches, blurred vision, pruritus, tinnitus, and photosensitivity. The study team will discuss these medications and their possible side effects in detail prior to initiation of treatment for volunteers who are infected with malaria.

There is also the possibility of complications of malaria, which are seen during naturally acquired malaria when diagnosis and treatment are delayed and high levels of parasitemia develop. Under the carefully controlled conditions of this study, the chance of complications is small and the risk of death from malaria infection is very small.

In the course of research experience with malaria and with approximately 1500 malaria challenges, we are aware of the following events,

- One person at another institution died from complications that were possibly related to malaria infection or treatment (a possible idiosyncratic reaction to quinine).
- Two people have experienced heart related complications. Both volunteers were participating in separate malaria-related trials conducted in the Netherlands. One was a 20 year old female who was challenged with malaria and experienced chest pain that required hospitalization several days for a presumed mild heart attach, after her malaria infection was treated. We do not know the cause of this event and therefore can not predict what factors might have placed her at increased risk for this event. Both the vaccine she received and the drug used to treat her malaria infection were different than those used in this trial.
- The other heart related complication occurred in a 39-year-old male who had a heart attack nine days after his malaria challenge and one day after receiving anti-malaria treatment. Later studies of his heart suggested that he had unrecognized severe heart disease

In previous malaria vaccine trials conducted by our program no serious adverse events have occurred and no hospitalizations have been required, related to the malaria challenge. Transient abnormalities, e.g. fever, headache, myalgias, shaking chills, abdominal discomfort, nausea, vomiting, dehydration, mild anemia, leukopenia, thrombocytopenia, hepato/splenomegaly, hepatic tenderness and fatigue, are expected consequences of malaria. In uncontrolled circumstances, malaria infections can lead to kidney, liver, heart, or brain injury (seizures, coma) and death.

Other remotely possible risks include a systemic allergic reaction to mosquito bites or anti-malarial drugs and the chance that the mosquitoes may transmit another infectious agent.

### Risks for Active Duty Military Volunteer Deployment

### The described risks may pose particular concern for supervisors of active duty personnel who volunteer for this study in terms of affecting deployment. While the described side effects of the vaccine may temporarily affect an active duty volunteer’s work performance, in no way does participation in this trial, before or after immunization, impair an active duty members ability to deploy or receive vaccines necessary for deployment. The member would simply be withdrawn from the study if unable to complete the study requirements due to deployment, though data collected up until that time would be used.

### Similarly for malaria challenge, an active duty member who volunteers for Part B of this study and is challenged could have their work performance temporarily affected if they contract malaria, however, this would not affect their overall ability to deploy as they are closely monitored and treated at the first sign of parasitemia which only requires a few days. Likewise, they can be treated to prevent malaria after challenge but before expected onset of malaria symptoms or if they withdraw before 30 days after challenge (should there be apparent protection from the vaccine). They would, however, still be unable to donate blood for 3 years per the American Red Cross guidelines,

### Though not anticipated, there is the remote risk of an unforeseen event that could temporarily or permanently affect an active duty member’s deployment status such as death or impairment from systemic allergic responses to the vaccine, mosquito bites or anit-malarial drugs.

## Precautions to Minimize Risk of Study participation

### Precautions To Minimize Risks Associated With Blood Drawing

Throughout this study, the amount of blood collected will be no more than 35 tablespoons (525 mLs) in any 8-week period (the amount of blood allowed to be drawn under the American Association of Blood Banks standards).

Blood tests including blood counts will be performed regularly during the study (including at the beginning and at the end) to assess for anemia. If significant anemia develops, volunteers may be withdrawn from the study and will be treated by the study doctor if needed in coordination with the volunteer’s personal doctor if they wish.

Volunteers will be screened for antibodies to HIV, hepatitis B and hepatitis C for both assessment of suitability of study participation as well as for protection of laboratory and health care personnel. Standard procedures will be followed for handling blood and body fluid specimens.

### Precautions To Minimize Risks Associated With Immunization

Medication and equipment to treat allergic reactions will be available and a study doctor will be present to monitor the immunization process and the volunteers for at least 30 minutes after the immunization.

Precautions to becoming infected with HIV involve avoiding unsafe sex (including unprotected vaginal, oral, or anal sex; multiple sexual partners; sexual contact with commercial sex workers; sexual contact with persons you do not know well; sexual contact with persons positive for HIV or of unknown HIV status; incorrect condom use; or sexual contact while under the influence of drugs or alcohol), intravenous drug use or other contact with HIV infected blood or fluids.

### Precautions To Minimize Risks Associated With Challenge

Since 1985 [107, 108] sporozoite challenge of volunteers has entered a new era. Previously infected volunteers were the source of parasites for infection of mosquitoes, or direct challenge with infected erythrocytes. In the 1980s we began using cultured parasites as the source of gametocytes for infecting mosquitoes. This means that we have extremely well-defined reagents that are consistently used. Our experience through 1992 with 118 volunteers who have been experimentally infected using a *P. falciparum* challenge model has been published [105, 106].

The mosquitoes used in this study for challenge are raised in a supervised laboratory at NMRC in Silver Spring, MD. In the laboratory, the mosquitoes used for challenges are infected with defined strains of malaria by feeding on malaria-infected human blood. To minimize the risk of disease transmission the following precautions are taken:

1. The malaria-infected blood is taken from humans who are not at risk of having other infections.
2. The malaria-infected blood is tested for syphilis, hepatitis B and C viruses and the HIV virus that causes AIDS before the mosquitoes are allowed to feed on it.
3. The length of time from when the mosquitoes feed on the donated blood until they feed on the volunteer is 16-18 days to further decrease any chances of transmitting an adventitious agent. Specifically, there has been no reported transmission of human viruses, including HIV and hepatitis, using this system. In addition, mosquitoes are known to digest hepatitis B surface antigen within 2-3 days.

Volunteers will be monitored closely, especially following the challenge with viable sporozoites. As soon as malaria infection is documented, volunteers will be treated as described in the previous section. Prompt treatment eliminates the risk of developing a serious complication due to the malaria infection. There are no strains of malaria that cannot be effectively treated if parasitemia is diagnosed promptly.

The risk of accidentally transmitting malaria to a person in the community will be negligible; infected mosquitoes will be restricted to the insectary or, if being transported, will be kept inside double closed contains, with the outer container a locked metal box. Infections in volunteers will be treated promptly, before gametocytes can develop.

### Precautions To Minimize Risks To Deployment for Active Duty Military Volunteerst

As stated previously, those active duty volunteers who must deploy after receiving the vaccine and cannot complete the requirements of the study would be withdrawn from the study, but still be able to deploy and receive vaccines for deployment.. However, every attempt would be made to continue follow-up for safety monitoring purposes by the study team if the member still wanted to participate and this was allowed by their deployment schedule and supervisor. Additionally, any required follow-up due to adverse events experienced by the active duty volunteer would be coordinated through the member’s command and/or deployment unit and the NMRC clinical trials team.

Similarly for malaria challenge, any active duty volunteer needing to deploy at any point after malaria challenge would be treated appropriately according to standard practice/established SOPs to either treat or prevent the onset of malaria symptoms if they need to deploy/withdraw within 30 days after challenge. This would not necessarily prevent deployment but may require the member to take medication during the first few days to weeks of deployment depending on the time of withdrawal after challenge. Enough medication would be provided by the study team to complete treatment as well as the requisite number of smears would be completed to assure clearance of parasitemia (if being treated for parasitemia/malaria symptoms). Any follow-up labs would be coordinated through the member’s command and/or deployment unit and the NMRC clinical trials team. In addition, the member would be educated on the signs and symptoms of malaria and the need to be seen and notify treating healthcare providers of their participation in a malarial challenge. Contact information for the study team would be re-issued as well as re-education concerning their inability to donate blood for the following 3 years.

Finally, in order to assure communication with active duty volunteer’s respective commands, all active duty member volunteers are required to obtain approval from their supervisors to participate in this trial once found eligible after the initial screening visit (Appendices H, I & FF). Further, participation in the study would not be allowed without such documentation regardless of eligibility status and the member would be withdrawn by the study team if unable to produce such documentation by the time of vaccination.

# Data Management and Analysis

## Data Collection and Storage

All documents requiring signatures will be paper and will be stored as part of the volunteers research chart which will be kept in a secure file cabinet in a locked office at the NMRC Clinical Trials Center, Building 141 of the NNMC campus. In addition standardized forms will be used for data collection. Data will be collected primarily through the use of paper copies of the data collection forms; however electronic versions will be available for recording and storage of data for later analysis.

The data will be entered into a secure and level locked database. Each user of the database will have a password and designated level of access. Data viewing will be possible as a separate option from either data entry or data modification. Ten percent of all data entered into the database will randomly checked for accuracy. To the extent possible data will be entered through selections made on drop down menus to reduce the number of typographical errors made. For data requiring direct data entry parameters will be set with alerts to notify out of range values.

Filemaker (the proposed database) is compatible with numerous statistical analysis packages (including SPSS) as well as Microsoft products. Thus data may be exported for statistical analysis and the need for duplicated efforts of entry will be reduced, thereby reducing the rate of errors. In addition any laboratory data that is provided in a Microsoft format can be imported into the database, again decreasing the change for entry error.

All participant records and consent forms obtained during the protocol will be maintained and stored in a secured, locked location at the NMRC Malaria Department’s clinical trial offices. As required, this original protocol, all reports, consent forms, questionnaires and other pertinent protocol records will be kept in the Office of Research Administration’s Command archives, Naval Medical Research Center, Silver Spring, MD.

It is the policy that data sheets of USAMRMC that data sheets are to be completed on all volunteers participating in research for entry into the U.S. Army Medical Research and Materiel Command Volunteer Registry Database. The information to be entered into this confidential database includes name, address, social security number, study name and dates. The intent of this database is twofold: first to readily answer questions concerning an individual’s participation in research sponsored by USAMRMC; and secondly, to ensure that the USAMRMC can exercise its obligation to ensure research volunteers are adequately warned (duty to warn) of risks and to provide new information as it becomes available. The information will be stored at the USAMRMC for a minimum of 75 years.

## Statistical Analysis Plan

### Hypotheses to be tested

1. The NMRC-M3V-Ad-PfCA vaccine is safe, well-tolerated and immunogenic.

2. The two antigen vaccine demonstrates no significant interference between the components as measured by immunogenicity.

### Analysis of Safety and Tolerability

The vaccine will be considered safe and well-tolerated if there are no severe or serious adverse events related to vaccine administration.

To analyze safety and tolerability, the overall percentage of subjects with at least one local adverse event (solicited and unsolicited), and with at least one general adverse event (solicited and unsolicited), during the fourteen-day follow-up period after immunization, will be tabulated. In addition, the incidence, intensity and relationship of individual solicited symptoms over the 14-day follow-up period will be calculated per group and vaccine dose. The number of subjects with at least one report of unsolicited adverse event reported up to 30 days after immunization, will also be tabulated per group and vaccine dose. The intensity and relationship to immunization of the unsolicited symptoms reported will also be assessed.

### Analysis of Immunogenicity

Immunogenicity will be assessed by the number of responders, the magnitude of response, and the number of positive assays, as described in Section 14.

### Analysis of Efficacy

If immunized volunteers are completely protected against malaria infection, protective efficacy will be calculated as the number of malaria positive volunteers divided by the number challenged in the experimental group, relative to the control group:

(# positive in experimental group X)/(# challenged in experimental group X)

Efficacy = [1- --------------------------------------------------------------------------------------------] **_X 100_**

(# positive in control group)/(# challenged in control group)

If volunteers are not completely protected against malaria infection, immunized and control volunteers will be ranked according to time of onset of parasitemia and a non-parametric rank-order statistical test performed to look for delays in parasitemia induced by vaccination (see below).

### Statistical Tests

Measurements with normal distributions expressed as means of continuous data (e.g., magnitude of responses) will be assessed using the Student’s t test (two-tailed), paired if pre-immunization values are compared with post immunization values, and unpaired if comparisons are made between groups. For discrete variables with normal distributions (e.g. number of responders, the number of positive assays, the number of individuals protected against challenge), the chi-squared test or Fisher’s exact test will be used (two-tailed, uncorrected for chi-squared), except when the cell value is five or less, in which case only Fisher’s exact test will be used (two-tailed). For days to parasitemia, volunteers will be rank-ordered and a non-parametric test suitable for unpaired groups (e.g., Mann Whitney) will be employed (one-tailed if comparing a vaccine group to controls, two-tailed if comparing one vaccinated group to another vaccinated group). In addition, days to parasitemia will be assessed using a Cox Proportional Hazards model and will be displayed using Kaplan-Meier plots.

# Recording and Collection of Data

During the study, the investigator will maintain documentation, including medical records, records detailing the progress of the study for each subject, laboratory reports, CRFs (See Appendices M thru R and LL-MM), signed informed consent forms for each study subject, drug disposition records, correspondence with the IRBs, USAMRMC USRRB/ORP, the study monitor, the SMC and the Sponsor, adverse event reports and information regarding subject discontinuation and completion of the study. All required study data will be clearly and carefully recorded by authorized study personnel. Only designated study site personnel shall record or change study related data. During the study, the investigator will be responsible for the procurement of data and for quality of data collected and recorded. Original observations entered directly onto a CRF are defined as source data.

# Monitoring

Monitoring of records will be conducted internally by the investigators as well as through the continuing review process conducted by the NNMC IRB. In addition an external monitor will provide regulatory and data collection reviews for accuracy and completeness.

The external monitor will review records at appropriate intervals to ensure compliance with the protocol and to verify the accuracy and completeness of data reported, accountability of investigational product, and maintenance of the regulatory file, including the required documentation of appropriate staff training. Monitoring visits, at minimum, will occur at the following intervals: pre-implementation, trial initiation, mid-trial, and closeout.

The Sponsor, the NMRC and NNMC IRBs and the USAMRMC HSRRB will be informed of monitoring visits and reports pertaining to such visits, including final study reports.

# Audit and Inspection

The investigator and study coordinator will be available to respond to reasonable requests and audit queries made by authorized representatives of regulatory agencies. The Sponsor, the NMRC and NNMC IRBs and the USAMRMC HSRRB will be informed of audits/inspections, results and communications pertaining to such audits/inspections.

# Modification to the Protocol

Any modification to the protocol, consent form and/or questionnaires, including changing the PI, must be submitted first to the NNMC IRB for review and approval and then forwarding to the NMRC IRB and BUMED for second level review and approval. In addition major modifications to the research protocol and any modifications that could potentially increase risk to subjects must be submitted to the USAMRMC ORP HSRRB for approval prior to implementation. All other amendments will be submitted to the USAMRMC ORP HRPO for acknowledgement and inclusion in the HRPO study file.

# Adherence to and Changes in Protocol

The investigator will not intentionally deviate from this protocol except in cases of medical emergencies without submitting a request for, and receiving, approval for an amendment to the study. The investigator may deviate from the protocol without prior approval when human risks are neutrally affected or reduced and scientific objectives are not compromised (e.g., postponed immunization by a day to accommodate a volunteer’s schedule) or when the change is necessary to eliminate an apparent immediate hazard to the subject. For deviations that do not meet these requirements, the investigator will notify the NNMC IRB and USAMRMC HSRRB within **5 working days** after the change is implemented.

# Investigational Product Accountability

The investigator must ensure that the investigational product supplies are stored as specified in the SOP’s and in a secured area, with access limited to authorized study personnel. The investigator must maintain accurate records of all investigational products. A record will be maintained that includes the dispensation date, amount of investigational product dispensed, initials and identification number. The investigator in accordance with sponsor’s specifications will document destruction of unused investigational product or its return to the manufacturer.

# Retention of Records

All records pertaining to this protocol will be stored in the Clinical Trials Center of NMRC for the 5 year duration of the study as well as being submitted for storage in the Army Registry. After five years, they may be moved to the Office of Research Administration at 500 Robert Grant Avenue, Silver Spring, MD for archive storage indefinitely. Signed consent form will be stored in a secure location at the Naval Medical Research Center and at the US Army Medical Research and Materiel Command (USAMRMC), Ft. Detrick indefinitely. Relevant institutional review boards will be notified in writing prior to destruction of any research records.

# Disclosure of Information

The study protocol, documentation, data and all other information generated will be held in strict confidence. No information concerning the study or the data will be released to any unauthorized third party. The information developed during the conduct of the study is considered confidential. The data from the study shall be prepared for publication upon completion of the study. Publication will be carried out in such a way as to not permit identification of individual volunteers by readers of the manuscript.

# Medical Care for Research-Related Injuries

All medical care for research-related injuries will be provided at no cost to the volunteer. Minor adverse events will be treated and monitored at the NMRC clinic trials unit. More serious adverse events that warrant hospitalization or extensive medical care will be provided by one of the three mechanisms as outlined in SECNAVINST 3900.39D depending on their status. Because this study is sponsored by the Army Surgeon General, DoD-funded research participants, regardless of healthcare eligibility status, can receive medical care at an Army hospital or clinic free of charge per AR 70-25. If participants pay out-of-pocket for medical care elsewhere for injuries caused by this research study, they should contact the Principal Investigator. If the issue cannot be resolved, contact the U.S. Army Medical Research and Materiel Command (USAMRMC) Office of the Staff Judge Advocate (legal office) at (301) 619-7663/2221. In addition, DoD healthcare beneficiaries and volunteers who receive SECNAV designee status can receive such care for research-related injuries at the National Naval Medical Center (NNMC), or other Navy-affiliated medical institution as well. In the event of emergent medical care all volunteers would be initially managed and stabilized at the NNMC ED per the Support Agreement between NNMC and NMRC for the use of the Clinical Trials Center in building 141 on the NNMC Campus (Appendix NN). Disposition would be determined based on status i.e. DoD healthcare beneficiaries and SECNAV designees (if obtained) could receive care at NNMC or an Army hospital/clinic while non-DoD volunteers would receive further care at an Army hospital/clinic.

#

# Obligations of the Sponsor and the Investigator

This study will be conducted in accordance with all federal regulations regarding the protection of human participants in research including The Nuremberg Code, The Belmont Report, 32 CFR 219 (The Common Rule) and all regulations pertinent to the Department of Defense, the Department of the Navy, the Department of the Army, the Bureau of Medicine and Surgery of the United States Navy and the internal policies for human subject protections and the standards for the responsible conduct of research of the Naval Medical Research Center (NMRC) and US Army Medical Research and Materiel Command.

NMRC holds a Department of Defense/Department of the Navy FWA for human subject protections. In cooperation for those efforts accomplished collaboratively with the Department of Health and Human Services, NMRC holds a Federal Wide Assurance (FWA 00000152) from the Office for Human Research Protections (OHRP). Finally, all NMRC key personnel contributing to or performing human research efforts are certified as having completed mandatory Command human research ethics education curricula and training under the direction of the NMRC Office of Research Administration (ORA) and Human Subjects Protections Program (HSPP).

The investigator agrees to perform the research in accordance with this protocol, the ICH Guideline for Good Clinical Practice (CPMP/ICH/135/95), as well as in conformity with any federal, provincial or local regulations regarding the conduct of clinical studies. The sponsor and investigator must comply with all applicable regulations. In addition, the investigator must follow local and institutional requirements including, but not limited to, investigational product, clinical research, informed consent and Institutional Review Board (IRB) regulations. The sponsor will provide notification to the investigator of protocol and amendment approvals by regulatory authorities when applicable.

Except where the principal investigator's signature is specifically required, it is understood that the term "investigator" as used in this protocol and on CRFs refers to the principal investigator or appropriate study personnel that the investigator designates to perform a certain duty. The principal investigator is ultimately responsible for the conduct of all aspects of the study. Sub-investigators or other appropriate study personnel are eligible to sign for the principal investigator on CRFs.

# Roles and Responsibilities of Study Personnel

The Medical Monitor for this study will be CAPT Daniel Freilich, of NMRC Combat Casualty Care Directorate. The Medical Monitor will be responsible for reviewing all severe or serious and unexpected adverse events and for providing an unbiased written report of the event(s).

The investigators for this study will be involved in all phases of the trial, from volunteer selection to manuscript preparation.

Research associates may be involved in data collection or analysis.

See Appendix HH which details individual roles.

# Protocol Review Process

The protocol will initially receive scientific review through an ad hoc scientific review committee designated by the Office of Research Administration (ORA), NMRC. After the protocol has been deemed acceptable, it will be forwarded to the NMRC ORA for NMRC Institutional Review Board (IRB) review in accordance with the NMRC-WRAIR Memorandum of Understanding concerning joint institution ethical review. Following recommendation for approval, the protocol will be forwarded to the NMRC Commanding Officer. Assuming the protocol is deemed acceptable, the protocol will be forwarded to a joint session of the Navy Bureau of Medicine and Surgery (BUMED) and the USAMRMC HSRRB as well as the National Naval Medical Center IRB (site of clinical trial center) for second level review and final approval action in accordance with a higher agency Memorandum of Understanding. Continuing (annual at minimum) review will be undertaken in accordance with existing IRB regulations.

# Signature of the Investigator

I agree to conduct this clinical study in accordance with the design and specific provisions of this protocol; deviations from the protocol are acceptable only as specified above or with a mutually agreed upon protocol amendment. I also agree to report all information or data in accordance with the protocol; in particular, I agree to report any serious adverse experiences as defined in this protocol.

**Typed Name Signature Date**

__Cindy Tamminga____________ ________________________ _________________________

Principal Investigator

# Appendices (In separate files)

1. Summary of Human Experience
2. Study Agent Preparation SOP for Trial
3. Salivary Gland Scoring
4. Preparation, Staining and Reading of Smears
5. Informed Consent Document for Part A
6. Informed Consent Document for Part B
7. HSP Addendum
8. Active Duty Approval Form for Participation in Part A
9. Active Duty Approval Form for Participation in Part B
10. Assessment of Understanding for Informed Consent: Part A
11. Assessment of Understanding for Informed Consent: Part B
12. SMC Description/Charter
13. Screening Form
14. Eligibility Criteria Worksheet
15. Physical Exam Form
16. Registration Form
17. Volunteer Encounter Form
18. Long Term Annual Telephone Follow-Up Visit Encounter Form
19. Investigator Assurance
20. Volunteer Temperature Log
21. Adverse Event Summary Case Report Form
22. Individual Volunteer Adverse Event Record Sheet
23. Concomitant Medication(s) Summary Case Report Form
24. End of Study Case Report Form
25. Pre-Challenge Assessment Of Understanding
26. Volunteer Encounter Check Sheet
27. NNMC HIV Testing Consent Form
28. Informed Consent Document for Infectivity Control Volunteers
29. Control Group Pre-Challenge Assessment of Understanding
30. Statement of Understanding for SECNAV Designees
31. Suggested Letter to BUMED for SECNAV Designees
32. Active Duty Approval Form for Participation in Part B as Infectivity Control
33. Study Specific HIV Consent
34. Staff Roles and Responsibility Chart
35. HIPAA Authorization for Part A
36. HIPAA Authorization for Part B
37. HIPAA Authorization for Infectivity Controls
38. Post Immunization/Challenge Form
39. Progress Notes
40. NMRC-NNMC Support Agreement for Building 141

**References**
